# Deciphering transcriptomic determinants of the divergent link between PD-L1 and immunotherapy efficacy

**DOI:** 10.1038/s41698-023-00443-3

**Published:** 2023-09-11

**Authors:** Anlin Li, Linfeng Luo, Wei Du, Zhixin Yu, Lina He, Sha Fu, Yuanyuan Wang, Yixin Zhou, Chunlong Yang, Yunpeng Yang, Wenfeng Fang, Li Zhang, Shaodong Hong

**Affiliations:** 1 State Key Laboratory of Oncology in South China, Collaborative Innovation Center for Cancer Medicine, Sun Yat-sen University Cancer Center, Guangzhou, China; 2 Department of Medical Oncology, Sun Yat-sen University Cancer Center, Guangzhou, China; 3 Department of VIP Region, Sun Yat-sen University Cancer Center, Guangzhou, China; 4 Department of Cellular & Molecular Diagnostics Center, Sun Yat-Sen Memorial Hospital, Sun Yat-Sen University, Guangzhou, China; 5 Guangdong Provincial Key Laboratory of Malignant Tumor Epigenetics and Gene Regulation of Sun Yat-Sen University, Guangzhou, China; 6 Department of Oncology, The People’s Hospital of Fengqing, Lincang, China

**Keywords:** Cancer, Predictive markers

## Abstract

Programmed cell death ligand 1 (PD-L1) expression remains the most widely used biomarker for predicting response to immune checkpoint inhibitors (ICI), but its predictiveness varies considerably. Identification of factors accounting for the varying PD-L1 performance is urgently needed. Here, using data from three independent trials comprising 1239 patients, we have identified subsets of cancer with distinct PD-L1 predictiveness based on tumor transcriptome. In the Predictiveness-High (PH) group, PD-L1+ tumors show better overall survival, progression-free survival, and objective response rate with ICI than PD-L1- tumors across three trials. However, the Predictiveness-Low (PL) group demonstrates an opposite trend towards better outcomes for PD-L1- tumors. PD-L1+ tumors from the PH group demonstrate the superiority of ICI over chemotherapy, whereas PD-L1+ tumors from the PL group show comparable efficacy between two treatments or exhibit an opposite trend favoring chemotherapy. This observation of context-dependent predictiveness remains strong regardless of immune subtype (Immune-Enriched or Non-Immune), PD-L1 regulation mechanism (adaptative or constitutive), tumor mutation burden, or neoantigen load. This work illuminates avenues for optimizing the use of PD-L1 expression in clinical decision-making and trial design, although this exploratory concept should be further confirmed in large trials.

## Introduction

Programmed cell death ligand 1 (PD-L1) expression remains the most widely validated, used and accepted biomarker to guide the selection of patients to receive immune checkpoint inhibitors (ICI)^1^. Nevertheless, accumulating data from clinical trials showed a divergent correlate of PD-L1 expression and outcomes across cancer types^1^. Even in non-small-cell lung cancer (NSCLC) where considerable efforts have been made to develop PD-L1 expression as a companion biomarker, most patients with PD-L1+ tumors are non-responders, while some patients with PD-L1- tumors do have durable response to ICI^2^.

To better implement PD-L1 expression as a robust clinical biomarker, previous studies have primarily focused on improving the sensitivity and reproducibility of PD-L1 testing by evaluating the technical and clinicopathological correlation with PD-L1 positivity^3–5^ or developing different means of assessing PD-L1 expression^6–8^. In addition, numerous groups have suggested using PD-L1 expression jointly with tumor mutation burden and CD8 + T cells to predict ICI response^9–11^. Nevertheless, they did not figure out the weakness inherent in PD-L1 expression as a predictive biomarker. While the misinterpretation of PD-L1 expression could result in patients not receiving optimal clinical care, little attempt has been made, to the best of our knowledge^3–11^, to explore the determinants of PD-L1 predictiveness.

A high degree of transcriptional heterogeneity has been characterized within PD-L1+ tumors between cancer types^12^, and PD-L1 can be regulated in response to a variety of inflammatory cytokines and oncogenic signaling pathways^13^, suggesting the immunobiological role of PD-L1 may be susceptible to transcriptional changes in tumor microenvironment (TME). Therefore, we hypothesize that the heterogeneous immune-related transcriptome within and across cancer types might explain the variations in PD-L1 performance observed in clinical trials or real-world data. In addition to immunohistochemistry (IHC)-based PD-L1 expression, this hypothesis may be also applicable to RNA-seq-based PD-L1 gene (*CD274*) expression^7,14^.

Here we put forward two subsets of patients with distinct tumoral PD-L1 predictive capacity in three independent trials: (1) Patients presented optimal predictive value of PD-L1 expression; (2) Patients not only show no PD-L1 predictiveness, but PD-L1+ ones may even demonstrate worse ICI efficacy than PD-L1- ones. Our study provides initial evidence indicating that the predictive capacity of tumoral PD-L1 expression is context-dependent, which can be considerably confounded by tumor transcriptome.

## Results

### Study design and patients

**Fig. 1:**
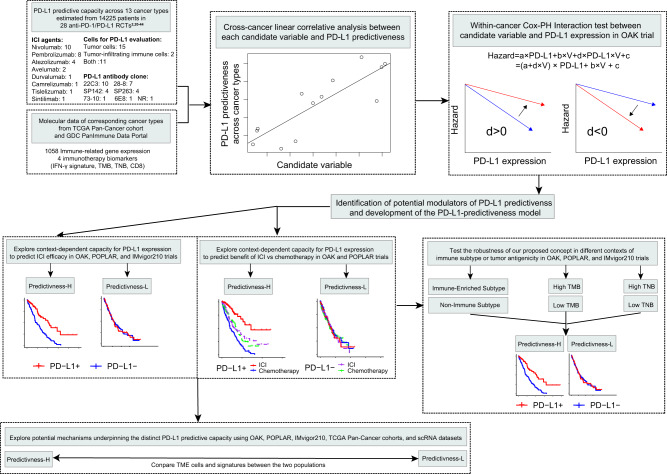
Graphical representation of the study design. RCTs randomized controlled trials, ICI immune checkpoint inhibitor, Cox-PH Cox proportional hazard, IHC immunohistochemistry, TMB tumor mutation burden, TNB tumor neoantigen burden, IFN interferon, OS overall survival, PFS progression-free survival, ORR objective response rate, TME tumor microenvironment, scRNA single-cell RNA-seq.

The study design and summary of data were depicted in Fig. 1 and corresponding sections in the Methods. We analyzed a broad range of data from (1) trial-level data of randomized controlled trials (RCTs) across cancer types; (2) molecular data of corresponding cancer types from TCGA Pan-Cancer cohort and GDC PanImmune Data Portal^15^; and (3) individual-patient level clinical and RNA-seq data of 1239 patients treated with ICI or chemotherapy from three independent clinical trials, including 699 patients from OAK (NCT02008227)^2^, 192 patients from POPLAR (NCT01903993)^2^, and 348 patients from IMvigor210 (NCT02108652 and NCT02951767)^16,17^. The OAK and POPLAR data were formally requested from Genentech at the European Genome-phenome Archive^18^, while the IMvigor210 data were retrieved from the original publication^19^.

To unravel the potential source of PD-L1 predictiveness heterogeneity, we sought to identify immune-related variables capable of explaining the cross-cancer and within-cancer variabilities in predictive value of tumoral PD-L1 expression. We then utilized a transcriptomics-based model to reveal patient subsets with distinct PD-L1 predictive ability in three trials (Fig. 1 and Methods).

### Identification of immuno-modulators of PD-L1 predictiveness

In the cross-cancer identification phase, 28 RCTs^2,20–44^ across 13 cancer types were included. Of the total 14225 patients included, 7591 (53%) received anti-PD-1/PD-L1 therapy and 6634 (47%) received standard-of-care. All of these RCTs assessed single agent in the subsequent-line setting. This eligibility (see Methods) was chosen for two main reasons: (1) to minimize heterogeneity in treatment strategy and patient characteristics; (2) the available immunotherapy trial for individual-patient validation primarily included patients who had received a single agent in subsequent-line settings. More details about baseline characteristics were listed in Fig. 1 and Supplementary table 1.

For a given cancer type, we estimated the ability of PD-L1 expression to stratify survival benefit for ICI versus standard-of-care based on the difference in reduced risk of death/progression between PD-L1+ and PD-L1- subgroups. Hazard ratio (HR) indicates the risk of an event in the treatment group versus that in the control group, and 1- HR can quantify the extent of reduced risk of an event. Hence, the PD-L1 predictiveness for each cancer type could calculated by the HR difference (HRD) = (1 – pooled HR_PD-L1+_) - (1 – pooled HR_PD-L1-_) = HR_PD-L1-_ - HR_PD-L1+_.

The HRD for overall survival (OS) varied considerably by cancer type (Supplementary Fig. 1 and Supplementary table 2). The OS benefit of PD-1/PD-L1 blockade versus standard-of-care treatment is significantly greater in PD-L1+ than PD-L1- tumors for five cancer types, including colorectal cancer (HRD = 1.01, *P interaction* [*P*_*i*_] = 0.03), breast cancer (HRD = 0.42, *P*_*i*_ = 0.03), gastric cancer (HRD = 0.37, *P*_*i*_ = 0.01), melanoma (HRD = 0.31, *P*_*i*_ = 0.05), and esophageal cancer (HRD = 0.23, *P*_*i*_ < 0.01). PD-L1 expression showed insignificant predictiveness in ovarian cancer (HRD = 0.23, *P*_*i*_ = 0.25), head and neck cancer (HRD = 0.09, *P*_*i*_ = 0.62), NSCLC (HRD = 0.08, *P*_*i*_ = 0.21), and bladder cancer (HRD = 0.04, *P*_*i*_ = 0.76). The predictive capacity of PD-L1 lost and showed an opposite trend in kidney clear cell carcinoma, small-cell lung cancer, mesothelioma, and glioblastoma (HRD = -0.02, -0.05, -0.35, and -0.38, respectively). Repeating this analysis using progression-free survival (PFS) showed similar ranking (Supplementary Fig. 2 and Supplementary table 3), and we observed a significantly positive correlation between OS and PFS HRD (*P* = 0.02, Spearman correlation, Supplementary Fig. 3).

To evaluate the association between each candidate variable and cross-cancer PD-L1 predictiveness variation, we calculated the median values of selected variables in TCGA and evaluated their correlations with the HRD derived from RCTs across cancer types. Many studies have justified using this cross-cancer correlative analysis to find predictors of ICI response or immune-related adverse events^11,45,46^. We excluded bladder cancer, ovarian cancer, and head and neck cancer from this analysis due to a substantial heterogeneity between trials (Supplementary Figs. 1, 2 and Supplementary table 4). We first assessed four previously established immunotherapy biomarkers (Interferon [IFN]-γ signature, CD8 score, tumor mutation burden [TMB], and tumor neoantigen burden [TNB])^47^ (Supplementary table 5) and found none of them significantly correlated with HRD for OS or PFS across cancer types (Supplementary Fig. 4), suggesting that the variation in PD-L1 predictiveness between different cancer types is probably not attributed to the level of other predictive biomarkers. Our results are in line with several studies indicating the independence between these biomarkers in predicting immunotherapy efficacy^3,9,10,48^.

Subsequently, we broadened our exploration to 1058 key immune-related genes involved in core TME immune signatures (Supplementary table 6), which was derived from a seminal pan-cancer immune landscape resource^15^. Correlation analysis between the median values of these genes in TCGA and HRD across cancer types resulted in 177 and 108 genes that significantly correlated with OS and PFS HRD across cancer types, respectively, with *P* values less than 0.05; among these, 31 genes were found to be significantly correlated with both OS and PFS HRD (Supplementary Fig. 5 and Supplementary table 7).

In the following within-cancer-level analysis, the 31 genes were subjected to the Cox proportional hazard (Cox-PH) model in atezolizumab-treated patients from OAK. OAK was used as an exploration cohort because it is a phase III study that can provide more confidence. POPLAR and IMvigor210 were later used as validation cohorts. The z-value was calculated to estimate the interaction effects between genes and PD-L1 predictiveness (see Methods). This method has been proven to effectively estimating interaction of any two variables on prognosis^49,50^. Intriguingly, the majority of top hits derived from the gene sets CSR_Activated_15701700 and CHANG_CORE_SERUM_RESPONSE_UP^51,52^, implying that a transcriptional program in fibroblast serum response may affect PD-L1 predictiveness (Supplementary table 8). We identified *CDKN1C* gene as the strongest modulator, with a z-value of 2.50 (*P* = 0.01) and 2.28 (*P* = 0.02) for OS and PFS, respectively. To demonstrate the presence of context-dependent PD-L1 predictive capacity, we developed a PD-L1 predictiveness score (PD-L1 PS) based on a linear regression between *CDKN1C* and OS PD-L1 predictiveness across cancer types (PD-L1 PS = −0.76 × *CDKN1C* + 2.81; Supplementary Fig. 6) and bifurcated patients into Predictiveness-High (PH) and Predictiveness-Low (PL) based on the median value of PD-L1 PS.

### Context-dependent capacity for PD-L1 expression to predict ICI efficacy

Baseline characteristics according to tumoral PD-L1 expression was comparable between PH and PL groups in OAK, POPLAR, and IMvigor210 trials (Supplementary tables 9–11). As expected, using the median value of PD-L1 gene expression for grouping in each trial, there were higher proportions of patients with PD-L1 ≥ 1% by IHC in patients with high (PD-L1-High) versus low (PD-L1-Low) PD-L1 gene expression.

**Fig. 2:**
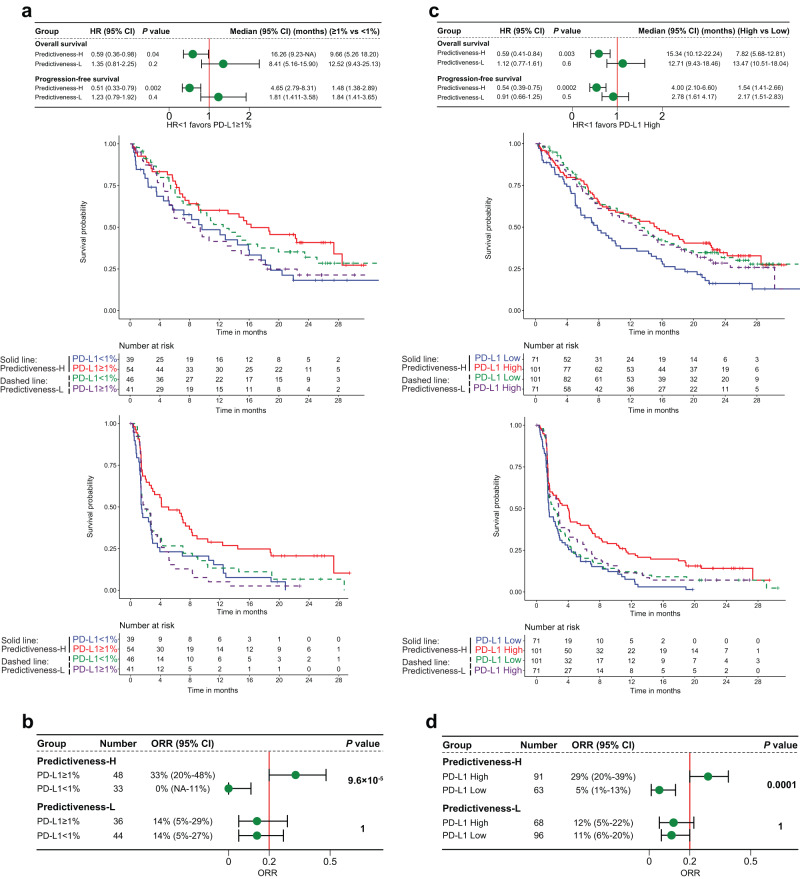
Context-dependent capacity for PD-L1 expression to predict survival and response to immune checkpoint inhibitor in OAK trial. **a**, **b** Overall survival, progression-free survival, and objective response rate with atezolizumab stratified by PD-L1 immunohistochemistry expression based on a cutoff of 1% among Predictiveness-High and Predictiveness-Low patients. **c**, **d** The results of a similar analysis using PD-L1 gene expression. *P* values for Panels a and c indicate log-rank test. *P* values for Panels **b** and **d** indicate Fisher’s exact test. Error bars represent 95% CI. The cutoffs of PD-L1 predictiveness score and PD-L1 gene expression were their median values of atezolizumab-treated patients. HR hazard ratio, CI confidence interval, ORR objective response rate, NA not available.

Among patients receiving ICI from OAK trial, the PH group demonstrated significantly longer OS (HR 0.59, 95% confidence interval [CI] 0.36–0.98, *P* = 0.04, Log-rank test) and PFS (HR 0.51, 95% CI 0.33–0.79, *P* = 0.002, Log-rank test) in PD-L1 ≥ 1% tumors than in PD-L1 < 1% tumors (Fig. 2a). In stark contrast, the PL group showed a trend toward shorter OS (HR 1.35, 95% CI 0.81–2.25, *P* = 0.2, Log-rank test) and PFS (HR 1.23, 95% CI 0.79–1.92, *P* = 0.4, Log-rank test) (Fig. 2a) in PD-L1 ≥ 1% versus PD-L1 < 1% tumors. Additionally, PD-L1 ≥ 1% tumors from PH group had significantly higher objective response rate (ORR) than PD-L1 < 1% tumors (33% vs 0%, *P* = 9.6 × 10^-5^, Fisher’s exact test), whereas PD-L1 ≥ 1% tumors from PL group did not show increased ORR (14% vs 14%, *P* = 1, Fisher’s exact test) (Fig. 2b). Similar results were seen when analyzing PD-L1 gene expression (Fig. 2c, d). We observed a consistent trend when dividing patients into three PD-L1 subgroups, using cutoffs of 1% and 50% for IHC and cutoffs of 25% and 75% percentiles for RNA-seq data (Supplementary Fig. 7). For instance, ORR were 0%, 23.1%, and 45.5% for PH patients with PD-L1 expression of <1%, 1–50%, and ≥50%, respectively. However, PL patients with PD-L1 expression of 50% or higher still showed worse ORR compared to those with PD-L1 < 1% tumors (7.7% vs 13.6%).

**Fig. 3:**
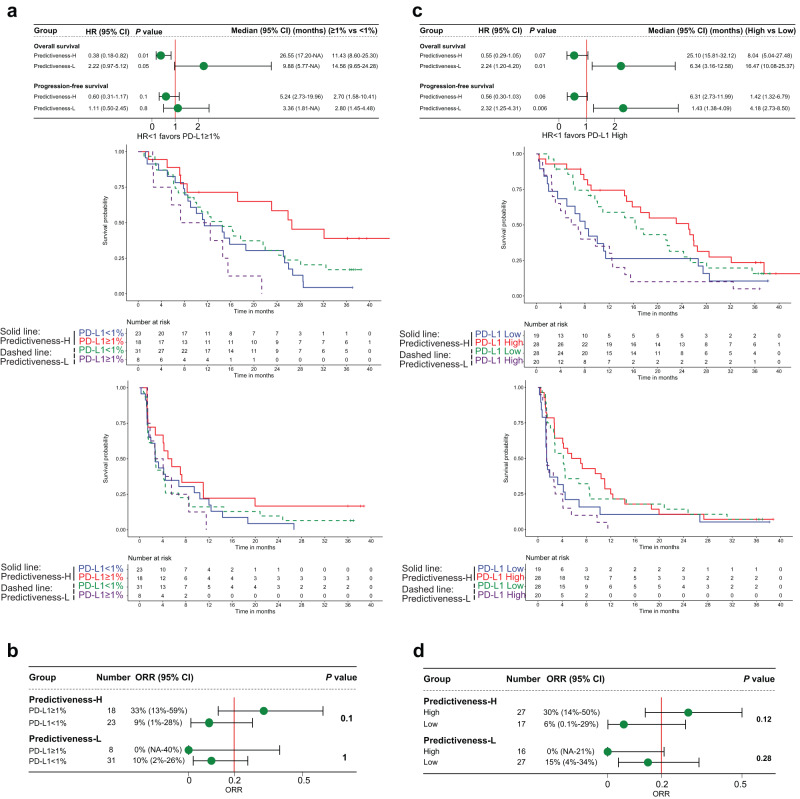
Context-dependent capacity for PD-L1 expression to predict survival and response to immune checkpoint inhibitor in POPLAR trial. **a**, **b** Overall survival, progression-free survival, and objective response rate with atezolizumab stratified by PD-L1 immunohistochemistry expression based on a cutoff of 1% among Predictiveness-High and Predictiveness-Low patients. **c**, **d** The results of a similar analysis using PD-L1 gene expression. *P* values for Panels a and c indicate log-rank test. *P* values for Panels **b** and **d** indicate Fisher’s exact test. Error bars represent 95% CI. The cutoffs of PD-L1 predictiveness score and PD-L1 gene expression were their median values of atezolizumab-treated patients. HR hazard ratio, CI confidence interval, ORR objective response rate, NA not available.

Similar analyses were done in POPLAR trial. PD-L1 ≥ 1% tumors from PH group were associated with improved OS (HR 0.38, 95% CI 0.18–0.82, *P* = 0.01, Log-rank test), PFS (HR 0.60, 95% CI 0.31–1.17, *P* = 0.1, Log-rank test), and ORR (33% vs 9%, *P* = 0.1, Fisher’s exact test) compared to PD-L1 < 1% tumors. However, an opposite trend was seen in PL group for OS (HR 2.22, 95% CI 0.97–5.12, *P* = 0.05, Log-rank test), PFS (HR 1.11, 95% CI 0.50–2.45, *P* = 0.8, Log-rank test), and ORR (0% vs 10%, *P* = 1, Fisher’s exact test) (Fig. 3a, b). The disparity of PD-L1 predictive value between the two groups was also striking when analyzing PD-L1 gene expression (Fig. 3c, d). In PL group, PD-L1-High tumors had significantly worse OS (HR 2.24, 95% CI 1.20–4.20, *P* = 0.01, Log-rank test) and PFS (HR 2.32, 95% CI 1.25–4.31, *P* = 0.006, Log-rank test), and showed a trend towards lower ORR (0% vs 15%, *P* = 0.28, Fisher’s exact test), as compared with PD-L1-Low tumors. Similar findings were derived when analyzing three PD-L1 expression levels (Supplementary Fig. 8). The ORR were 8.7%, 18.2% and 57.1% in PH group, and those in PL group were 9.7%, 0%, and 0% for patients with PD-L1 expression of <1%, 1–50%, and ≥50%, respectively. Similar to OAK, the Cox-PH interaction test between CDKN1C and PD-L1 expression was significant (OS: z-value = 3.09, *P* = 0.002; PFS: z-value = 3.27, *P* = 0.001; Supplementary table 12).

**Fig. 4:**
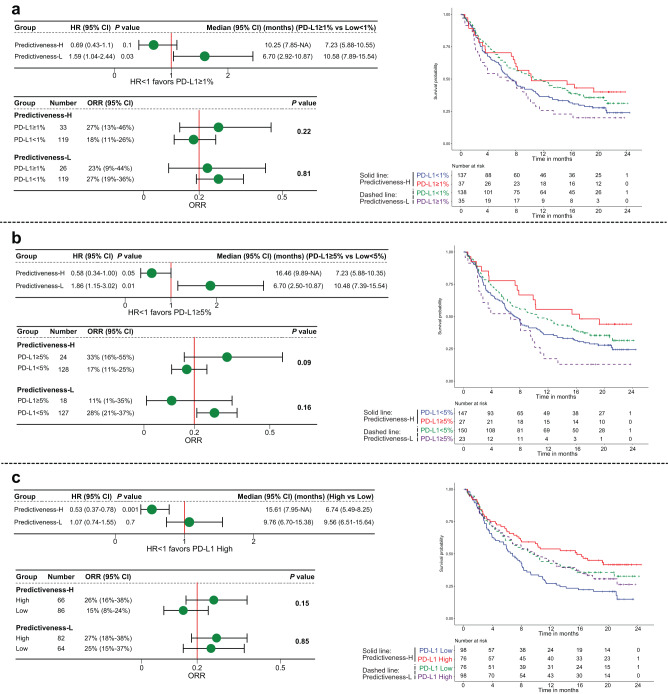
Context-dependent capacity for PD-L1 expression to predict survival and response to immune checkpoint inhibitor in IMvigor210 trial. **a** Overall survival and objective response rate with atezolizumab stratified by PD-L1 immunohistochemistry expression based on a cutoff of 1% among Predictiveness-High and Predictiveness-Low patients. **b** The results of a similar analysis using a cutoff of 5%. **c** The results of a similar analysis using PD-L1 gene expression. *P* values for survival analyses indicate log-rank test. *P* values for response analyses indicate Fisher’s exact test. Error bars represent 95% CI. The cutoffs of PD-L1 predictiveness score and PD-L1 gene expression were their median values of atezolizumab-treated patients. HR hazard ratio, CI confidence interval, ORR objective response rate, NA not available.

To prove that this phenomenon is not restricted to NSCLC, we further applied the PD-L1 PS in 348 patients with bladder cancer from IMvigor210 trial. In PH group, PD-L1 ≥ 1% tumors improved OS (HR 0.69, 95% CI 0.43–1.10, *P* = 0.1, Log-rank test) and ORR (27% vs 18%, *P* = 0.22, Fisher’s exact test) as compared with PD-L1 < 1% tumors, whereas the trends were reversed for OS (HR 1.59, 95% CI 1.04–2.44, *P* = 0.03, Log-rank test) and ORR (23% vs 27%, *P* = 0.81, Fisher’s exact test) in PL group (Fig. 4a). We observed more noticeable results when using 5% as the cutoff for PD-L1 positivity (Fig. 4b). The HR of OS (HR 0.58, 95% CI 0.34–1.00, *P* = 0.05, Log-rank test) and ORR (33% vs 17%, *P* = 0.09, Fisher’s exact test) favored PD-L1+ tumors in PH group, while the HR of OS (HR 1.86, 95% CI 1.15–3.02, *P* = 0.01, Log-rank test) and ORR (11% vs 28%, *P* = 0.16, Fisher’s exact test) favored PD-L1- tumors in PL group. These results were markedly different from the original publication of IMvigor210^19^, which showed no difference in ORR between PD-L1 subgroups based on a cutoff of 1% or 5%. Similar results were seen when analyzing PD-L1 gene expression by two or three levels (Fig. 4c and Supplementary Fig. 9). While there was a clear monotonic relationship between an increasing PD-L1 gene expression and ORR in PH group (10.4%, 19.4%, and 32.4% for low, intermediate, and high expression), the trend was reversed in PL group (27.6%, 26.3%, and 24.3% for low, intermediate, and high expression). Similar to OAK and POPLAR, the Cox-PH interaction test between CDKN1C and PD-L1 expression was significant (OS: z-value = 2.57, *P* = 0.01; Supplementary table 12).

### Context-dependent capacity for PD-L1 expression to predict benefit of ICI over chemotherapy

**Fig. 5:**
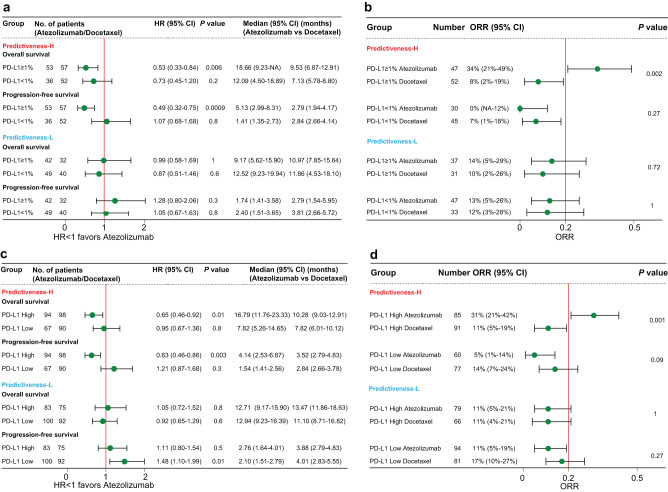
Context-dependent capacity for PD-L1 expression to predict benefit of immune checkpoint inhibitor over chemotherapy in OAK trial. **a**, **b** Overall survival, progression-free survival, and objective response rate with atezolizumab versus docetaxel stratified by PD-L1 immunohistochemistry expression based on a cutoff of 1% among Predictiveness-High and Predictiveness-Low patients. **c**, **d** The results of a similar analysis using PD-L1 gene expression. *P* values for Panels a and c indicate log-rank test. *P* values for Panels b and d indicate Fisher’s exact test. Error bars represent 95% CI. The cutoffs of PD-L1 predictiveness score and PD-L1 gene expression were their median values of total intention-to-treat patients. The results of a similar analysis in POPLAR trial were shown in Supplementary Fig. 14. HR hazard ratio, CI confidence interval, ORR objective response rate, NA not available.

We interrogated whether the capacity of tumoral PD-L1 expression in predicting efficacy of ICI versus chemotherapy was also influenced by our proposed PD-L1 predictiveness. Among PH patients from OAK trial, PD-L1 ≥ 1% tumors exhibited significantly improved OS (HR 0.53, 95% CI 0.33–0.84, *P* = 0.006, Log-rank test), PFS (HR 0.49, 95% CI 0.32–0.75, *P* = 0.0009, Log-rank test), and ORR (34% vs 8%, *P* = 0.002, Fisher’s exact test) with atezolizumab versus docetaxel, whereas PD-L1 < 1% tumors showed insignificant findings for these outcomes (Fig. 5a, b and Supplementary Fig. 10). However, PD-L1 ≥ 1% tumors from the PL group showed comparable efficacy between two treatments or presented an opposite trend towards benefit with docetaxel (Fig. 5a, b and Supplementary Fig. 11). We obtained similar results when using PD-L1 gene expression (Fig. 5c, d and Supplementary Figs. 12, 13).

Repeating these analyses in POPLAR trial also yielded contrasting findings between the PH and PL groups (Supplementary Figs. 14–18). Although PD-L1 gene expression has been associated with enhanced survival benefit of atezolizumab versus docetaxel in the previous publication of POPLAR trial^14^, we found a completely reverse trend for PL patients. The HR of OS (HR 1.63, 95% CI 0.82–3.23, *P* = 0.2, Log-rank test) and PFS (HR 2.30, 95% CI 1.16–4.56, *P* = 0.01, Log-rank test) favored docetaxel in PD-L1-High tumors, while the HR of OS (HR 0.67, 95% CI 0.38–1.19, *P* = 0.2, Log-rank test) and PFS (HR 0.76, 95% CI 0.44–1.31, *P* = 0.3, Log-rank test) favored atezolizumab in PD-L1-Low tumors (Supplementary Fig. 18).

### Context-dependent PD-L1 predictiveness maintains regardless of immune subtype

The upregulation of PD-L1 in TME is mainly driven by IFN-γ, representing a negative feedback event to inhibit the adaptive immune response^13,53^. On the other hand, many oncogenic pathways can lead to constitutive PD-L1 expression, which may not provide predictive value for ICI due to the absence of a pre-existing anti-tumor immunity^13,53^.

We thus asked whether the observed distinct predictiveness of PD-L1 between PH and PL groups stem from differences in PD-L1 upregulation mechanism. Analyses were performed in the three trials, separately. Unexpectedly, both PH and PL groups demonstrated comparably increasing levels of three inflammatory biomarkers, including IFN-γ signature, T-cell inflamed gene expression profile (GEP), and CD8 score, in PD-L1-High versus PD-L1-Low tumors (Supplementary Fig. 19). These data indicate that a T-cell inflamed TME dominates PD-L1 upregulation regardless of PD-L1 predictiveness. This notion was further supported by the observation that PD-1-high CD8 + T cells showed a significant correlation with PD-L1 across the PH and PL groups in each trial (Supplementary Fig. 20). More convincingly, we examined the enrichment levels of several canonical pathways^13,53^ involved in adaptive or constitutive PD-L1 upregulation, and found that the strength of correlation between PD-L1 and adaptive immune evasion pathways was markedly greater than that between PD-L1 and constitutive pathways in each trial, irrespective of PD-L1 predictiveness (Supplementary Fig. 21).

We subsequently predicted relative TME cell proportions in each trial using Kassandra, which showed better single-cell-level accuracy and stability than previous tools^54^. Unsupervised clustering of cells identified two immune subtypes in both PH and PL groups, with Immune-Enriched Subtype harboring abundant lymphocytes in contrast to predominantly tumor cells and relative paucity of immune cells in Non-Immune Subtype (Supplementary Figs. 22, 23). There were higher proportions of Immune-Enriched Subtype tumors in PD-L1-High versus PD-L1-Low tumors, irrespective of PD-L1 predictiveness (Supplementary table 13). Importantly, both Immune-Enriched and Non-Immune subtypes retained an expected disparity in PD-L1 predictiveness between PH and PL tumors (Supplementary Fig. 24), indicating that TME components beyond lymphocyte infiltration can influence the PD-L1 predictiveness.

In addition to the PD-1/PD-L1 axis, expressions of other immune checkpoints such as CTLA4, TIM3, and LAG3, may contribute to PD-L1-independent adaptive resistance and relate to reinvigoration potential of dysfunctional T cells^55^. We found that expression of PD-L1 in PH patients and that in PL patients exhibited a similar correlation pattern with other immune checkpoints in each trial (Supplementary Fig. 25). Thus, checkpoint-driven T-cell exhaustion was also unlikely to be responsible for driving distinct PD-L1 predictiveness.

Similar to PD-L1 expression, we also observed diminished abilities for IFN-γ signature, GEP, or CD8 score to predict ICI benefit in PL group in each trial (Supplementary Fig. 26), probably because of their tight relationships with PD-L1 expression. Similarly, while Immune-Enriched Subtype tumors were associated with significantly longer OS when compared to Non-Immune Subtype tumors in PH group, the magnitude of increased benefits was smaller or reversed in PL group (Supplementary Fig. 27).

Collectively, in this section, we demonstrated that tumoral PD-L1 predictiveness is at least partially independent of factors including PD-L1 regulation mechanism (adaptive or constitutive), immune subtype (Immune-Enrich or Non-Immune), and expression of immune checkpoints other than PD-L1. In addition, not only the predictive value of PD-L1 expression, but also that of other inflammatory biomarkers can be influenced by our proposed PD-L1 predictiveness.

### Context-dependent PD-L1 predictiveness maintains regardless of tumor antigenicity

TMB and TNB are indicative of tumor immunogenicity and they predict ICI response independently of PD-L1 expression^9,10^. We sought to evaluate the robustness of our proposed PD-L1 predictiveness in different conditions of TMB or TNB. The OAK and IMvigor210 trials provided data for TMB, while only the IMvigor210 trial provided data for TNB. We used a threshold value of 16 mut/Mb to classify patients into high TMB or low TMB group, and the median level for the TNB groupings. In each trial, the phenomenon of context-dependent PD-L1 predictiveness remained strong in each subgroup by TMB (Supplementary Fig. 28) or TNB (Supplementary Fig. 29). Unlike the aforementioned inflammatory biomarkers, the predictive values of TMB and TNB were not affected by PD-L1 predictiveness (Supplementary Figs. 30, 31). Therefore, TMB and TNB that capture distinct feature of tumor antigenicity may be used to predict ICI response for PL patients.

### Stomal cells might underlie the context-dependent PD-L1 predictiveness

**Fig. 6:**
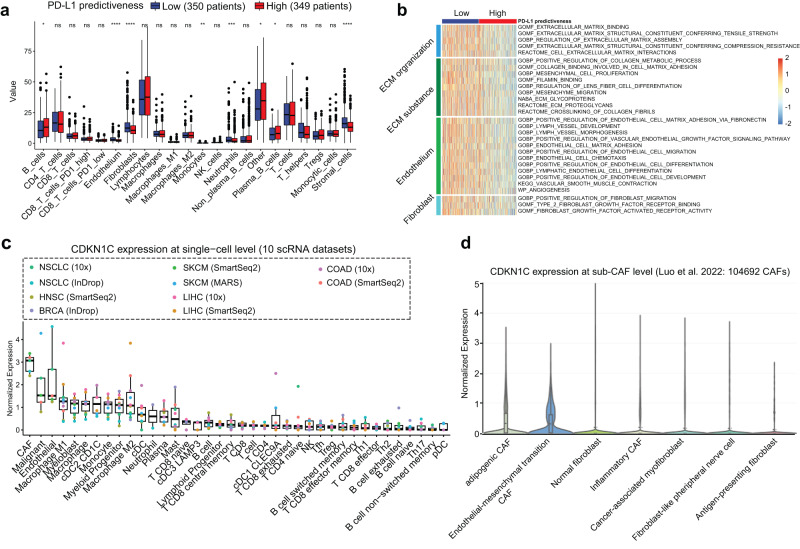
Exploration of potential mechanisms underpinning the distinct PD-L1 predictiveness. **a** The fraction of Kassandra-based cells in Predictiveness-High group versus Predictiveness-Low group in OAK trial. The results of a similar analysis in POPLAR and IMvigor210 trials were shown in Supplementary Fig. 32. The cutoff of PD-L1 predictiveness score was the median value of total intention-to-treat patients in each trial. The “Stromal cells” were calculated as a sum of “Endothelium” and “Fibroblasts” values. The “Other” indicated all cells not deconvolved by Kassandra, mainly including malignant cells and benign epithelial cells. The horizontal line in the boxes represents the median value. The bottom and top of the boxes are the lower and upper quartiles. The whiskers encompass 1.5 times the interquartile range. *P* value indicates Wilcoxon rank-sum test. The range of P values is labeled with asterisks. *P < 0.05; **P < 0.01; ***P < 0.001; ****P < 0.0001. **b** Association of PD-L1 predictiveness score with pathways related to stromal activity in OAK trial. Data were represented as the z-score of population enrichment across each trial. The results of a similar analysis in POPLAR and IMvigor210 trials were shown in Supplementary Fig. 33. **c** CDKN1C expression in single-cell level based on a meta-cohort of 10 single-cell RNA-seq datasets. The horizontal line in the boxes represents the median value. The bottom and top of the boxes are the lower and upper quartiles. The whiskers encompass 1.5 times the interquartile range. **d** CDKN1C expression in fibroblast subpopulation at single-cell level based on the largest single-cell fibroblast database to date (https://gist-fgl.github.io/sc-caf-atlas/#) [14]. ECM extracellular matrix, CAF cancer-associated fibroblast, cDC conventional dendritic cells, pDC Plasmacytoid dendritic cells, NSCLC non-small-cell lung cancer, SKCM melanoma, COAD colon cancer, HNSC head and neck cancer, BRCA breast cancer, LIHC liver cancer.

Prompted by findings in our previous section showing that PD-L1 predictiveness may be obscured by the pathophysiological status of fibroblast (Supplementary table 8), we thus directly compared cell components between PH and PL groups in each trial. As expected, there were higher levels of stromal cells, including fibroblasts and endothelium, in PL tumors than in PH tumors (Fig. 6a and Supplementary Fig. 32). We further found that PL tumors were enriched for stroma-related activities, such as extracellular matrix organization, collagen metabolic process, fibroblast migration, and endothelial cell-matrix adhesion (Fig. 6b and Supplementary Fig. 33). To test the generalizability of the association between PD-L1 predictiveness with T-cell infiltration, we expanded our analysis to all TCGA cancer types. The results showed a significantly negative correlation of PD-L1 PS with fibroblast, endothelial cell, and stromal score in approximately half of cancer types (Supplementary Fig. 34).

Next, we examined the expression of the CDKN1C gene (PD-L1 PS gene) at the single-cell level across several cancer types in 10 single-cell RNA-seq datasets from Ru et al. ^56^. We found that cancer-associated fibroblasts (CAFs) are the main TME cells expressing CDKN1C (Fig. 6c). Using the largest single-cell atlas of CAFs^57^, we further observed higher expression of CDKN1C in adipo-genic CAFs (CAF-adi) and CAFs that exhibit endothelial-mesenchymal transition (CAF-EndMT) than in other subsets of CAFs (Fig. 6d). CAF-EndMT exhibited transcriptional pattern of both fibroblasts and endothelial cells^57^, which was consistent with previous enrichment analyses of TME cells and pathways. Although providing a definitive mechanism will require further studies in controlled experimental systems, these data collectively pointed towards the potentially biological role of stromal cells in determining PD-L1 predictiveness.

## Discussion

Despite accumulating evidence showing the controversial predictiveness, PD-L1 expression is currently the most widely used and accepted biomarker to select patients to receive anti-PD-1/PD-L1 therapies, and four IHC assays have been approved by Food and Drug Administration as companion diagnostics^1^. Improved understanding of factors underlying the variability in PD-L1 predictiveness is essential for precision immunotherapy. This study provides initial evidence indicating that the predictive capacity of PD-L1 expression measured by IHC or RNA-seq is context-dependent. PH group exhibited an evident improvement of ICI efficacy for PD-L1+ versus PD-L1- tumors, but the predictive value of PD-L1 expression vanished or outcomes even trended towards benefit for PD-L1- tumors in PL group. Clinical decision based on PD-L1 expression might be ineffective or even harmful for PL patients who account for approximately half of patients in the three trials we analyzed, cautioning against indiscriminately using PD-L1 expression to guide ICI treatment.

The present analysis found that only five out of 13 assessed cancer types showed a sufficiently strong association between PD-L1 status and OS benefits from anti-PD-1/PD-L1 therapies versus standard-of-care. Four cancer types showed an opposite trend towards better efficacy in PD-L1- tumors. These data indicates that clinical trials may fail if investigators blindly use PD-L1 positivity to limit patient enrollment or define the target population for assessing primary endpoint, especially in cancer types with a substantial number of PL patients. Notably, we found a lack of PD-L1 predictiveness for NSCLC, where an increasing OS with anti-PD-1/PD-L1 was seen in both PD-L1+ and PD-L1- patients. This aligned with the final results of OAK and POPLAR trials which showed a survival benefit of atezolizumab over docetaxel regardless of PD-L1 expression^2^. The 4-year OS rates were comparable between PD-L1+ and PD-L1- tumors in OAK (17% vs 14%) and POPLAR (15% vs 15%)^2^. Thus, PD-L1 expression can neither achieve consistent predictive value across cancer types nor precisely determine whether or not a patient could derive long-term survival benefits within a cancer type.

Following the predominant focus on technical challenges of PD-L1 testing^1^, additional effort would be required to explore which groups of patients could benefit from treatment decision based on PD-L1 stratification and which populations need additional biomarkers to guide precise selection. In this regard, our work highlights the importance of individualizing application of PD-L1 expression in different TME contexts of PD-L1 predictiveness. Our findings caution that many patients’ treatment decisions might be misguided by PD-L1 expression due to low PD-L1 predictiveness. Using 1% as PD-L1 positivity cutoff, the risk of death was 35% higher in PD-L1+ than in PD-L1- tumors among PL patients in OAK trial, and that was 122% in POPLAR trial. In PL patients from IMvigor210 trial, PD-L1+ tumors were associated with an increased risk of death by 59% and 86% compared with PD-L1- tumors when using cutoffs of 1% and 5%, respectively.

Intriguingly, the PL group also demonstrated low predictive values for other well-established inflammatory biomarkers, including IFN-γ signature, GEP, and CD8 score. These findings aligned with current evidence that revealed a double-edged role of IFN-γ signaling in association with response to ICI^58–60^. Contrary to the common concept that IFN-γ is necessary for anti-tumor immune response and is linked to the efficacy of ICI, enhancing IFN-γ signaling can lead to resistance^58^ or hyper-progression^59^ to ICI in certain contexts. Our work and these studies jointly emphasize the importance of delineating the biological heterogeneity of currently standard biomarkers, such as PD-L1 expression.

The PD-L1 predictiveness identified here is unaffected by immune subtype, PD-L1 regulation mechanism, and tumor antigenicity. Rather, our preliminary mechanistic exploration suggests that the variation of PD-L1 predictive ability may be attributable partly to distinct stromal quantity and quality. We observed a higher level of stromal cells (fibroblasts and endothelium) and stroma-related pathways in PL than in PH tumors. Moreover, the analysis of single-cell data confirmed high expression of CDKN1C in CAFs, especially in CAF-adi and CAF-EndMT subsets. CAFs predominate tumor stroma and consist of highly heterogeneous subpopulations that can exert immunosuppressive (ICI-resistant phenotype) or immunostimulatory (ICI-responsive phenotype) effect dependent on the certain TME context^61,62^. Given the plasticity of CAFs, we propose two hypotheses to explain the divergent PD-L1 predictiveness. Firstly, CDKN1C+ CAFs within the PD-L1 + TME may transition into a suppressive state through interactions with PD-L1+ tumor or immune cells, as well as other cells commonly found in an inflamed TME, such as plasma cells and dendritic cells. As a result, these CAFs can curtail the immune response and counteract the positive predictive capacity of PD-L1. Alternatively, CDKN1C+ CAFs may undergo phenotypic changes to become immunosuppressive in response to altered signaling (e.g., cytokines) following the blockade of PD-1/PD-L1 in the PD-L1 + TME. To validate these hypotheses, future mechanistic studies may evaluate single-cell-level transcriptomic profiles from baseline and on-treatment samples of both PD-L1+ and PD-L1- cases with matched clinical information of ICI efficacy.

One of the major limitations of this work is that the current analyses make it difficult to provide a definitive mechanism underlying the distinct PD-L1 predictiveness. Additionally, the study was exploratory in nature, and the technical and statistical pipelines are not perfect. Raw reads and count data, except for the IMvigor210 study (the distribution of counts is shown in Supplementary Fig. 35), were unavailable, increasing the risk that our methods are susceptible to technical factors such as read depth, tumor fraction, and other aspects of library preparation or in-silico factors. Considering the limited number of included cancer types renders low power to detect differences during cross-cancer correlative analysis, adjustments for multiple comparisons were not made.

Nevertheless, our study demonstrated that a proportion of patients’ treatment decisions might be misguided by PD-L1 expression due to TME, which may prove helpful for selecting suitable candidates for PD-L1 testing. Importantly, we confirmed the consistency of clinical and molecular observations in three large clinical trials involving over 1200 patients, which adds confidence to the results. Taken together, further studies are necessary to determine whether our proposed concept of PD-L1 predictiveness and associated biological aspects are generalizable to other unexplored treatment strategies, patient characteristics, and PD-L1 scoring cutoffs.

In conclusion, our work reveals previously unappreciated context-dependent capacity for PD-L1 expression to predict benefit of ICI, which fill an important gap in our understanding of the varying PD-L1 performance. Classifying PD-L1 predictiveness based on tumor transcriptome information might be a promising strategy to guide the personalized application of PD-L1 expression in predicting immunotherapy outcomes, although this concept requires further confirmation.

## Methods

### Search strategy, selection criteria, and data extraction for randomized controlled trials

We performed a systematic literature search of PubMed, EMBASE, MEDLINE, and Scopus to identify phase 2 and 3 randomized controlled trials (RCTs) published prior to August 16, 2022. The search terms included “PD-1”, “PD-L1”, “nivolumab”, “atezolizumab”, “pembrolizumab”, “cemiplimab”, “avelumab”, “durvalumab”, “tislelizumab”, and “randomized”. Only studies published in English were considered. References from review articles and included studies were reviewed to ensure completeness. We included only the most updated or final results of RCTs when several publications of the same trial were identified.

To be eligible, randomized trials had to assess PD-1 or PD-L1 inhibitors versus standard-of-care in subsequent-line setting, and had to have data available for the hazard ratio (HR) and 95% confidence interval (CI) for death or progression in PD-L1+ and/or PD-L1- subgroups. The scoring method and threshold of PD-L1 immunohistochemistry (IHC) positivity were eligible in any of the followings: tumor proportional score (TPS) of 1%, combined positive score (CPS) of 1, tumor cells (TC) of 1%, tumor-infiltrating immune cells (IC) of 1%, or other means used to define PD-L1 positivity by investigators. We excluded studies that compared anti-PD-1/PD-L1 agents with placebo, studies that presented survival curves without reporting HRs and 95% CIs, and studies that only evaluated combination treatment. We also excluded studies that only reported the results of either the PD-L1+ or PD-L1- subgroups if, in a particular cancer type, only one trial was available. This is because, in such cases, the PD-L1 predictive capacity cannot be evaluated for that cancer type.

From each study, we extracted the name of study, year of publication, cancer type, study phase, line of therapy, target of inhibitor, study drug, PD-L1 antibody clone, PD-L1 scoring method, cell subset for evaluation, number of patients, and HR and CI according to PD-L1 expression status.

### Collection of molecular data from TCGA

We derived clinical (https://tcga-pancan-atlas-hub.s3.us-east-1.amazonaws.com/download/Survival_SupplementalTable_S1_20171025_xena_sp) and transcripts per million (TPM)-normalized RNA-seq data of TCGA Pan-Cancer cohort (https://toil.xenahubs.net/download/tcga_RSEM_gene_tpm.gz) from UCSC Xena browser. Normal samples coded with “11” were removed. The Toil^63^ was used to perform the RNA-seq pipeline. CutAdapt was employed to remove extraneous adapters, while STAR was utilized for alignment and read coverage, and RSEM was employed for expression quantification. The STAR and RSEM indexes were constructed using the HG38 reference genome and Gencode’s v23 comprehensive CHR annotation file.

Tumor mutation burden (TMB) was calculated using nonsynonymous SNP and INDEL mutations per megabase via whole exome sequencing. The data were downloaded from the mutation-load_updated.txt file located at the GDC PanImmune Data Portal (https://api.gdc.cancer.gov/data/ff3f962c-3573-44ae-a8f4-e5ac0aea64b6). The MC3 project employed multiple rules to remove poor quality samples^64^. Tumor neoantigen burden (TNB) were downloaded from TCGA_pMHC_SNV_sampleSummary_MC3_v0.2.8.CONTROLLED_170404.tsv located at GDC PanImmune Data Portal (https://api.gdc.cancer.gov/data/0d3ee0a7-0557-447b-9ada-bc7838d1effb). The pipeline for generating TNB has been described in details in a previous publication^15^. Briefly, the MC3 variant file (mc3.v0.2.8.CONTROLLED.maf) was used to extract somatic nonsynonymous coding single nucleotide variants based on following filters in “PASS,” “wga,” “native_wga_mix”; NCALLERS > 1; barcode in whitelist where do_not_use=False; Variant_Classification = “Missense_Mutation”; and Variant_Type = “SNP.” Then, potential neoantigenic peptides were identified using NetMHCpan v3.0 based on HLA types derived from RNA-seq via OptiType. Peptides containing amino acid mutations were regarded as possible antigens if they showed a predicted binding to autologous MHC.

### Methods of OAK, POPLAR, and IMvigor210 trials

The OAK and POPLAR trial data have been granted permission by Genentech/Roche, while the IMvigor210 data were publicly available (see Data Availability section). Detailed descriptions of the eligibility criteria and recruitment methods for OAK^2^, POPLAR^2^, and IMvigor210^16,17^ trials have been reported previously. Briefly, the randomized, multicenter, open-label phase II POPLAR and phase III OAK trials compared atezolizumab and docetaxel among non-small-cell lung cancer (NSCLC) patients who progressed following platinum-based chemotherapy. The single-arm, phase II IMvigor210 trial examined atezolizumab among patients with locally advanced or metastatic urothelial bladder cancer across first-line and second-line settings. These trials were done in accordance with the Declaration of Helsinki and approval was obtained from local ethics committees.

All patients were available for PD-L1 gene expression data, while 361 (51.6%), 155 (80.7%), and 347 (99.7%) patients in three trials, respectively, had available PD-L1 IHC information. PD-L1 expression on tumor cells was stained by IHC using formalin fixed paraffin embedded (FFPE) tumor tissues, and the expression level was scored as the sum of PD-L1+ tumor cells as a proportion of the total number of viable tumor cells. The OAK trial used 22C3 assay and the POPLAR and IMvigor210 trials used SP142 assay.

The procedures of RNA-seq for the OAK^18^, POPLAR^18^, and IMvigor210^19^ trials have been published previously. In these trials, RNA extraction was performed on tumor samples with ≥20% tumor cell content, of which >75% demonstrated ≥45% tumor purity. RNA was extracted from the macro-dissection-marked H&E images (High Pure FFPET RNA Isolation Kit, Roche). All transcriptome profiles were generated using TruSeq RNA Access technology (Illumina). Ribosomal RNA reads were removed by aligning RNA-seq reads, followed by alignment of remaining reads to the NCI Build 38 human reference genome using GSNAP version 2013-10-10, allowing for up to two mismatches per 75 base sequences. Transcript annotation was based on the Ensembl genes database (release 77). Gene expression levels were quantified by calculating the number of reads mapped to the exons of each RefSeq gene in a strand-specific manner, utilizing the R package Genomic Alignments. All RNA-seq data were normalized as log_2_(TPM + 0.001).

The OAK and IMvigor210 trials provided data for TMB, while only the IMvigor210 trial provided data for TNB. Tissue TMB was evaluated using FFPE samples through comprehensive genomic profiling with FoundationOne. TMB ≥ 16 mut/Mb was used as a cutoff since it has been validated in a prospective study using FoundationOne testing^65^. In the OAK study, TMB was defined as the number of somatic, coding SNVs and indels detected at an allele frequency of ≥5%, after excluding known and likely oncogenic driver events and germline SNPs. Any artifacts were removed by comparing to a database comprised of normal, healthy FFPE tissue and computational filtering for strand bias. The details of the TMB and TNB pipeline used in the IMvigor210 study have been published^19^. Briefly, TMB was calculated based on the number of SNVs and indels detected in coding regions, excluding known and predicted germline alterations as well as known likely somatic variants. To identify expressed mutations, we tallied RNA-seq alignments for somatic mutations found in exome data using VariantTools’ tallyVariants function. We then predicted the neoantigen potential of each mutation by identifying the HLA genotypes of the subjects and assigning the optimal HLA-neoepitope pair across all HLA alleles and 8-11 mer peptides containing the mutation, based on the minimum IC50 values predicted by NetMHCcons.

### Identifying immuno-modulators of PD-L1 predictiveness and constructing the PD-L1 predictiveness score

A two-step procedure was applied to identify modulators of PD-L1 predictiveness, with the intent of evaluating the association of each candidate variable with cross-cancer and within-cancer variabilities in predictive value of PD-L1 expression. The variables for screening included four biomarkers previously associated with ICI response (Interferon [IFN]-γ signature, CD8 score, TMB, and TNB)^47^, and 1058 immune-related genes from 58 gene sets that were previously used for scoring and clustering of the pan-cancer immune landscape^15^.

In the first step, for a given cancer type, we estimated the ability of PD-L1 expression to stratify benefits of overall survival (OS) and progression-free survival (PFS) for ICI versus standard-of-care by computing hazard ratio difference (HRD) based on RCT data, which was defined as the pooled HR of the PD-L1- subgroup minus that of the PD-L1+ subgroup. Then, we calculated the median values of selected variables of the corresponding cancer types in TCGA and evaluated their correlations with HRD across cancer types. The Spearman rank correlation coefficient *Rs* was used to estimate how well the cross-cancer PD-L1 predictiveness variance could be explained by a variable. Many studies have justified using this cross-cancer correlative analysis to find predictors of ICI response or immune-related adverse events^11,45,46^.

Next, variables that significantly correlated with both OS and PFS HRD entered the second step, where an interaction test was performed to rank variables by their interaction effects with PD-L1 predictiveness within a cancer type. This method has been proven to effectively estimating interaction of any two variables on prognosis^49,50^. We assume a multivariate Cox proportional hazard (Cox-PH) regression model: Hazard = a × PD-L1+b × V + d× PD-L1 × V + c. Hazard is the hazard of death or progression. PD-L1 and V represent the expression level of PD-L1 gene expression and that of a candidate variable, respectively (e.g., 0 indicates value <median, 1 indicates value ≥ median). The interaction term of PD-L1 and V is PD-L1 × V, and the association slope between PD-L1 and Hazard is a + d× V. A positive or negative d value represents that a high V will decrease or increase the beneficial association between PD-L1 expression and survival benefit of ICI. Identification of modulator was based on the Wald test statistic for testing a null interaction effect, d = 0. To quantify the extent of interaction effect, a standardized Wald test z-value for each variable was calculated by the coefficient d divided by its standard error.

Finally, the V with the highest z-value was used to generate the PD-L1 predictiveness score (PD-L1 PS) based on the linear regression model between V and OS HRD across cancer type via the R package caret (version 6.0-94): PD-L1 PS = α ×V + β, using a standard leave-one-out cross-validation method.

### Estimation of tumor microenvironment (TME) signature

The gene sets used in this study were summarized by Supplementary table 14. For each dataset, before calculating the enrichment level of each TME signature, we applied quantile normalization to standardize the TPM data using the R package preprocessCore (version 1.62.1). Additionally, we normalized the expression values of each gene by subtracting the average expression level among all samples. The levels of IFN-γ signature and T-cell inflamed gene expression profile were calculated as the average expression of the genes obtained from Ayers et al. ^66^. CD8 score was calculated as the average expression of CD8A and CD8B. Gene set variation analysis was conducted with gene sets from the MSigDB database using the R package GSVA (version 1.48.1). The kernel was set by the augment kcdf = ‘Gaussian’, based on our input expression being log_2_(TPM + 0.001).

### Cell deconvolution and clustering

We predicted relative TME cell proportions in the three trials based on bulk RNA-seq by using Kassandra^54^, which showed better single-cell-level accuracy and stability than previous tools. We performed 1000 times-repeated unsupervised hierarchical agglomerative clustering to identify cell patterns using the R package ConsensusClusterPlus (version 1.64.0) (reps = 1000, pItem = 0.8, pFeature = 1). Consensus cumulative distribution function plot testing the cluster number from 2 to 6 to determine the optimal number of clusters (maxK = 6). To enhance efficiency, other quicker quantification methods including MCP-counter^67^ and ESTIMATE^68^ were used for TCGA Pan-Cancer cohort.

### Evaluation of CDKN1C expression at single-cell level

We utilized 10 publicly available single-cell RNA-seq datasets across various cancer types collected by Ru et al. ^56^, including NSCLC, head and neck cancer, melanoma, colorectal cancer, breast cancer, and liver cancer. Cells were clustered into different lineages and sub-lineages based on Ru et al.‘s pipeline^56^, and CDKN1C expression was evaluated in each subpopulation. Additionally, we assessed CDKN1C expression in the largest single-cell fibroblast database to date (https://gist-fgl.github.io/sc-caf-atlas/#)^57^. This database included gene expression information for seven subsets of 104,692 fibroblasts, including myofibroblasts, inflammatory cancer-associated fibroblasts (CAFs), adipo-genic CAFs, endothelial-to-mesenchymal transition CAFs, peripheral nerve-like CAFs, antigen-presenting CAFs, and normal fibroblasts.

### Statistical analysis

A random-effect model was used to pool the trial-level HRs and 95% CIs for each cancer type using the R package meta (version 6.5-0), separately in PD-L1+ and PD-L1- subgroups. Statistical heterogeneity between trials was quantified using the *I*^*2*^ value. The heterogeneity of efficacy between PD-L1+ and PD-L1- subgroups was measured by *P* for interaction (*P*_*i*_). In the analyses of individual-patient level data, categorical and continuous variables were compared by the χ2 test and Wilcoxon rank-sum test, respectively. Kaplan–Meier method was used to estimate median OS and PFS, and to draw survival curves. Survival between two groups was compared by HRs and 95% CIs using Cox regression model using the R packages survival (version 3.5-5) and survminer (version 0.4.9), and the significance was tested by a log-rank test. We used Fisher’s exact test to compare ORR between two groups, and the Clopper-Pearson method for 95% CIs. The forest-plots and the heatmaps were generated using the R packages forestplot (version 3.1.1) and pheatmap (version 1.0.12), respectively. All analyses were conducted using R (version 4.2.1) and were considered statistically significant if two-sided *P* < 0.05.

### Reporting summary

Further information on research design is available in the Nature Research Reporting Summary linked to this article.

### Supplementary information

Supplementary Materials

Reporting summary

## Data Availability

The clinical and RNA-seq data of OAK and POPLAR trials can be requested in European Genome-phenome Archive (Accession numbers EGAD00001008391, EGAD00001008390, EGAD00001008549, EGAD00001008548, EGAD00001008550, and EGAD00001006630). The clinical and RNA-seq data of IMvigor210 trial are accessible in R package IMvigor210CoreBiologies. The 28 clinical trial data were obtained from public literature. The TCGA Pan-Cancer cohort data were obtained from UCSC Xena browser and GDC PanImmune Data Portal, as noted above in the Methods section.

## Code availability

This paper does not report original code, except for the Cox proportional hazard interaction test between PD-L1 expression and candidate variables. This part of the code is deposited at https://github.com/NPTL1201/PDL1_predictiveness_interaction/blob/main/code_R. Any additional information required to reanalyze the data reported in this paper is available from the lead contact upon reasonable request.
