# Supplementary material for: Deciphering transcriptomic determinants of the divergent link between PD-L1 and immunotherapy efficacy
Source: NPJ Precis Oncol. 2023 Sep 11;7:87. doi: 10.1038/s41698-023-00443-3 (PMC10495439; doi:10.1038/s41698-023-00443-3)
Supplement: Supplementary file 1 — Supplementary Materials [file 41698_2023_443_MOESM1_ESM.pdf]

Supplementary Materials

Supplementary Figure 1. Predictive value of PD-L1 expression for overall survival across cancer types.
Supplementary Figure 2. Predictive value of PD-L1 expression for progression-free survival across cancer types.
Supplementary Figure 3. Correlation between PD-L1 predictiveness for progression-free survival and that for overall survival across cancer types.
Supplementary Figure 4. Correlation between median levels of four previously established immunotherapy biomarkers and PD-L1 predictiveness across cancer types.
Supplementary Figure 5. Venn diagram presenting the genes significantly correlated with OS and PFS PD-L1 predictiveness across cancer types.
Supplementary Figure 6. Correlation between *CDKN1C* gene expression and PD-L1 predictiveness for overall survival across cancer types.
Supplementary Figure 7. Analysis of context-dependent PD-L1 predictiveness by three PD-L1 expression levels in OAK trial.
Supplementary Figure 8. Analysis of context-dependent PD-L1 predictiveness by three PD-L1 expression levels in POPLAR trial.
Supplementary Figure 9. Analysis of context-dependent PD-L1 predictiveness by three PD-L1 gene expression levels in IMvigor210 trial.
Supplementary Figure 10. Kaplan-Meier survival curve of atezolizumab versus docetaxel stratified by PD-L1 immunohistochemistry expression in Predictiveness-High patients from the OAK trial.
Supplementary Figure 11. Kaplan-Meier survival curve of atezolizumab versus docetaxel stratified by PD-L1 immunohistochemistry expression in Predictiveness-Low patients from the OAK trial.
Supplementary Figure 12. Kaplan-Meier survival curve of atezolizumab versus docetaxel stratified by PD-L1 gene expression in Predictiveness-High patients from the OAK trial.
Supplementary Figure 13. Kaplan-Meier survival curve of atezolizumab versus docetaxel stratified by PD-L1 gene expression in Predictiveness-Low patients from the OAK trial.
Supplementary Figure 14. Context-dependent capacity for PD-L1 expression to predict benefit of immune checkpoint inhibitor over chemotherapy in POPLAR trial.
Supplementary Figure 15. Kaplan-Meier survival curve of atezolizumab versus docetaxel stratified by PD-L1 immunohistochemistry expression in Predictiveness-High patients from the POPLAR trial.
Supplementary Figure 16. Kaplan-Meier survival curve of atezolizumab versus docetaxel stratified by PD-L1 immunohistochemistry expression in Predictiveness-Low patients from the POPLAR trial.
Supplementary Figure 17. Kaplan-Meier survival curve of atezolizumab versus docetaxel stratified by PD-L1 gene expression in Predictiveness-High patients from the POPLAR trial.
Supplementary Figure 18. Kaplan-Meier survival curve of atezolizumab versus docetaxel stratified by PD-L1 gene expression in Predictiveness-Low patients from the POPLAR trial.
Supplementary Figure 19. Association of PD-L1 and other inflammatory biomarkers.
Supplementary Figure 20. Correlation between PD-L1 and PD-1-high CD8+ T cells.
Supplementary Figure 21. Evaluation of PD-L1 upregulation mechanisms in Predictiveness-High and Predictiveness-Low patients across three trials.
Supplementary Figure 22. The landscape of the immune subtype in Predictiveness-High group.
Supplementary Figure 23. The landscape of the immune subtype in Predictiveness-Low group.
Supplementary Figure 24. Testing the robustness of our proposed PD-L1 predictiveness by immune subtype.
Supplementary Figure 25. Spearman correlations between PD-L1 and other immune checkpoints in Predictiveness-High and Predictiveness-Low patients across three trials.
Supplementary Figure 26. Kaplan-Meier overall survival curve with atezolizumab stratified by inflammatory biomarkers among Predictiveness-High and Predictiveness-Low patients.
Supplementary Figure 27. Kaplan-Meier overall survival curve with atezolizumab stratified by immune

subtype.
Supplementary Figure 28. Testing the robustness of our proposed PD-L1 predictiveness by tumor mutation burden.
Supplementary Figure 29. Testing the robustness of our proposed PD-L1 predictiveness by tumor neoantigen burden in IMvigor210 trial.
Supplementary Figure 30. Kaplan-Meier overall survival curve with atezolizumab stratified by tumor mutation burden in Predictiveness-High and Predictiveness-Low group.
Supplementary Figure 31. Kaplan-Meier overall survival curve with atezolizumab stratified by tumor neoantigen burden in Predictiveness-High and Predictiveness-Low group in IMvigor210 trial.
Supplementary Figure 32. The fraction of Kassandra-based cells in Predictiveness-High group versus Predictiveness-Low group.
Supplementary Figure 33. Association of PD-L1 predictiveness score with pathways related to stromal activity.
Supplementary Figure 34. Spearman correlation of PD-L1 predictiveness score with fibroblast, endothelium, or stromal score in 31 TCGA cancer types.
Supplementary Figure 35. Count distribution for patients in IMvigor210 trial.
Supplementary table 1. Main characteristics of the trials used for evaluation of cross-cancer PD-L1 predictive capacity.
Supplementary table 2. Hazard ratios of overall survival for anti-PD-1/PD-L1 versus standard treatment stratified by PD-L1 expression.
Supplementary table 3. Hazard ratios of progression-free survival for anti-PD-1/PD-L1 versus standard treatment stratified by PD-L1 expression.
Supplementary table 4. Evaluation of PD-L1 predictive capacity by target of immune checkpoint.
Supplementary table 5. Median values of four previously established immunotherapy biomarkers across a cancer type.
Supplementary table 6. Median values of 1058 immune-related genes across a cancer type.
Supplementary table 7. The median levels of 31 genes significantly correlated with PD-L1 predictiveness for overall survival and progression-free survival across cancer types.
Supplementary table 8. The interaction test between the PD-L1 and candidate genes using atezolizumab-treated patients from OAK trial.
Supplementary table 9. Baseline characteristics of the OAK trial according to PD-L1 gene expression within Predictiveness-High and Predictiveness-Low groups.
Supplementary table 10. Baseline characteristics of the POPLAR trial according to PD-L1 gene expression within Predictiveness-High and Predictiveness-Low groups.
Supplementary table 11. Baseline characteristics of the IMvigor210 trial according to PD-L1 gene expression within Predictiveness-High and Predictiveness-Low groups.
Supplementary table 12. The interaction test between the PD-L1 and CDKN1C using atezolizumab-treated patients from POPLAR and IMvigor210 trials.
Supplementary table 13. Distribution of immune subtype according to PD-L1 gene expression within Predictiveness-High and Predictiveness-Low groups.
Supplementary table 14. Gene signatures used in this study.
eReference. References for Supplementary table 1.

Supplementary Figure 1. Predictive value of PD-L1 expression for overall survival across cancer types.

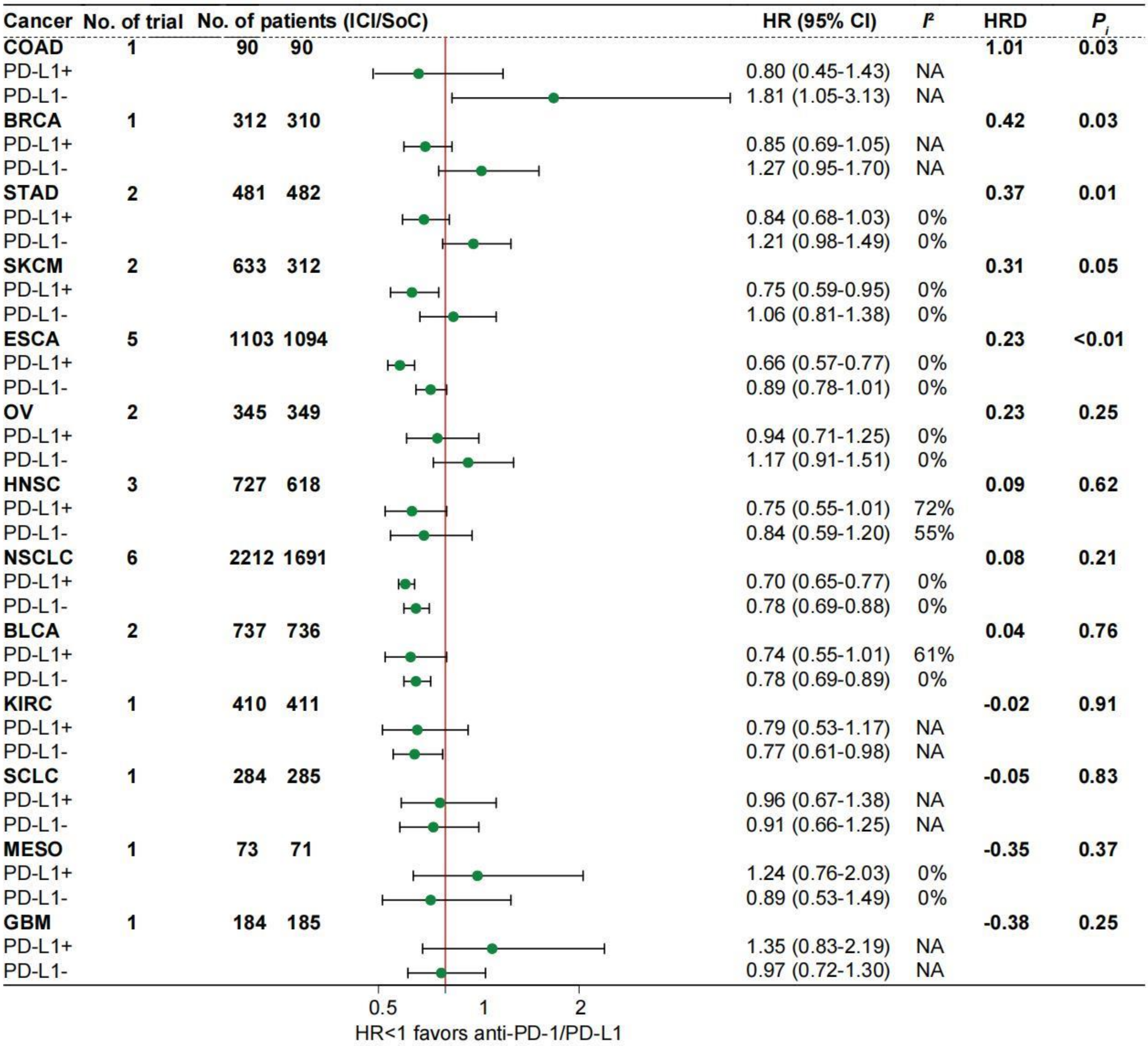

Pooled hazard ratio (HR) of overall survival (OS) between anti-PD-1/PD-L1 and standard-of-care for PD-L1+ and PD-L1- subgroups across cancer types. For a given cancer type, we defined PD-L1 predictive capacity as pooled HR of PD-L1- subgroup minus that of PD-L1+ subgroup (Hazard ratio difference, HRD). Cancer types were ranked based on their OS HRD. Error bars represent 95% CI. P_i denotes the P value for interaction. The one MESO trial provided results of two PD-L1 antibody clones, therefore an I^2 value was available to assess heterogeneity. There were a small proportion of patients with gastro-esophageal junction cancer in STAD trials. Trials of BLCA investigated urothelial cancer and primary tumor sites of most patients were bladder. HRD, hazard ratio difference; ICI, immune checkpoint inhibitor; SoC, standard-of-care; HR, hazard ratio; CI, confidence interval; NA, not available; COAD, colon cancer; BRCA, breast cancer; STAD, stomach cancer; SKCM, melanoma; ESCA, esophageal cancer; OV, ovarian cancer; HNSC, head and neck cancer; NSCLC, non-small-cell lung cancer; BLCA, bladder cancer; KIRC, kidney clear cell carcinoma; SCLC, small-cell lung cancer; MESO, mesothelioma; GBM, glioblastoma.

Supplementary Figure 2. Predictive value of PD-L1 expression for progression-free survival across cancer types.

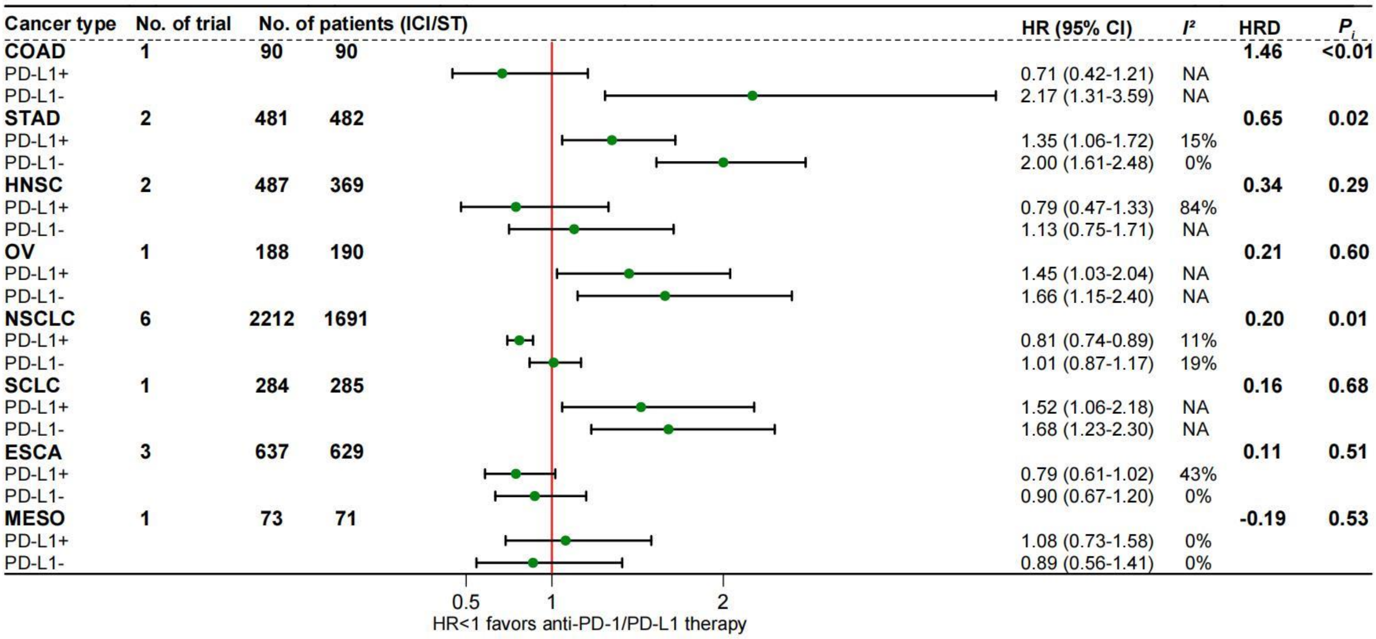

Pooled hazard ratio (HR) of progression-free survival (PFS) between anti-PD-1/PD-L1 and standard-of-care for PD-L1+ and PD-L1- subgroups across cancer types. For a given cancer type, we defined PD-L1 predictive capacity as pooled HR of PD-L1- subgroup minus that of PD-L1+ subgroup (Hazard ratio difference, HRD). Cancer types were ranked based on their PFS HRD. Error bars represent 95% CI. The one MESO trial provided results using two PD-L1 antibody clones, therefore an I^2 value was available to assess heterogeneity. There were a small proportion of patients with gastro-esophageal junction cancer in STAD trials. HRD, hazard ratio difference; ICI, immune checkpoint inhibitor; SoC, standard-of-care; HR, hazard ratio; CI, confidence interval; NA, not available; COAD, colon cancer; STAD, stomach cancer; HNSC, head and neck cancer; OV, ovarian cancer; NSCLC, non-small-cell lung cancer; SCLC, small-cell lung cancer; ESCA, esophageal cancer; MESO, mesothelioma. P_i denotes the P value for interaction.

Supplementary Figure 3. Correlation between PD-L1 predictiveness for progression-free survival and that for overall survival across cancer types.

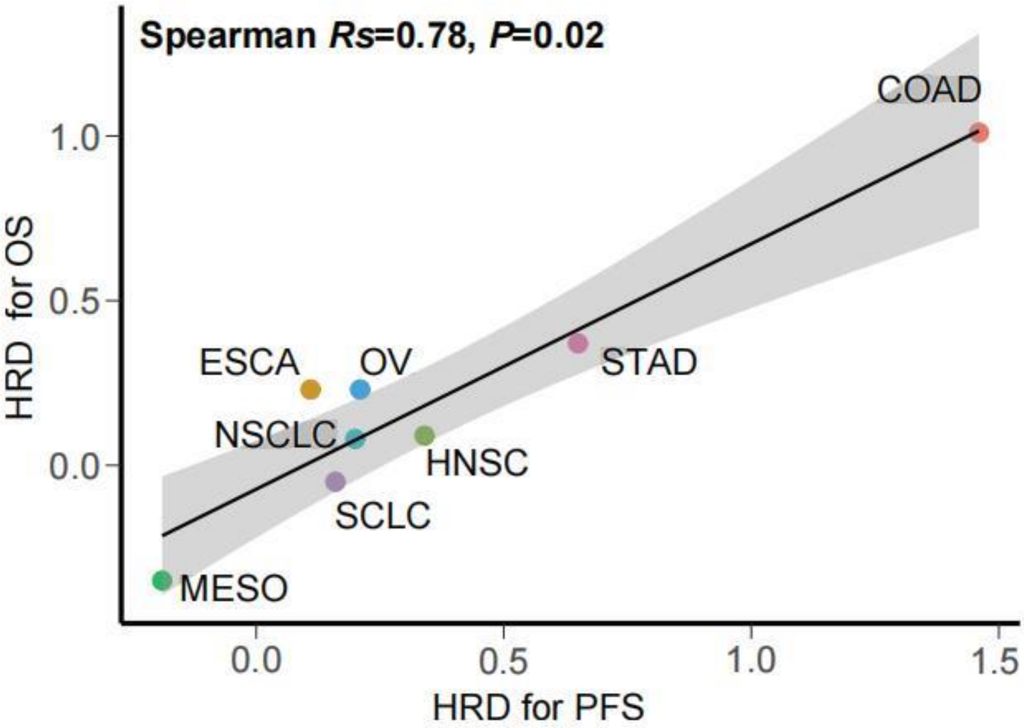

Rs denotes the spearman rank coefficient. *P* value indicates the significance test for spearman correlation. HRD, hazard ratio difference; OS, overall survival; PFS, progression-free survival; COAD, colon cancer; STAD, stomach cancer; ESCA, esophageal cancer; OV, ovarian cancer; HNSC, head and neck cancer; NSCLC, non-small-cell lung cancer; SCLC, small-cell lung cancer; MESO, mesothelioma.

Supplementary Figure 4. Correlation between median levels of four previously established immunotherapy biomarkers and PD-L1 predictiveness across cancer types.

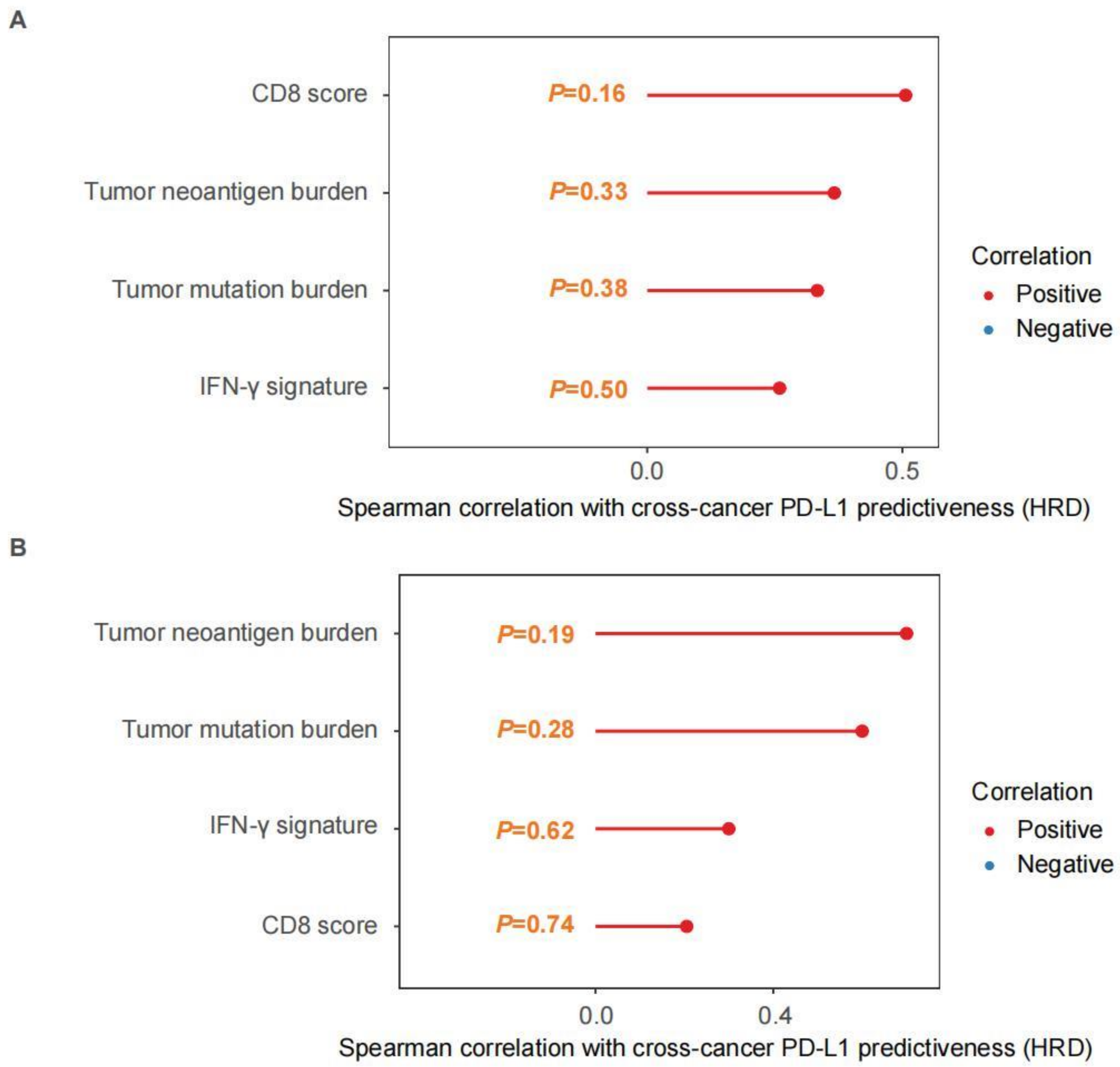

(A) overall survival. (B) progression-free survival. *P* value indicates the significance test for spearman correlation. HRD, hazard ratio difference; IFN, interferon; TCR; T-cell receptor.

Supplementary Figure 5. Venn diagram presenting the genes significantly correlated with OS and PFS PD-L1 predictiveness across cancer types.

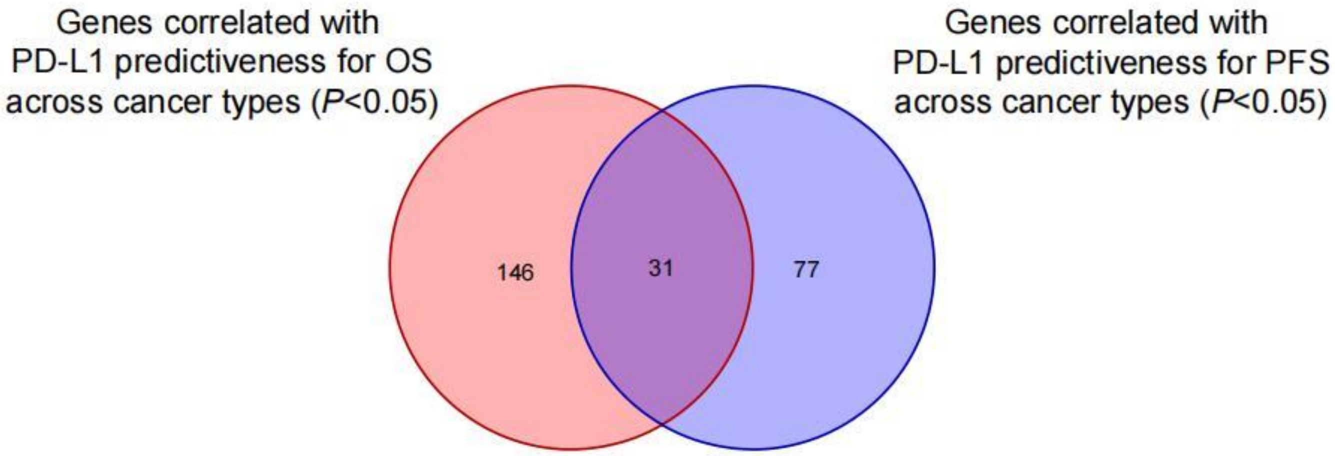

P value indicates the significance test for spearman correlation. OS, overall survival; PFS, progression-free survival.

Supplementary Figure 6. Correlation between *CDKN1C* gene expression and PD-L1 predictiveness for overall survival across cancer types.

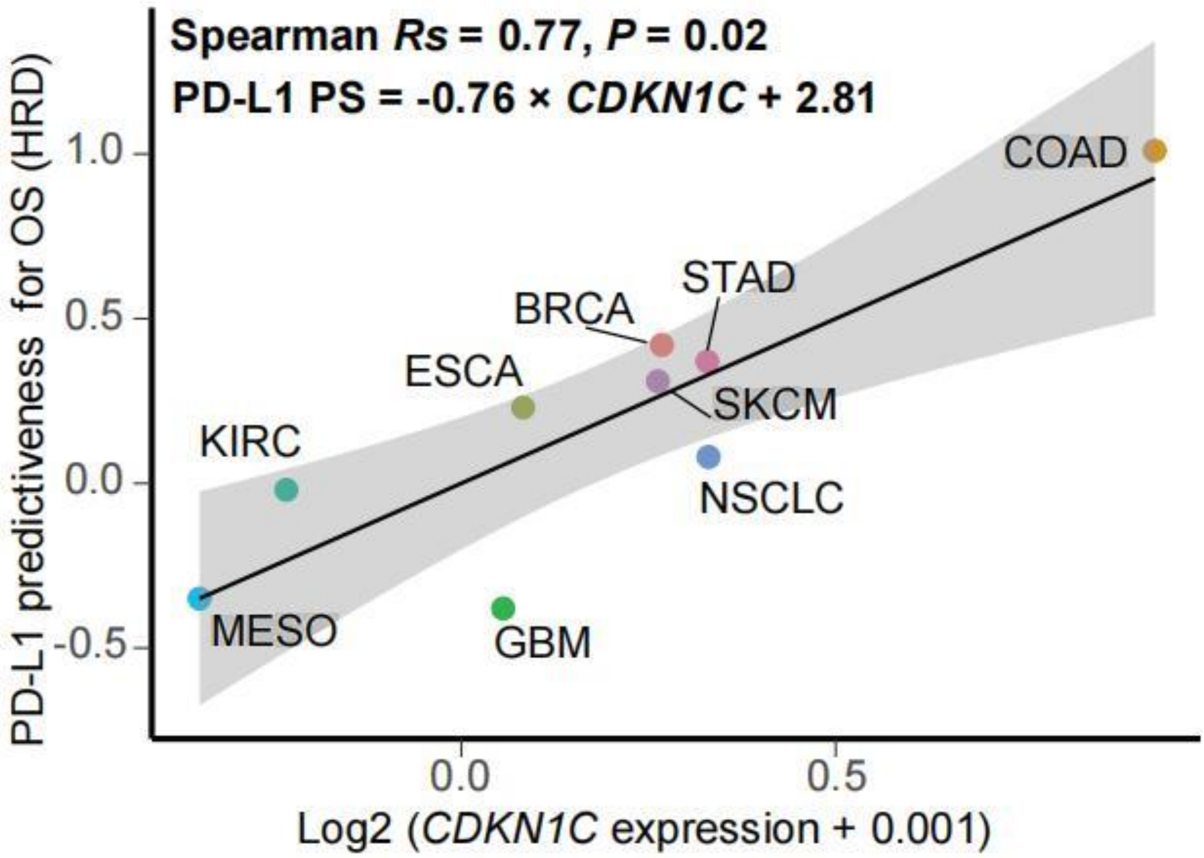

CDKN1C was identified as the strongest modulator of PD-L1 predictiveness and was used to develop a PD-L1 predictiveness score based on linear regression between *CDKN1C* and PD-L1 predictiveness for overall survival. *Rs* denotes the spearman rank coefficient. *P* value indicates the significance test for spearman correlation. HRD, hazard ratio difference; OS, overall survival; COAD, colon cancer; BRCA, breast cancer; STAD, stomach cancer; SKCM, melanoma; ESCA, esophageal cancer; NSCLC, non-small-cell lung cancer; KIRC, kidney clear cell carcinoma; MESO, mesothelioma; GBM, glioblastoma.

Supplementary Figure 7. Analysis of context-dependent PD-L1 predictiveness by three PD-L1 expression levels in OAK trial.

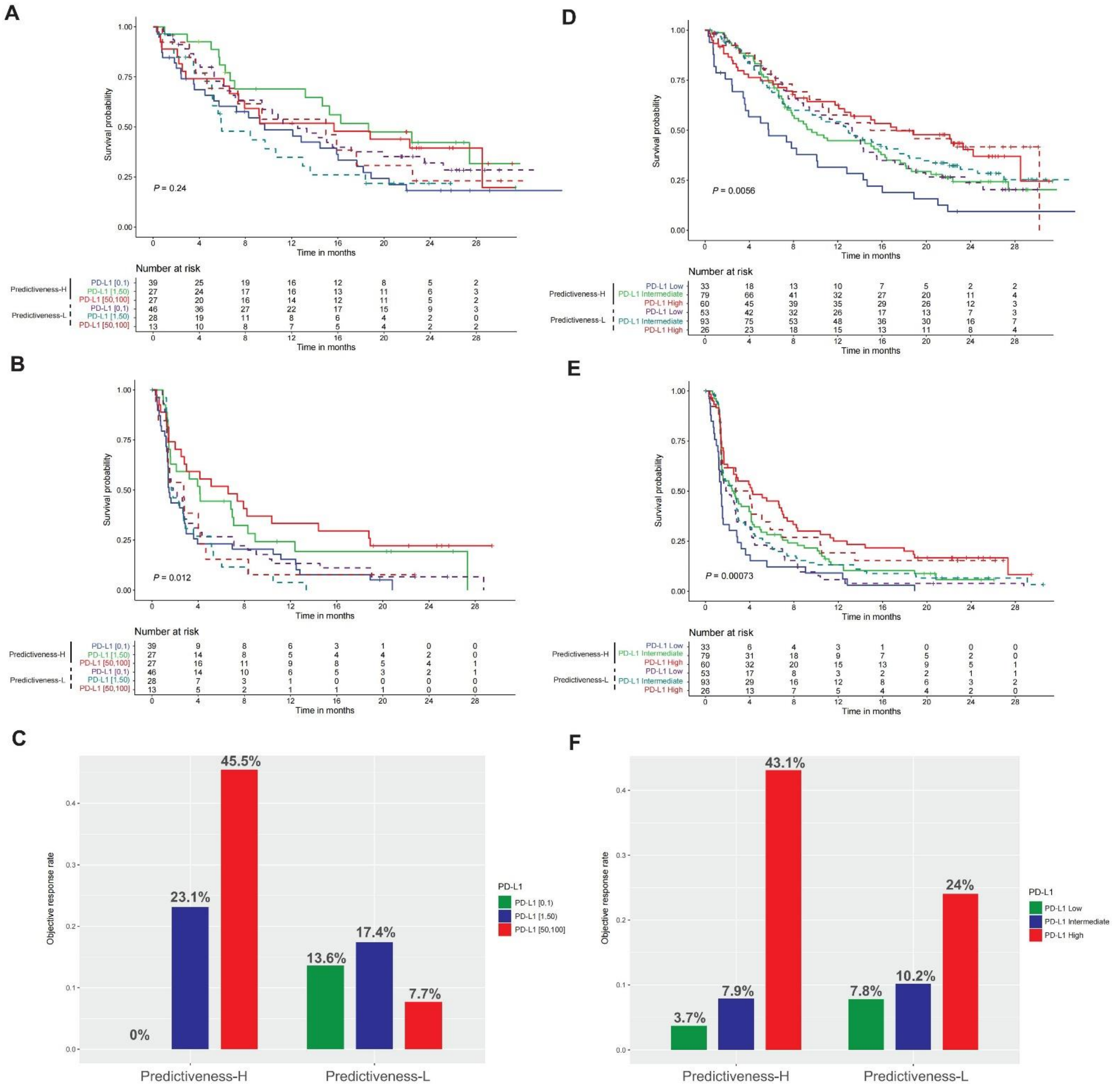

(A-C) Overall survival, progression-free survival, and objective response rate with atezolizumab stratified by PD-L1 immunohistochemistry expression based on cutoff values of 1% and 50% among Predictiveness-High and Predictiveness-Low patients. (D-F) The results of a similar analysis using PD-L1 gene expression. *P* value indicates log-rank test. The cutoff of PD-L1 predictiveness score was the median value of atezolizumab-treated patients, and the cutoffs of PD-L1 gene expression were the lower and upper quartiles of atezolizumab-treated patients.

Supplementary Figure 8. Analysis of context-dependent PD-L1 predictiveness by three PD-L1 expression levels in POPLAR trial.

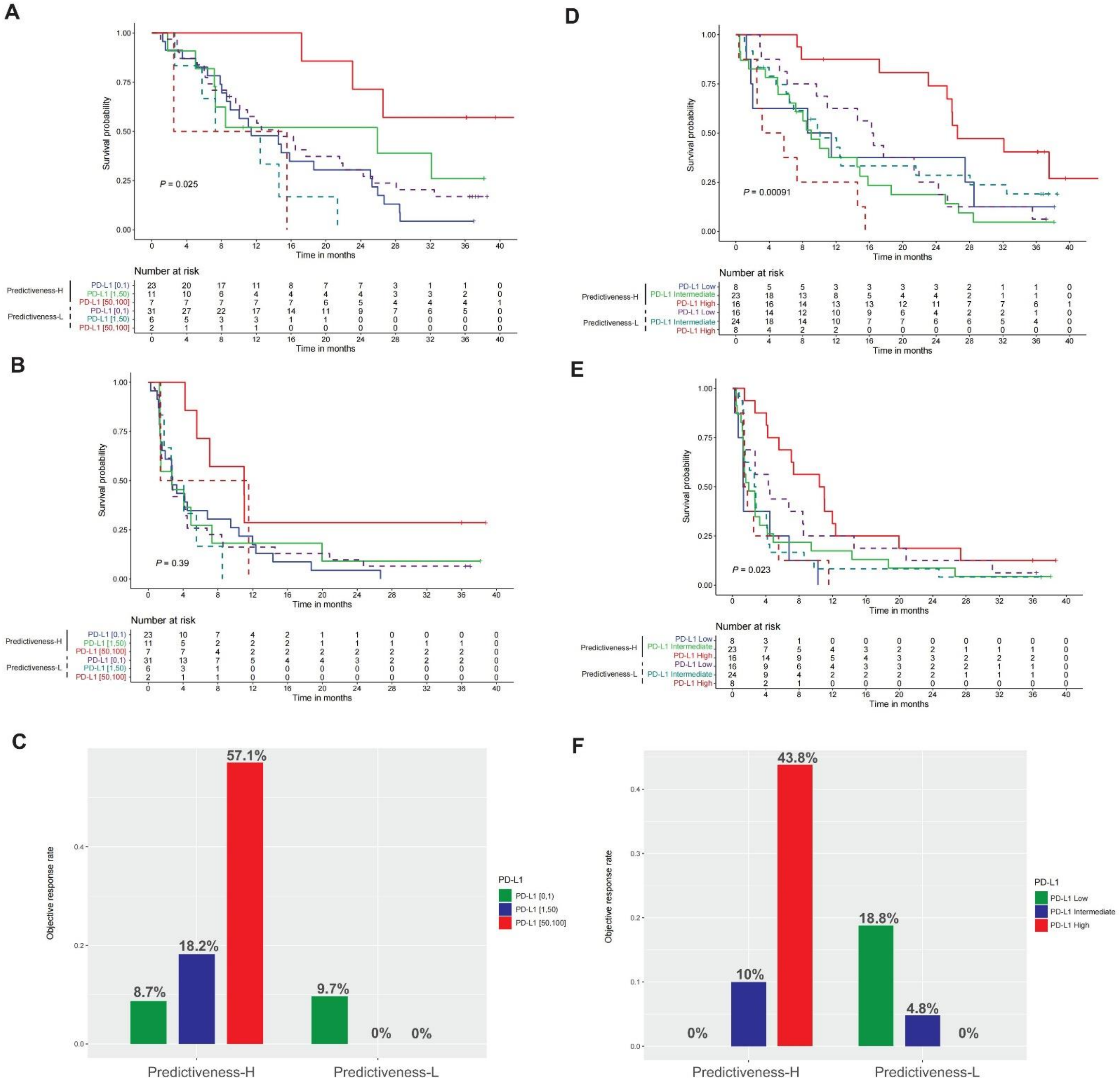

(A-C) Overall survival, progression-free survival, and objective response rate with atezolizumab stratified by PD-L1 immunohistochemistry expression based on cutoff values of 1% and 50% among Predictiveness-High and Predictiveness-Low patients. (D-F) The results of a similar analysis using PD-L1 gene expression. *P* value indicates log-rank test. The cutoff of PD-L1 predictiveness score was the median value of atezolizumab-treated patients, and the cutoffs of PD-L1 gene expression were the lower and upper quartiles of atezolizumab-treated patients.

Supplementary Figure 9. Analysis of context-dependent PD-L1 predictiveness by three PD-L1 gene expression levels in IMvigor210 trial.

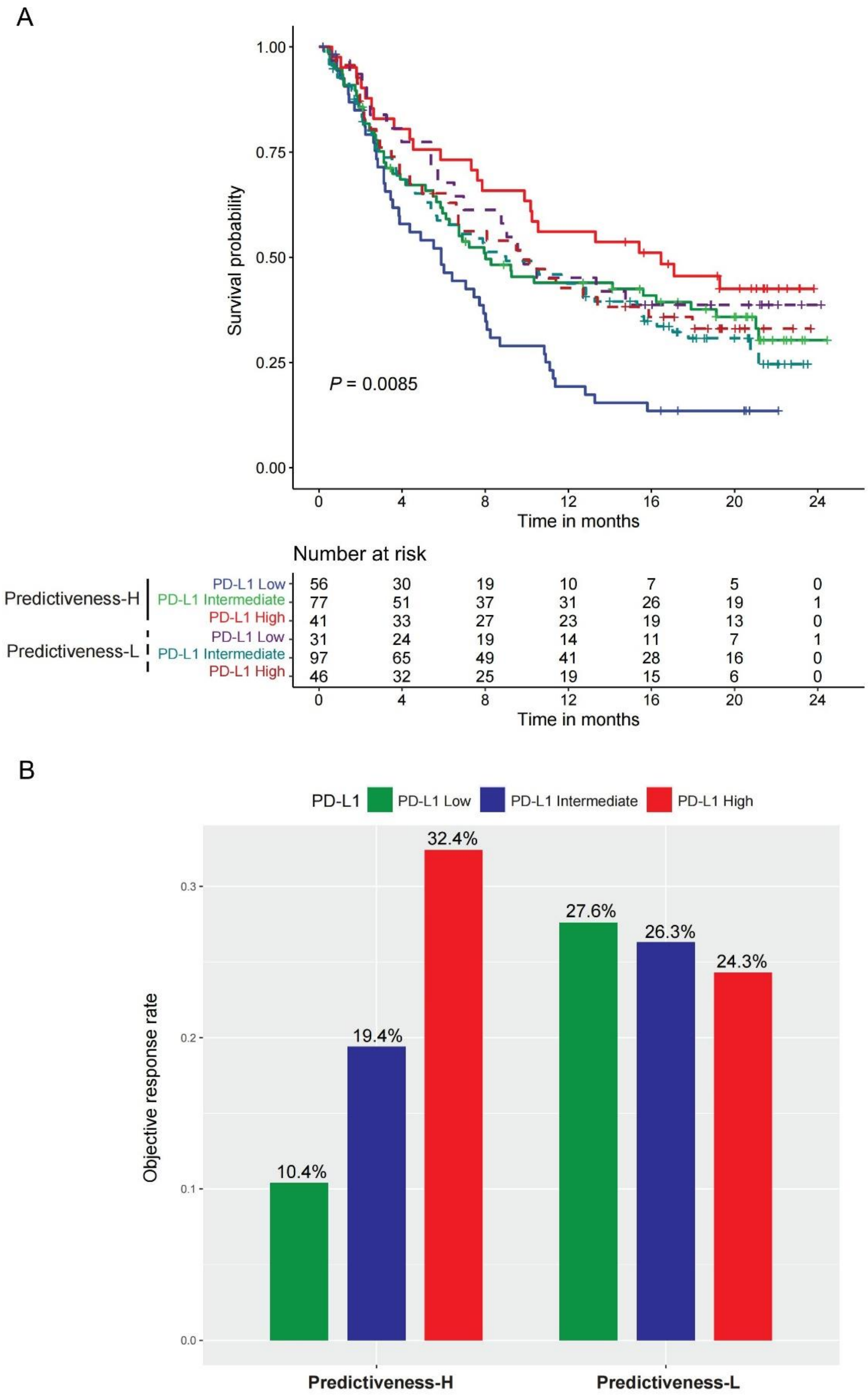

(A-B) Overall survival and objective response rate with atezolizumab stratified by PD-L1 gene expression among Predictiveness-High and Predictiveness-Low patients. *P* value indicates log-rank test. The cutoff of PD-L1 predictiveness score was the median value of atezolizumab-treated patients, and the cutoffs of PD-L1 gene expression were the lower and upper quartiles of atezolizumab-treated patients.

Supplementary Figure 10. Kaplan-Meier survival curve of atezolizumab versus docetaxel stratified by PD-L1 immunohistochemistry expression in Predictiveness-High patients from the OAK trial.

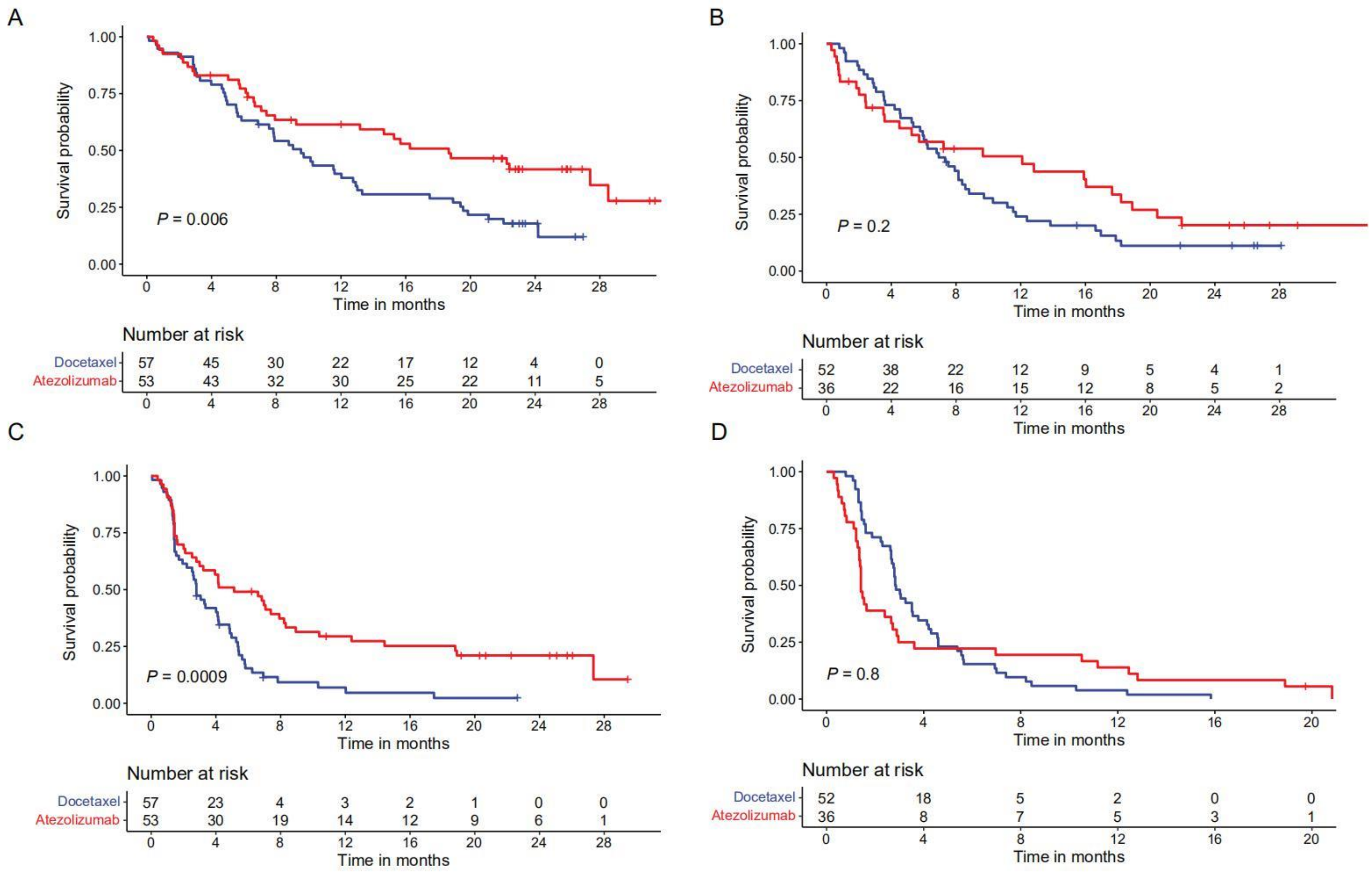

(A-B) Overall survival in patients with PD-L1≥1% and PD-L1<1% tumors, respectively. (C-D) Progression-free survival in patients with PD-L1≥1% and PD-L1<1% tumors, respectively. *P* value indicates log-rank test. The cutoff of PD-L1 predictiveness score was the median value of total intention-to-treat patients.

Supplementary Figure 11. Kaplan-Meier survival curve of atezolizumab versus docetaxel stratified by PD-L1 immunohistochemistry expression in Predictiveness-Low patients from the OAK trial.

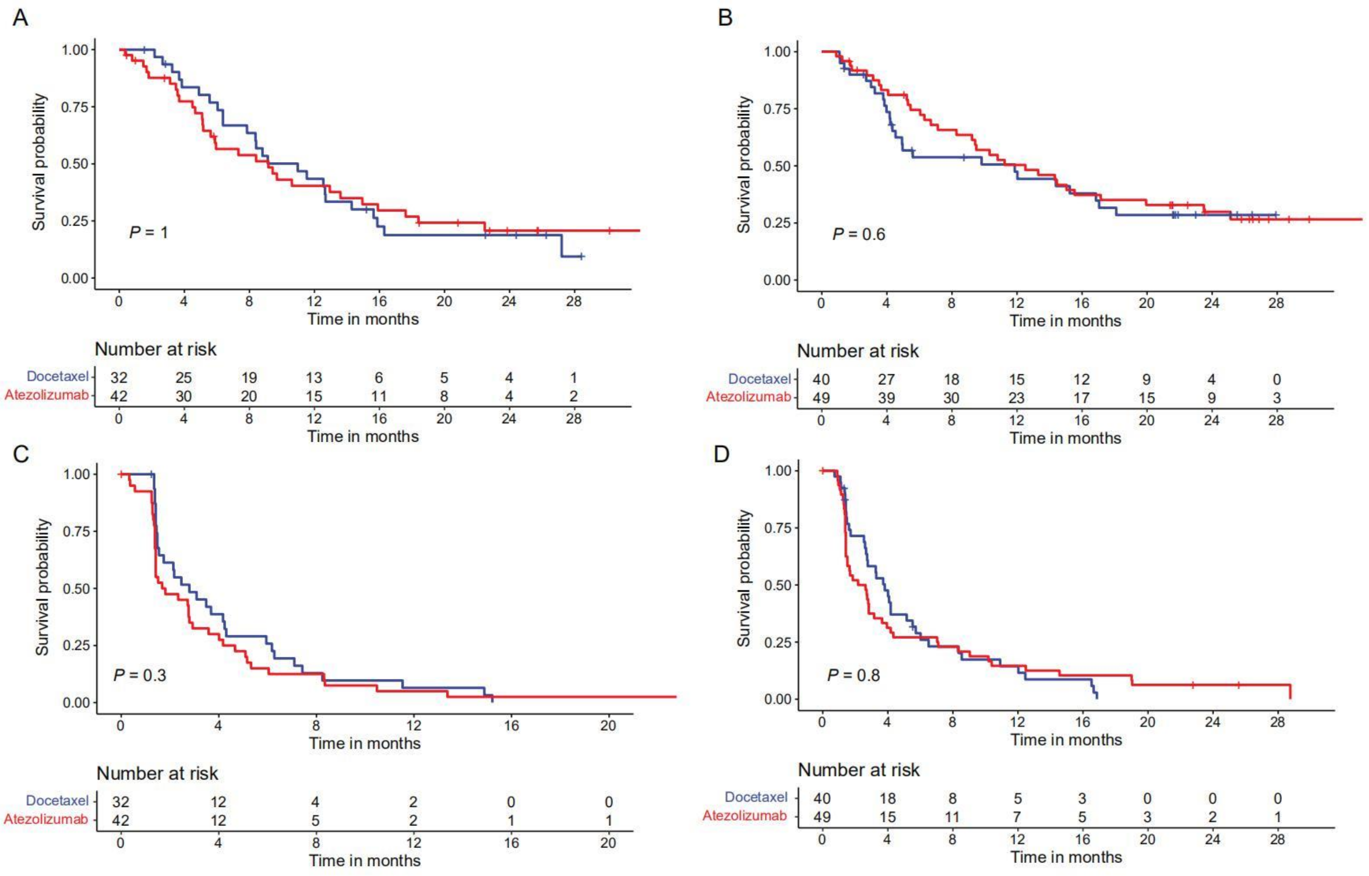

(A-B) Overall survival in patients with PD-L1≥1% and PD-L1<1% tumors, respectively. (C-D) Progression-free survival in patients with PD-L1≥1% and PD-L1<1% tumors, respectively. *P* value indicates log-rank test. The cutoff of PD-L1 predictiveness score was the median value of total intention-to-treat patients.

Supplementary Figure 12. Kaplan-Meier survival curve of atezolizumab versus docetaxel stratified by PD-L1 gene expression in Predictiveness-High patients from the OAK trial.

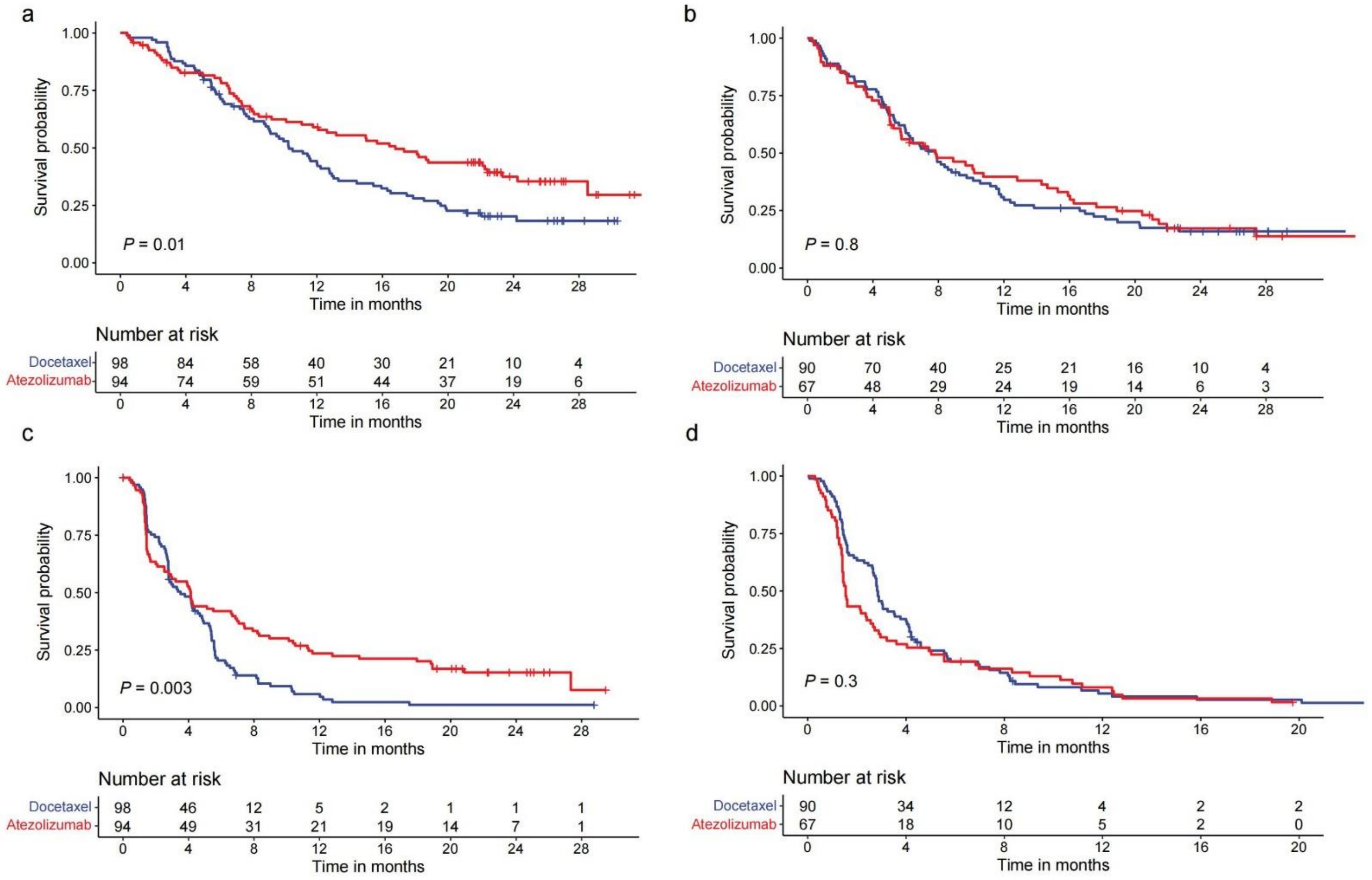

(A-B) Overall survival in patients with high and low PD-L1 expression, respectively. (C-D) Progression-free survival in patients with high and low PD-L1 expression, respectively. *P* value indicates log-rank test. The cutoffs of PD-L1 predictiveness score and PD-L1 gene expression were their median values of total intention-to-treat patients.

Supplementary Figure 13. Kaplan-Meier survival curve of atezolizumab versus docetaxel stratified by PD-L1 gene expression in Predictiveness-Low patients from the OAK trial.

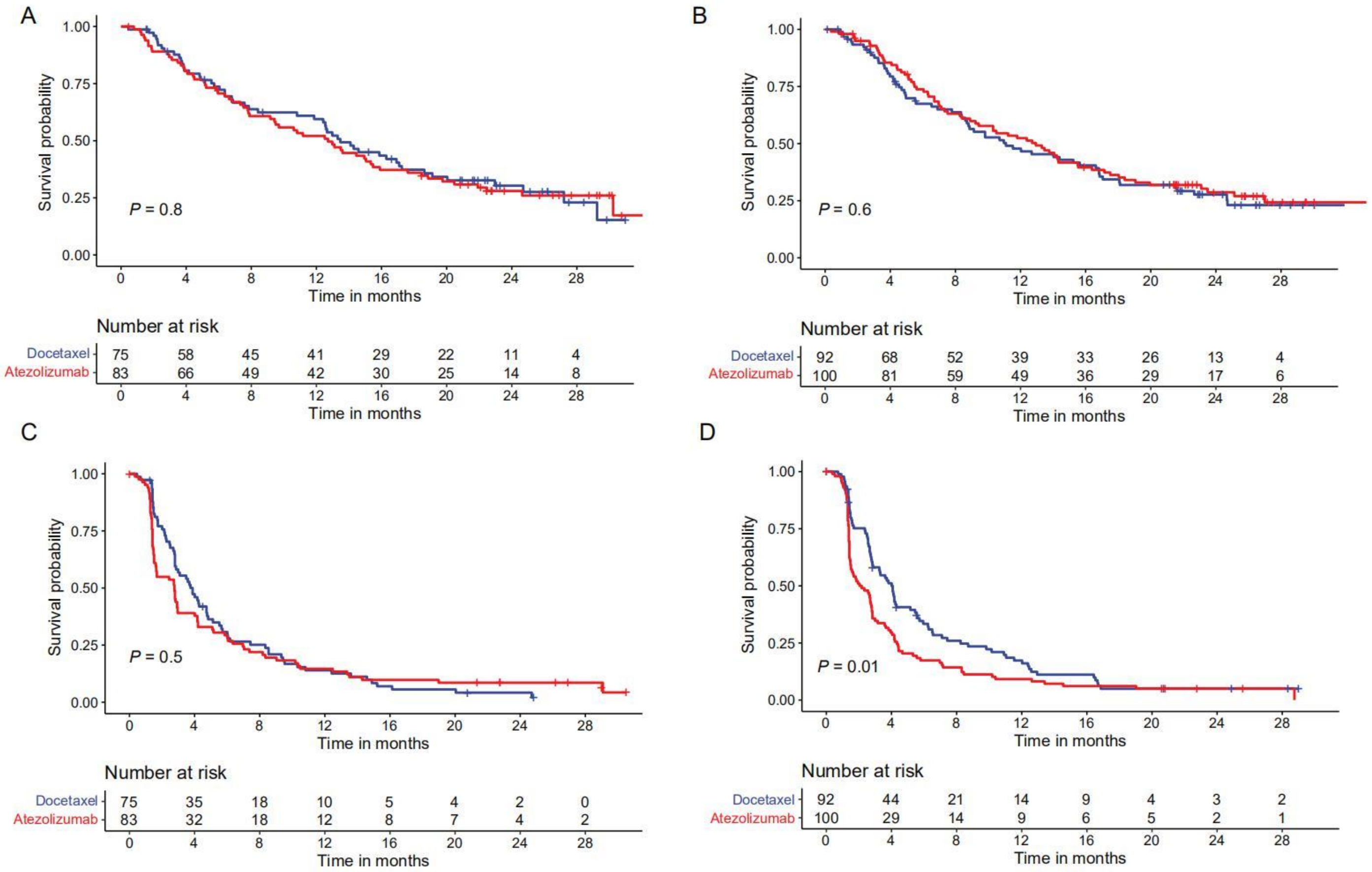

(A-B) Overall survival in patients with high and low PD-L1 expression, respectively. (C-D) Progression-free survival in patients with high and low PD-L1 expression, respectively. *P* value indicates log-rank test. The cutoffs of PD-L1 predictiveness score and PD-L1 gene expression were their median values of total intention-to-treat patients.

Supplementary Figure 14. Context-dependent capacity for PD-L1 expression to predict benefit of immune checkpoint inhibitor over chemotherapy in POPLAR trial.

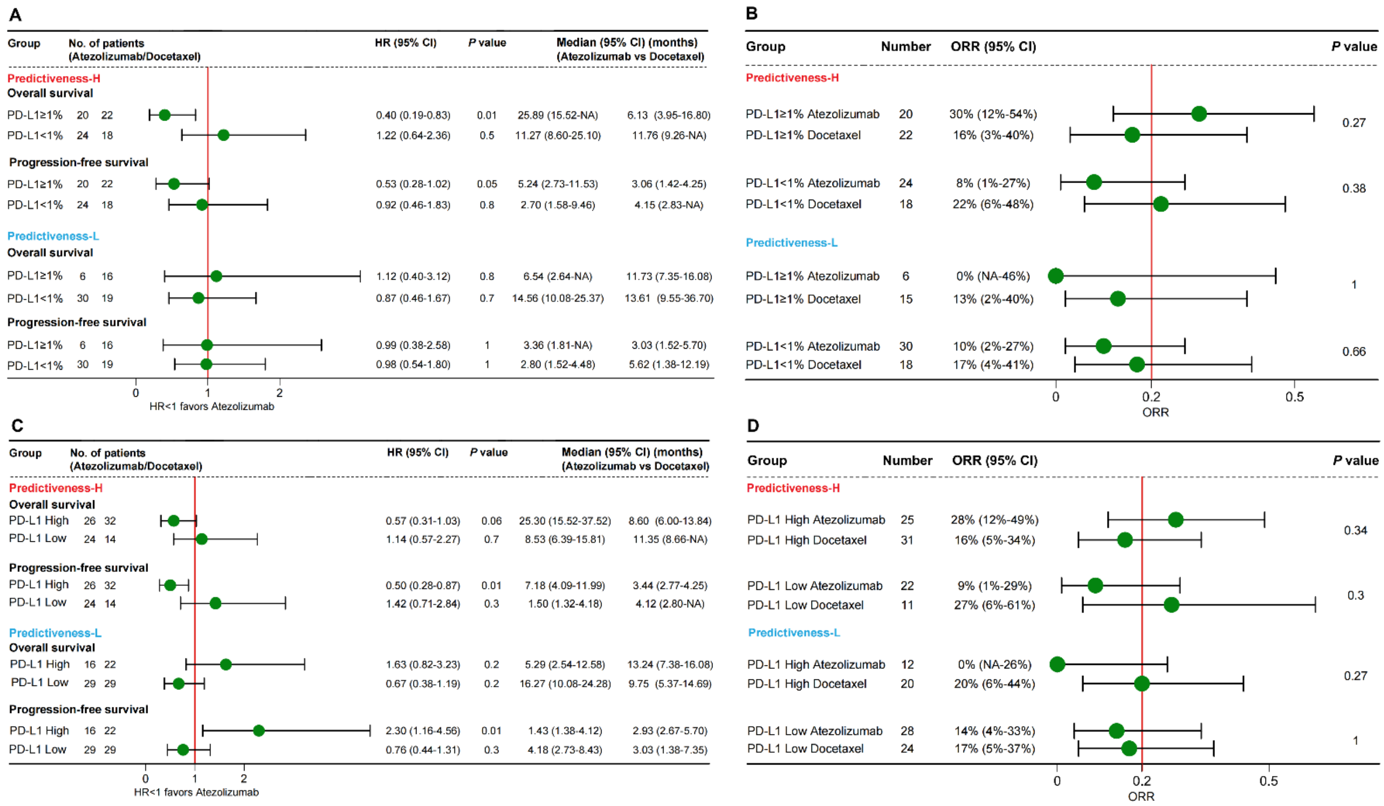

(A-B) Overall survival, progression-free survival, and objective response rate with atezolizumab versus docetaxel stratified by PD-L1 immunohistochemistry expression based on a cutoff of 1% among Predictiveness-High and Predictiveness-Low patients. (C-D) The results of a similar analysis using PD-L1 gene expression. Error bars represent 95% CI. *P* values for Panels A and C indicate log-rank test. *P* values for Panels B and D indicate Fisher's exact test. The cutoffs of PD-L1 predictiveness score and PD-L1 gene expression were their median values of total intention-to-treat patients. HR, hazard ratio; CI, confidence interval; ORR, objective response rate; NA, not available.

Supplementary Figure 15. Kaplan-Meier survival curve of atezolizumab versus docetaxel stratified by PD-L1 immunohistochemistry expression in Predictiveness-High patients from the POPLAR trial.

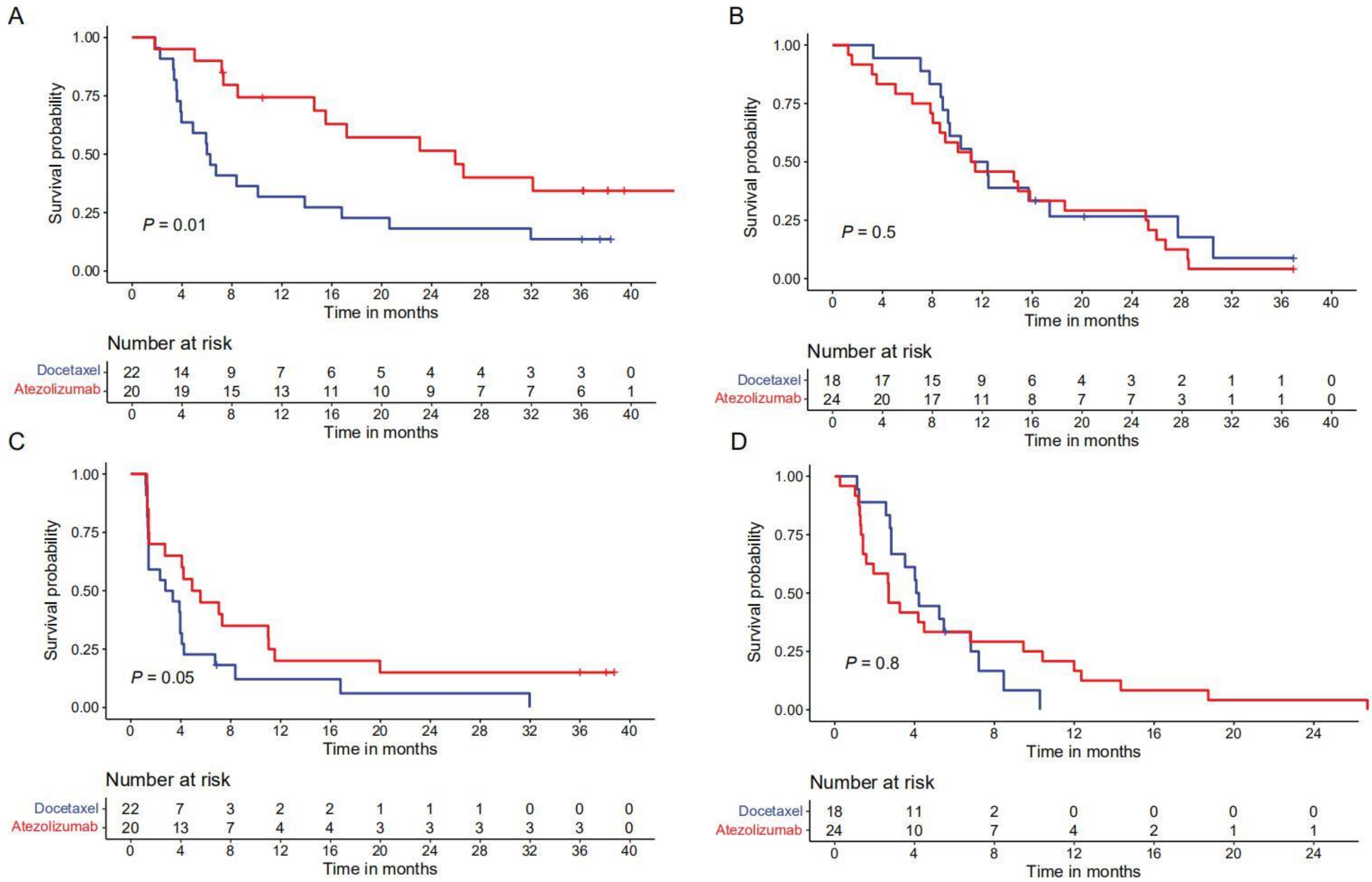

(A-B) Overall survival in patients with PD-L1≥1% and PD-L1<1% tumors, respectively. (C-D) Progression-free survival in patients with PD-L1≥1% and PD-L1<1% tumors, respectively. *P* value indicates log-rank test. The cutoff of PD-L1 predictiveness score was the median value of total intention-to-treat patients.

Supplementary Figure 16. Kaplan-Meier survival curve of atezolizumab versus docetaxel stratified by PD-L1 immunohistochemistry expression in Predictiveness-Low patients from the POPLAR trial.

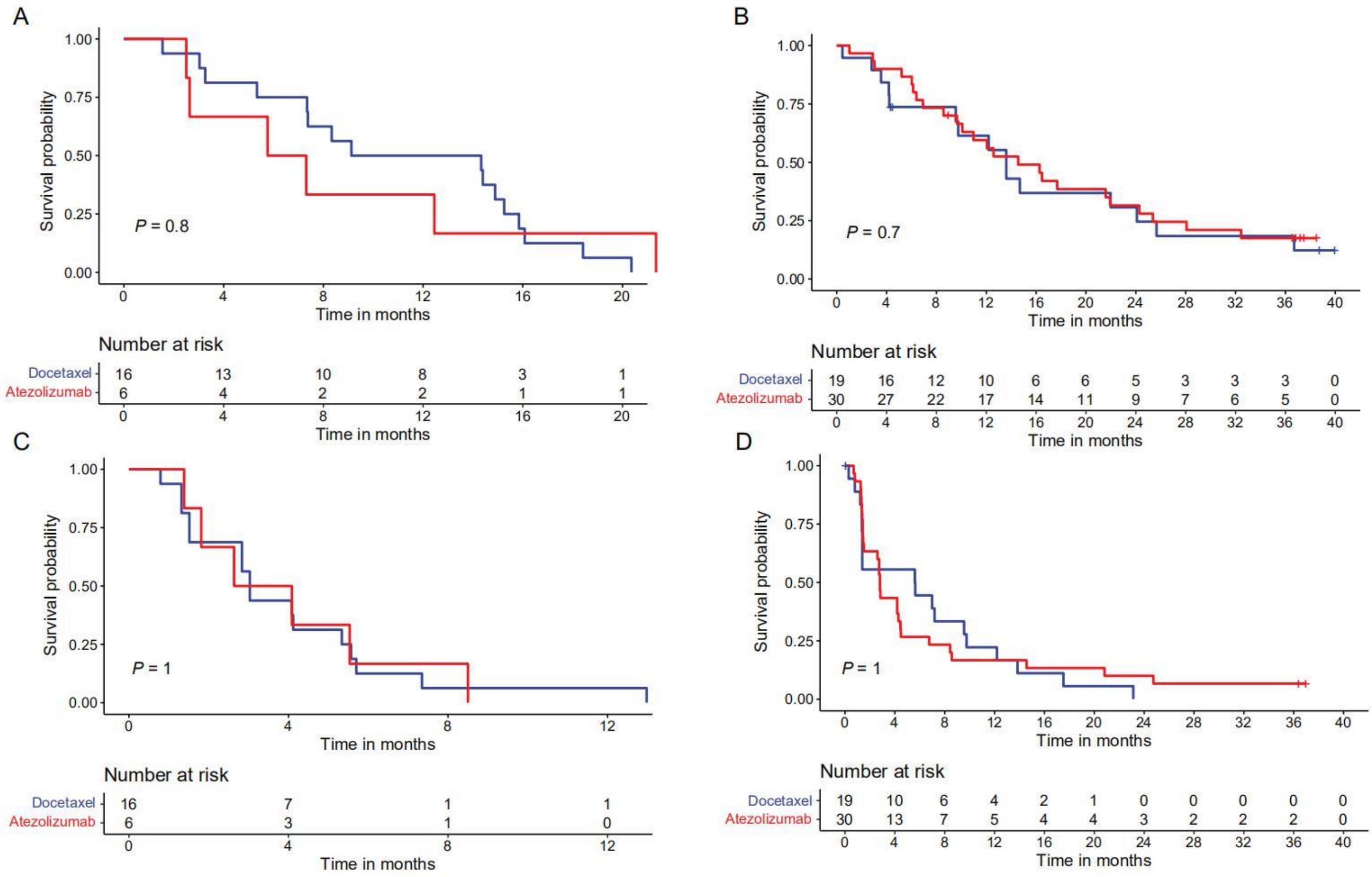

(A-B) Overall survival in patients with PD-L1≥1% and PD-L1<1% tumors, respectively. (C-D) Progression-free survival in patients with PD-L1≥1% and PD-L1<1% tumors, respectively. *P* value indicates log-rank test. The cutoff of PD-L1 predictiveness score was the median value of total intention-to-treat patients.

Supplementary Figure 17. Kaplan-Meier survival curve of atezolizumab versus docetaxel stratified by PD-L1 gene expression in Predictiveness-High patients from the POPLAR trial.

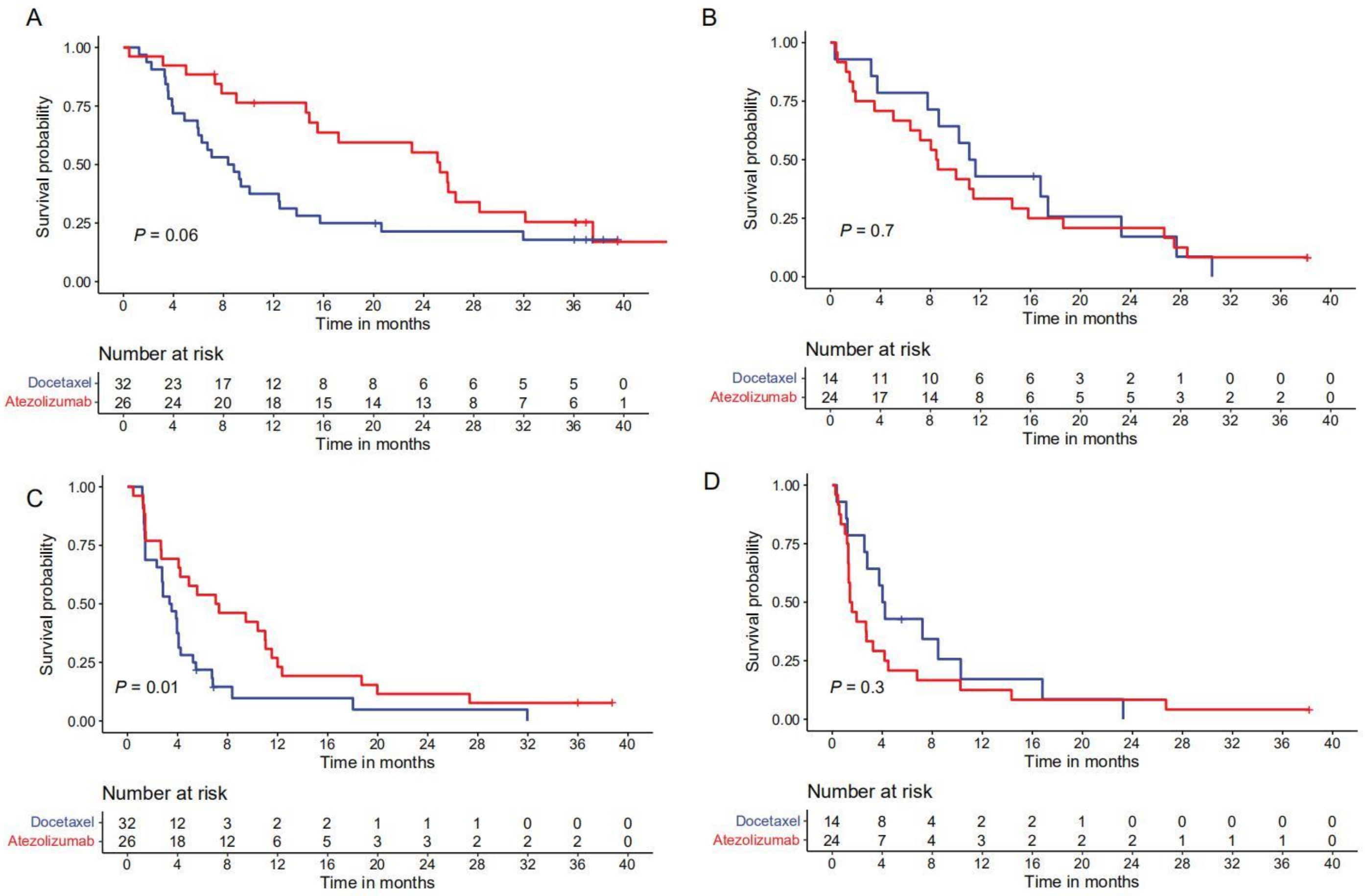

(A-B) Overall survival in patients with high and low PD-L1 expression, respectively. (C-D) Progression-free survival in patients with high and low PD-L1 expression, respectively. *P* value indicates log-rank test. The cutoffs of PD-L1 predictiveness score and PD-L1 gene expression were their median values of total intention-to-treat patients.

Supplementary Figure 18. Kaplan-Meier survival curve of atezolizumab versus docetaxel stratified by PD-L1 gene expression in Predictiveness-Low patients from the POPLAR trial.

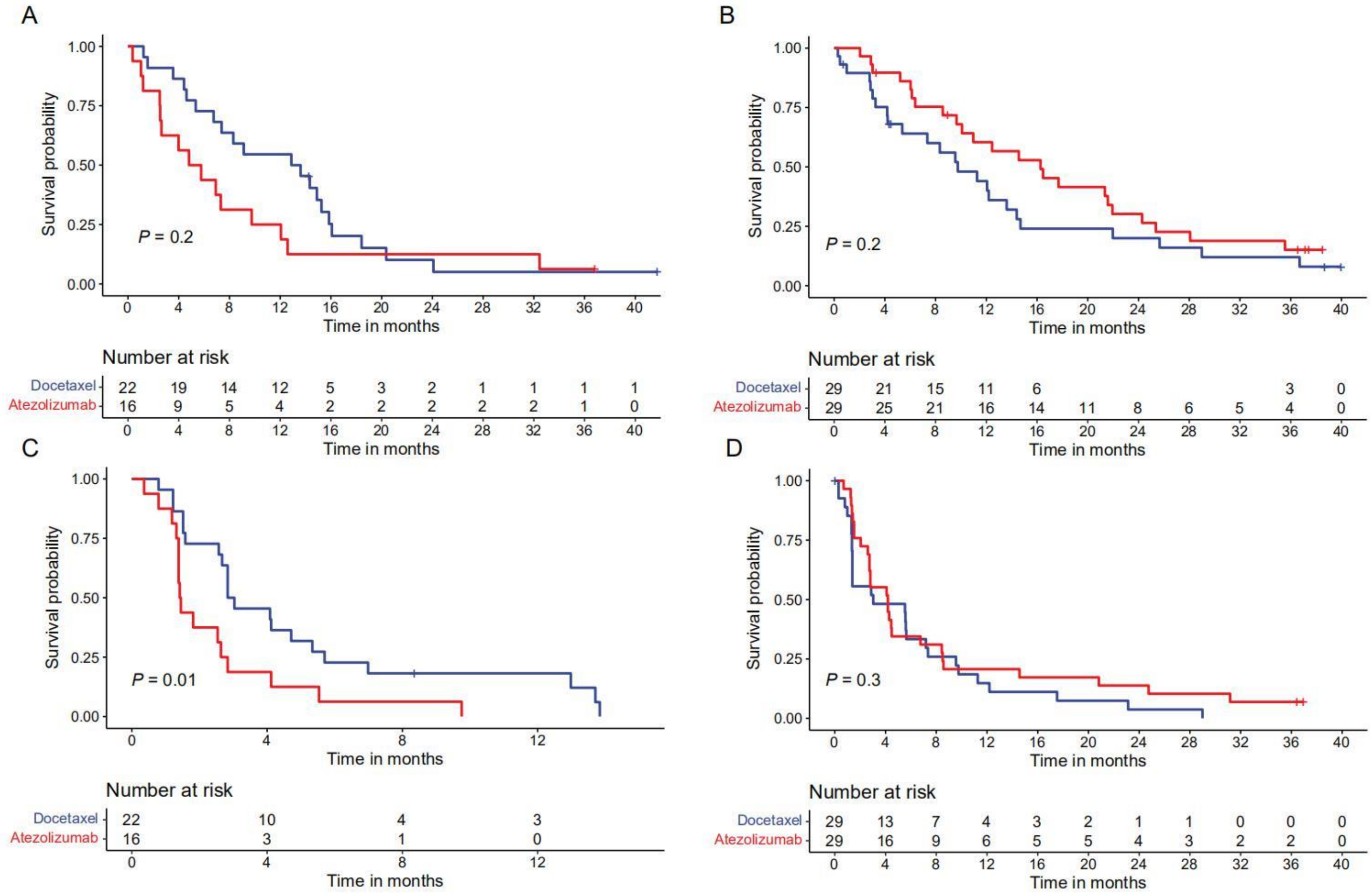

(A-B) Overall survival in patients with high and low PD-L1 expression, respectively. (C-D) Progression-free survival in patients with high and low PD-L1 expression, respectively. *P* value indicates log-rank test. The cutoffs of PD-L1 predictiveness score and PD-L1 gene expression were their median values of total intention-to-treat patients.

Supplementary Figure 19. Association of PD-L1 and other inflammatory biomarkers.

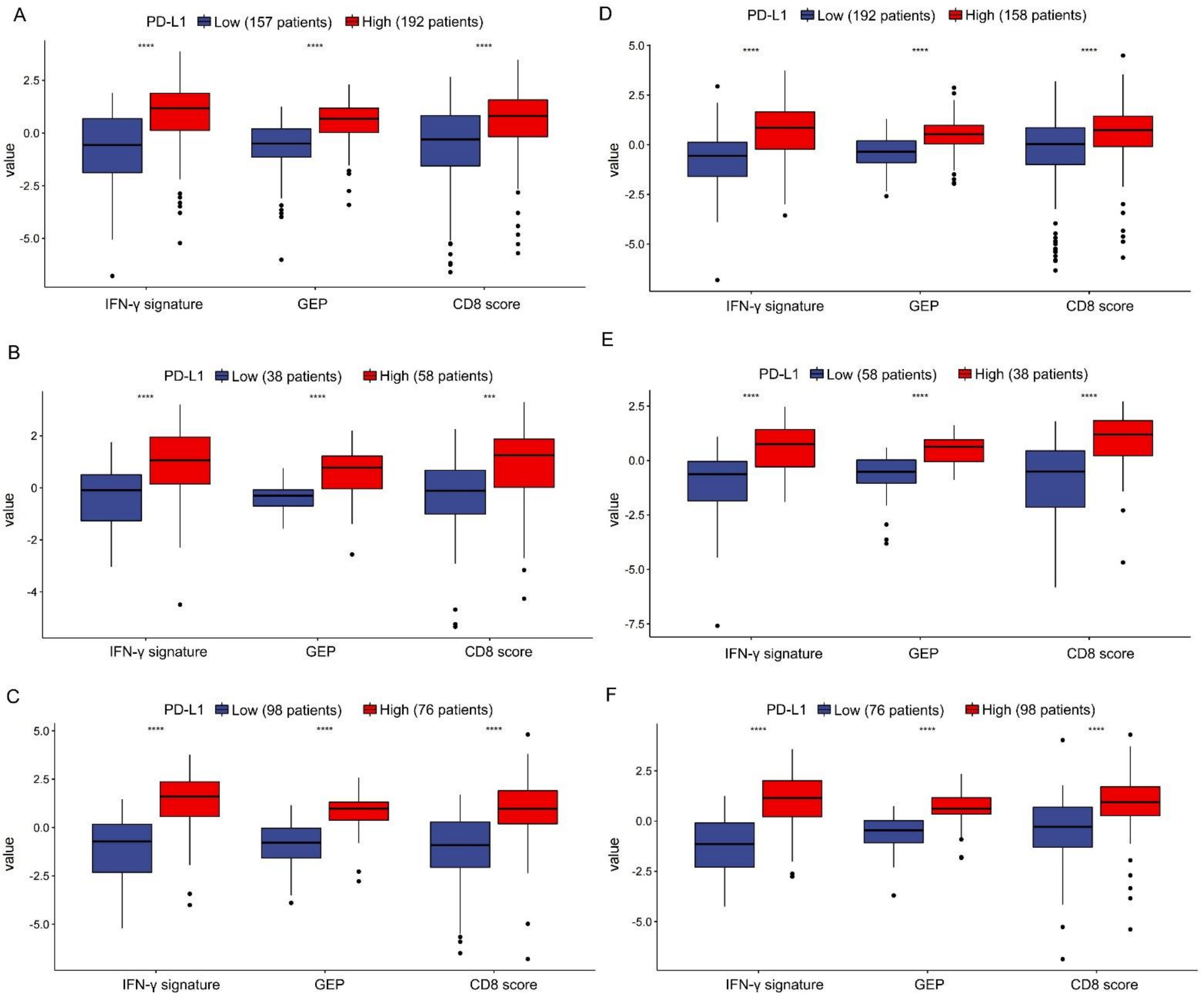

(A-C) Analysis of Predictiveness-High group in OAK, POPLAR, and IMvigor210 trials, respectively. (D-F) The results of a similar analysis in Predictiveness-Low group. The cutoffs of PD-L1 predictiveness score and PD-L1 gene expression were their median values of total intention-to-treat patients in each trial. The horizontal line in the boxes represents the median value. The bottom and top of the boxes are the lower and upper quartiles. The whiskers encompass 1.5 times the interquartile range. *P* value indicates Wilcoxon rank-sum test. The range of P values is labeled with asterisks. *, P < 0.05; **, P < 0.01; ***, P < 0.001; ****, P < 0.0001. IFN, interferon; GEP, T-cell inflamed gene expression profile.

Supplementary Figure 20. Correlation between PD-L1 and PD-1-high CD8+ T cells.

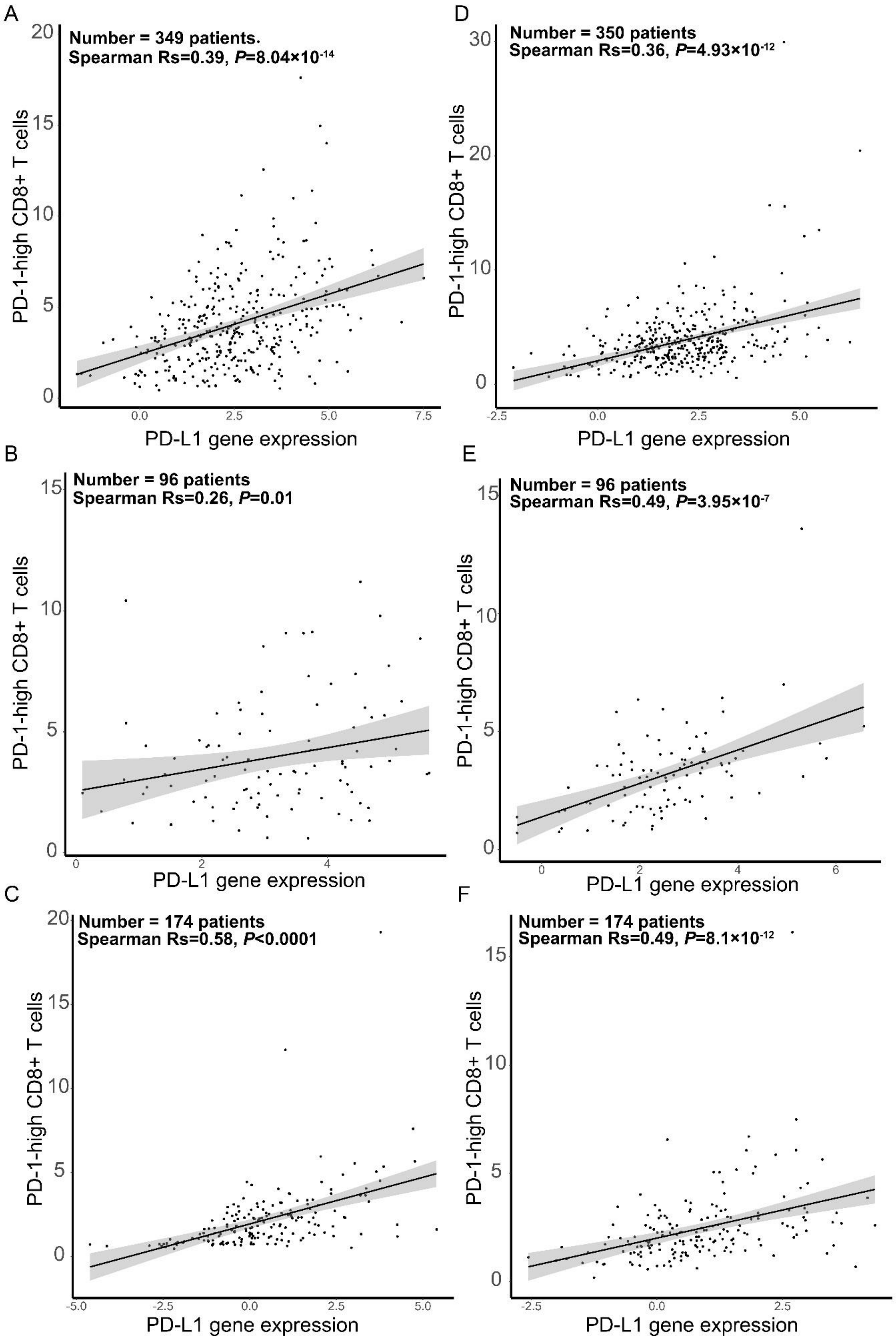

(A-C) Analysis of Predictiveness-High group in OAK, POPLAR, and IMvigor210 trials, respectively. (D-F) The results of a similar analysis in Predictiveness-Low group. The levels of PD-1-high CD8+ T cells were estimated using Kassandra algorithm. *P* value indicates the significance test for spearman correlation. *Rs* denotes the spearman rank coefficient.

Supplementary Figure 21. Evaluation of PD-L1 upregulation mechanisms in Predictiveness-High and Predictiveness-Low patients across three trials.

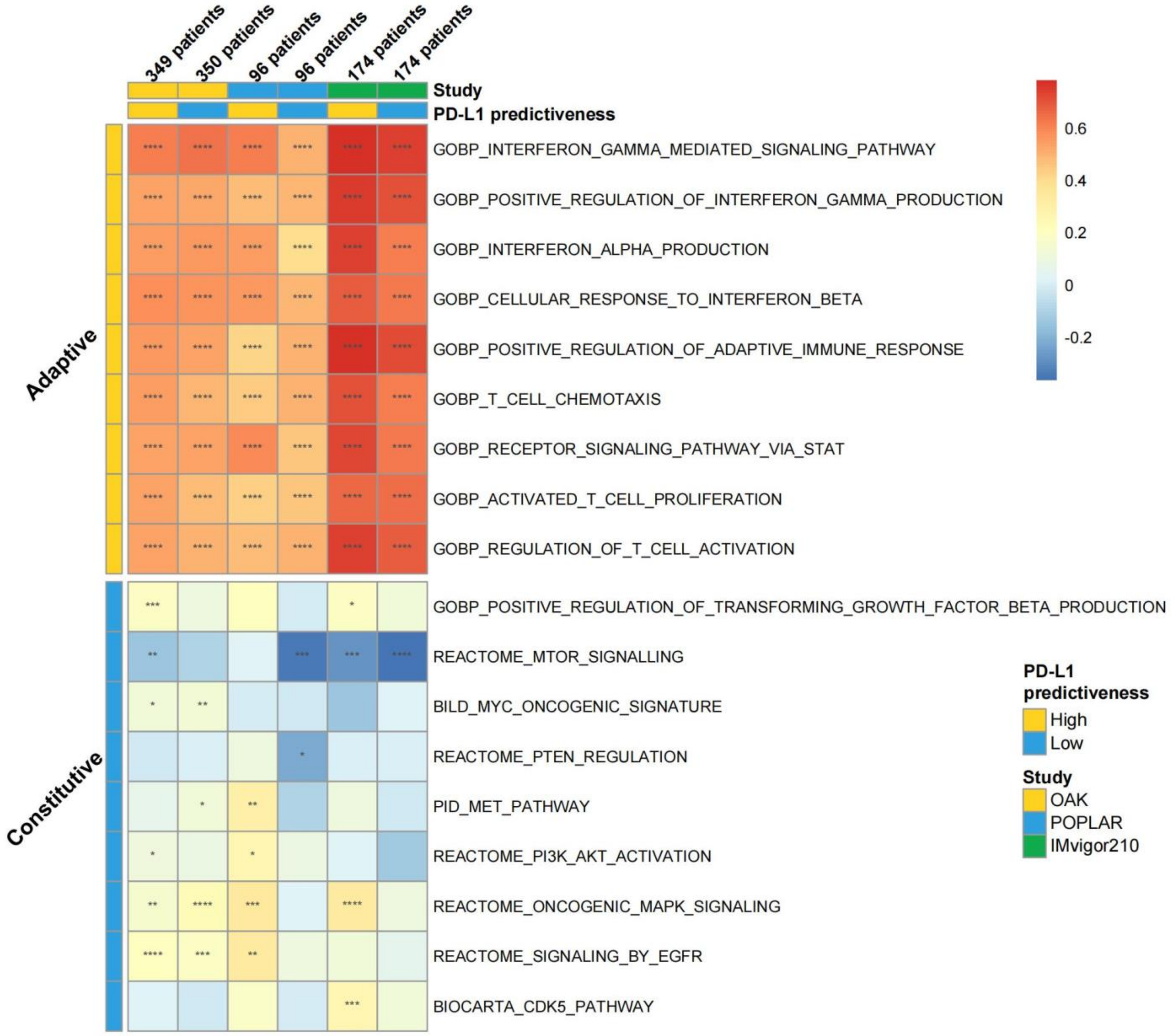

This graph depicts the spearman correlations between PD-L1 and enrichment scores of adaptive (upper panel) or constitutive (lower panel) pathways involved in PD-L1 upregulation. *P* value indicates the significance test for spearman correlation. The range of P values is labeled within each cell with asterisks. *, $P < 0.05$; **, $P < 0.01$; ***, $P < 0.001$; ****, $P < 0.0001$.

Supplementary Figure 22. The landscape of the immune subtype in Predictiveness-High group.

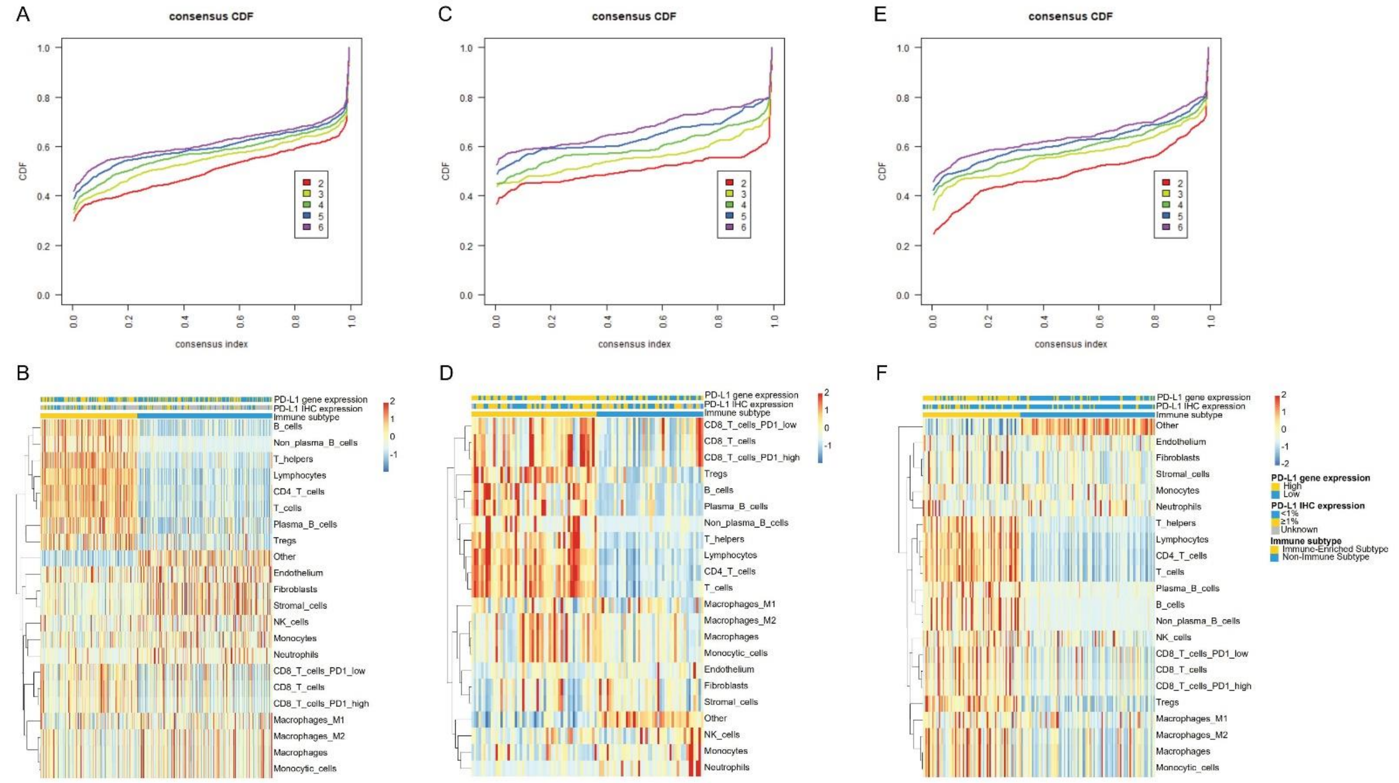

(A) Consensus cumulative distribution function plot testing the cluster number from 2 to 6 to determine the optimal number of clusters in OAK trial. The number of 2 was selected because it showed a relatively horizontal line and a sharper increase at 1. (B) Unsupervised clustering of Kassandra-based cells to classify patients from OAK trial into Immune-Enriched Subtype and Non-Immune Subtype. (C-D) The results of a similar analysis in POPLAR trial. (E-F) The results of a similar analysis in IMvigor210 trial. Data were represented as the z-score of population enrichment across each trial. The “Stromal cells” were calculated as a sum of "Endothelium" and "Fibroblasts" values. The ‘‘Other’’ indicated all cells not deconvolved by Kassandra, mainly including malignant cells and benign epithelial cells. CDF, cumulative distribution function; IHC, immunohistochemistry.

Supplementary Figure 23. The landscape of the immune subtype in Predictiveness-Low group.

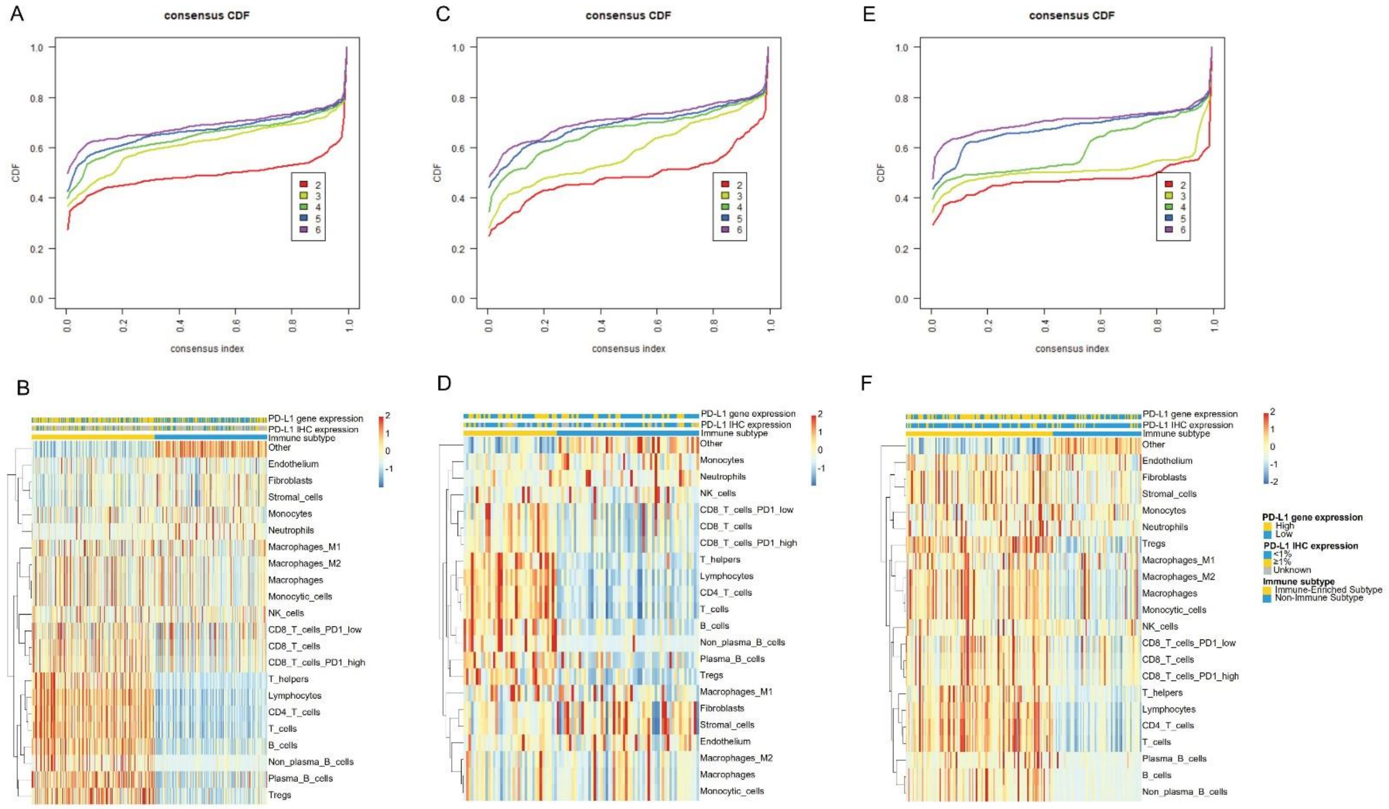

(A) Consensus cumulative distribution function plot testing the cluster number from 2 to 6 to determine the optimal number of clusters in OAK trial. The number of 2 was selected because it showed a relatively horizontal line and a sharper increase at 1. (B) Unsupervised clustering of Kassandra-based cells to classify patients from OAK trial into Immune-Enriched Subtype and Non-Immune Subtype. (C-D) The results of a similar analysis in POPLAR trial. (E-F) The results of a similar analysis in IMvigor210 trial. Data were represented as the z-score of population enrichment across each trial. The “Stromal cells” were calculated as a sum of "Endothelium" and "Fibroblasts" values. The ‘‘Other’’ indicated all cells not deconvolved by Kassandra, mainly including malignant cells and benign epithelial cells. CDF, cumulative distribution function; IHC, immunohistochemistry.

Supplementary Figure 24. Testing the robustness of our proposed PD-L1 predictiveness by immune subtype.

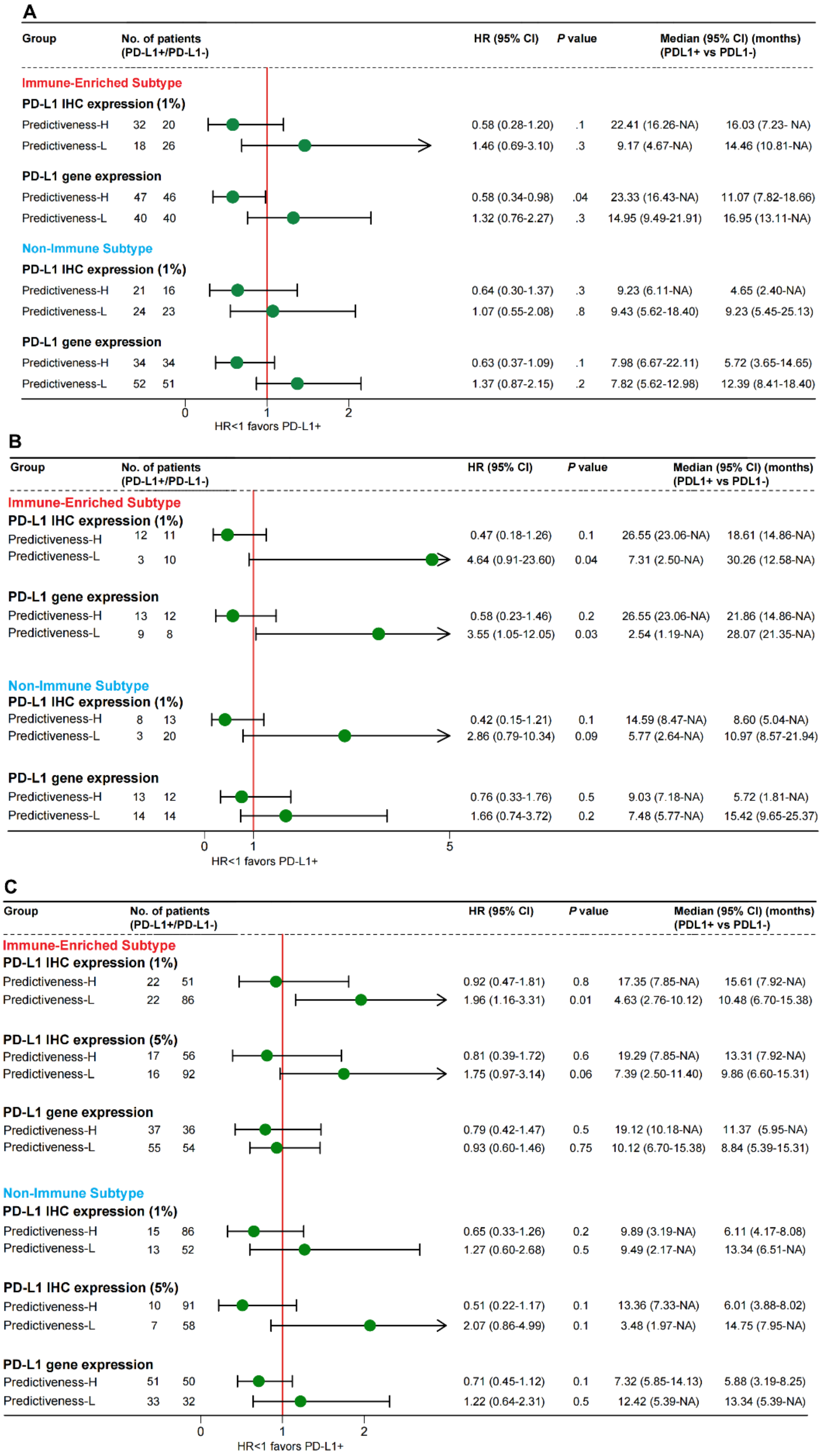

(A) Hazard ratio of overall survival with atezolizumab stratified by PD-L1 immunohistochemistry or gene expression among Predictiveness-High and Predictiveness-Low patients from OAK trial, analyzed separately for Immune-Enriched Subtype and Non-Immune Subtype. (B-C) The results of a similar analysis in POPLAR and IMvigor210 trials, respectively. Error bars represent 95% CI. *P* value indicates log-rank test. The cutoffs of PD-L1 predictiveness score and PD-L1 gene expression were their median values of atezolizumab-treated patients in each trial. The 97.5% limit of a hazard ratio was labeled by an arrow if exceeding three. HR, hazard ratio; CI, confidence interval; IHC, immunohistochemistry; NA, not available.

Supplementary Figure 25. Spearman correlations between PD-L1 and other immune checkpoints in Predictiveness-High and Predictiveness-Low patients across three trials.

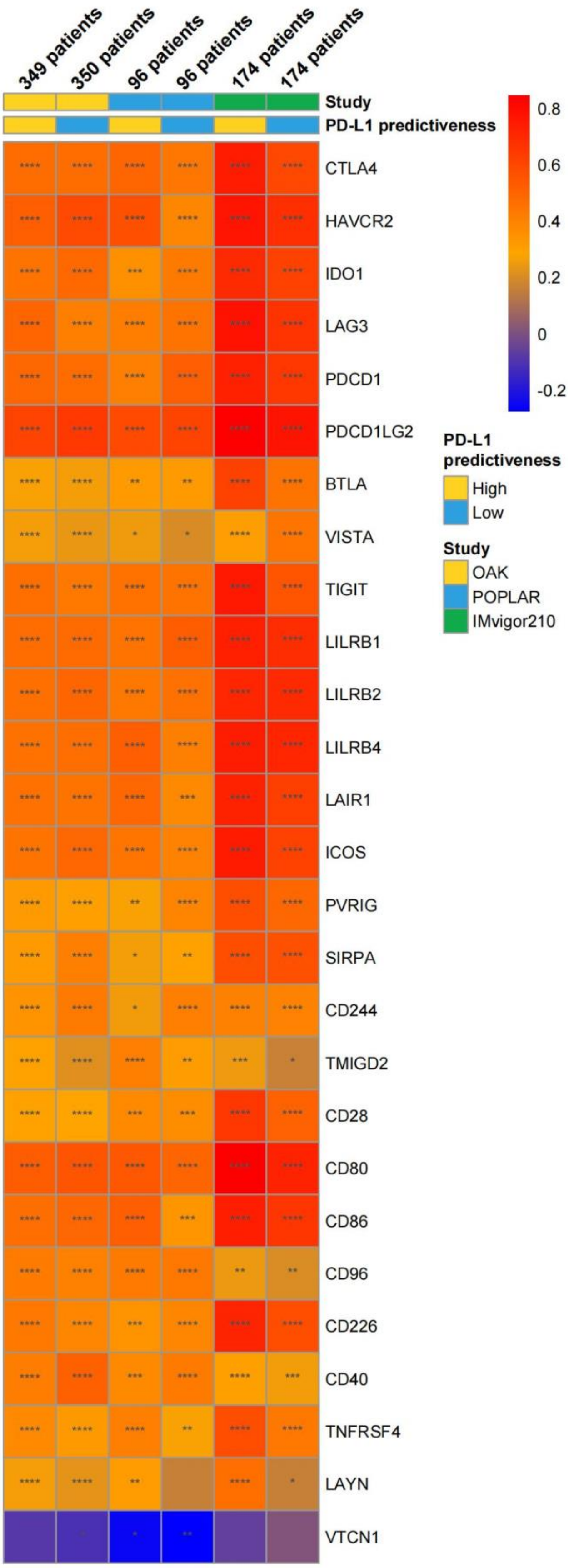

P value indicates the significance test for spearman correlation. The range of P values is labeled within each cell with asterisks. *, P < 0.05; **, P < 0.01; ***, P < 0.001; ****, P < 0.0001.

Supplementary Figure 26. Kaplan-Meier overall survival curve with atezolizumab stratified by inflammatory biomarkers among Predictiveness-High and Predictiveness-Low patients.

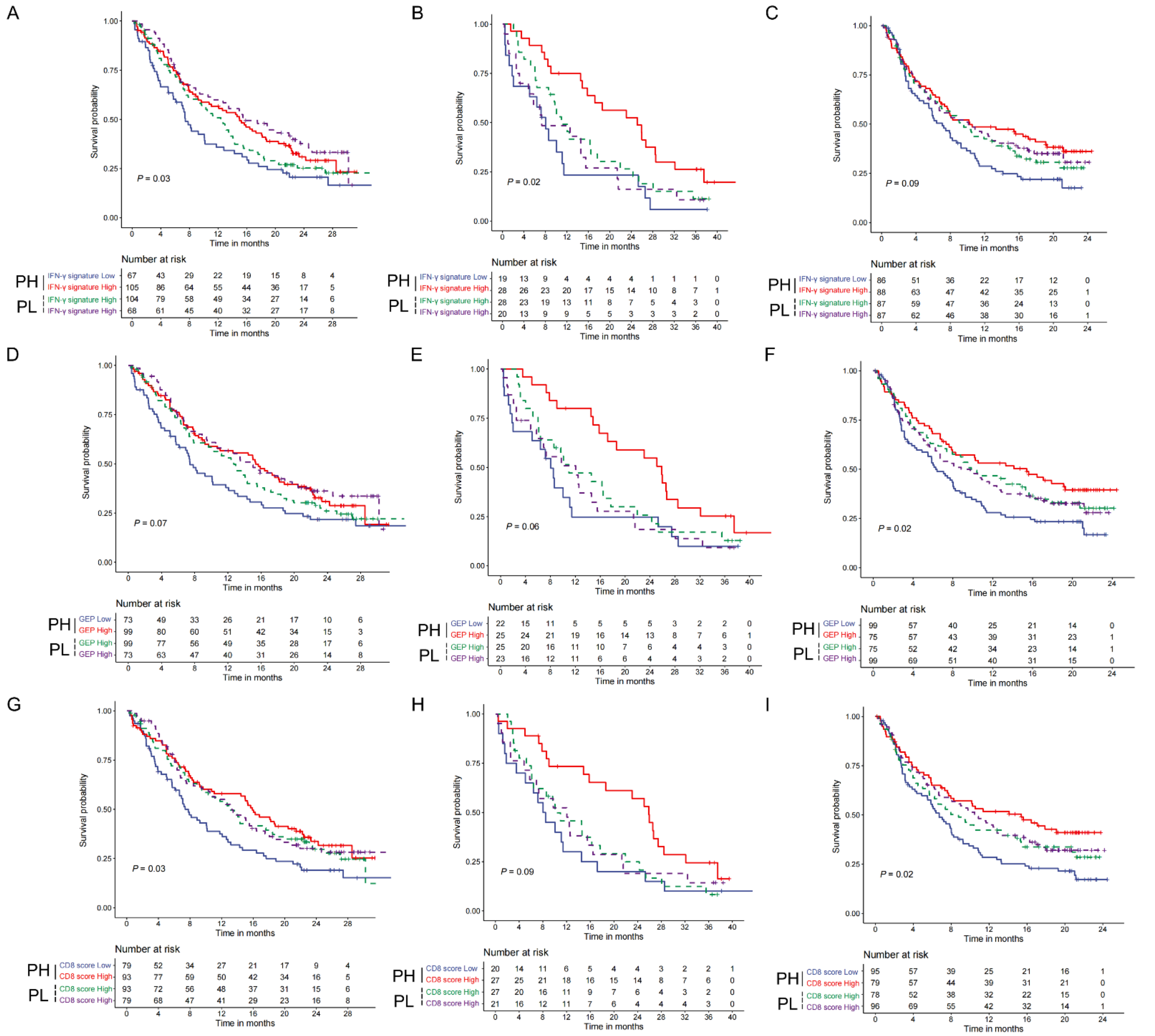

(A-C) Analysis of IFN-γ signature in OAK, POPLAR, and IMvigor210 trials, respectively. (D-F) The results of a similar analysis using T-cell inflamed gene expression profile. (G-I) The results of a similar analysis using CD8 score. *P* value indicates log-rank test. The cutoffs of these variables were their median values of atezolizumab-treated patients in each trial. PH, Predictiveness-High; PL, Predictiveness-Low; IFN, interferon; GEP, T-cell inflamed gene expression profile.

Supplementary Figure 27. Kaplan-Meier overall survival curve with atezolizumab stratified by immune subtype.

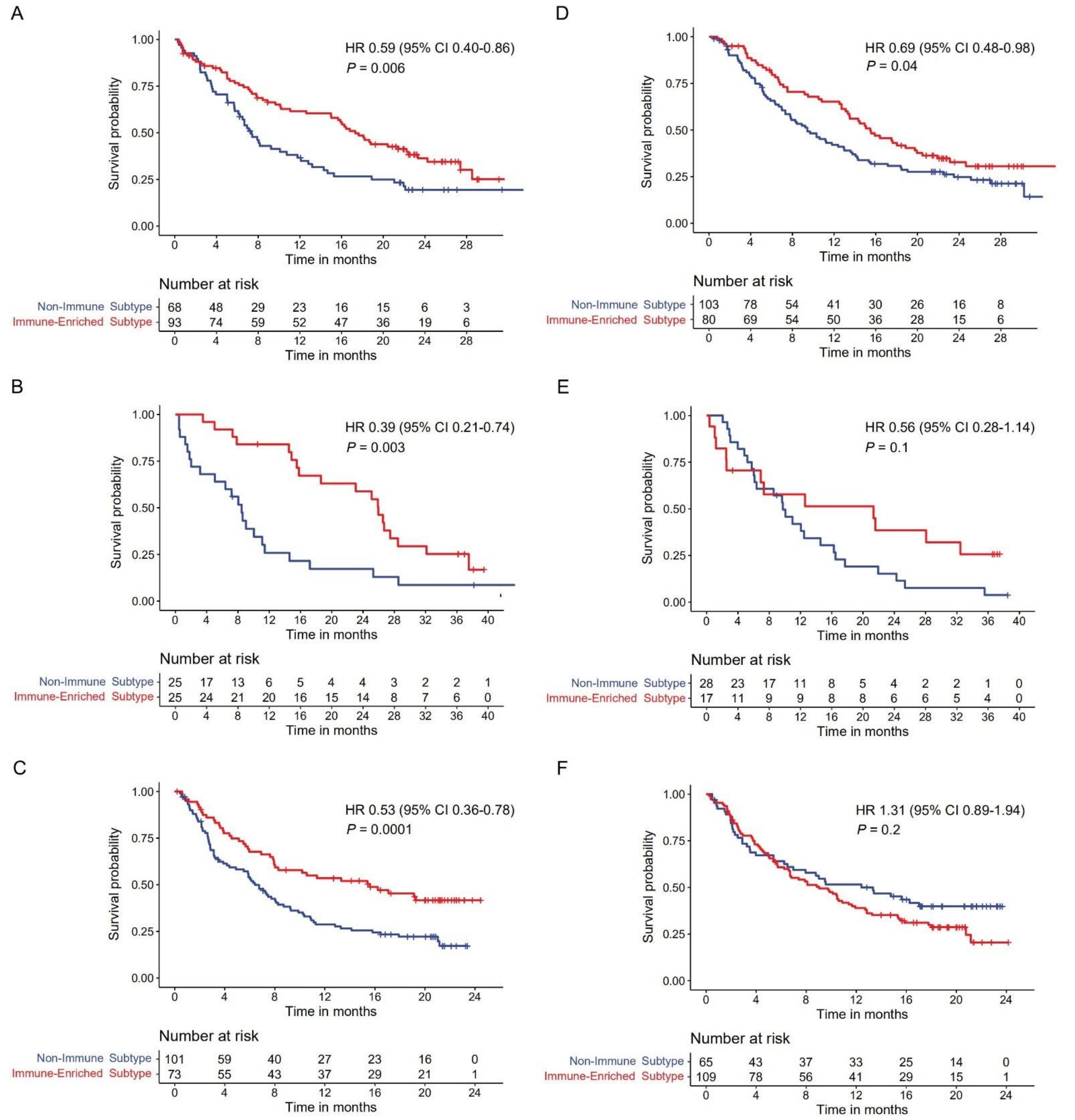

(A-C) Analysis in Predictiveness-High group in OAK, POPLAR, and IMvigor210 trials, respectively. (D-F) The results of a similar analysis in Predictiveness-Low group. *P* value indicates log-rank test. HR, hazard ratio; CI, confidence interval.

Supplementary Figure 28. Testing the robustness of our proposed PD-L1 predictiveness by tumor mutation burden.

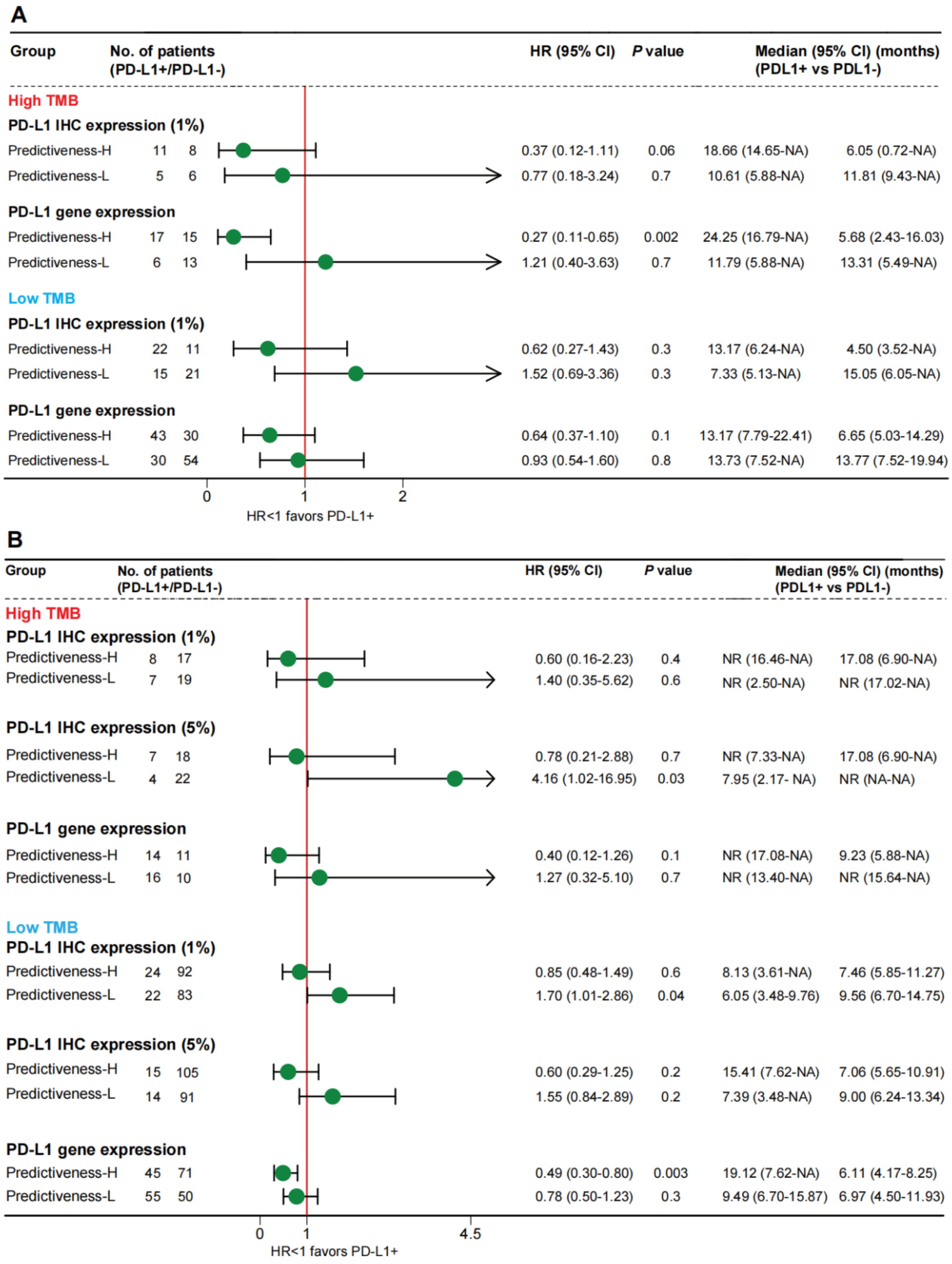

(A) Hazard ratio of overall survival with atezolizumab stratified by PD-L1 immunohistochemistry (IHC) or gene expression among Predictiveness-High and Predictiveness-Low patients from OAK trial, analyzed separately for high tumor mutation burden (TMB) group and low TMB group. (B) The results of a similar analysis in IMvigor210 trial. Error bars represent 95% CI. *P* value indicates log-rank test. The cutoff of TMB was 16 mut/Mb. The cutoffs of PD-L1 predictiveness score and PD-L1 gene expression were their median values of atezolizumab-treated patients. The 97.5% limit of a hazard ratio was labeled by an arrow if exceeding three. HR, hazard ratio; CI, confidence interval; IHC, immunohistochemistry; TMB, tumor mutation burden; NR, not reached; NA, not available.

Supplementary Figure 29. Testing the robustness of our proposed PD-L1 predictiveness by tumor neoantigen burden in IMvigor210 trial.

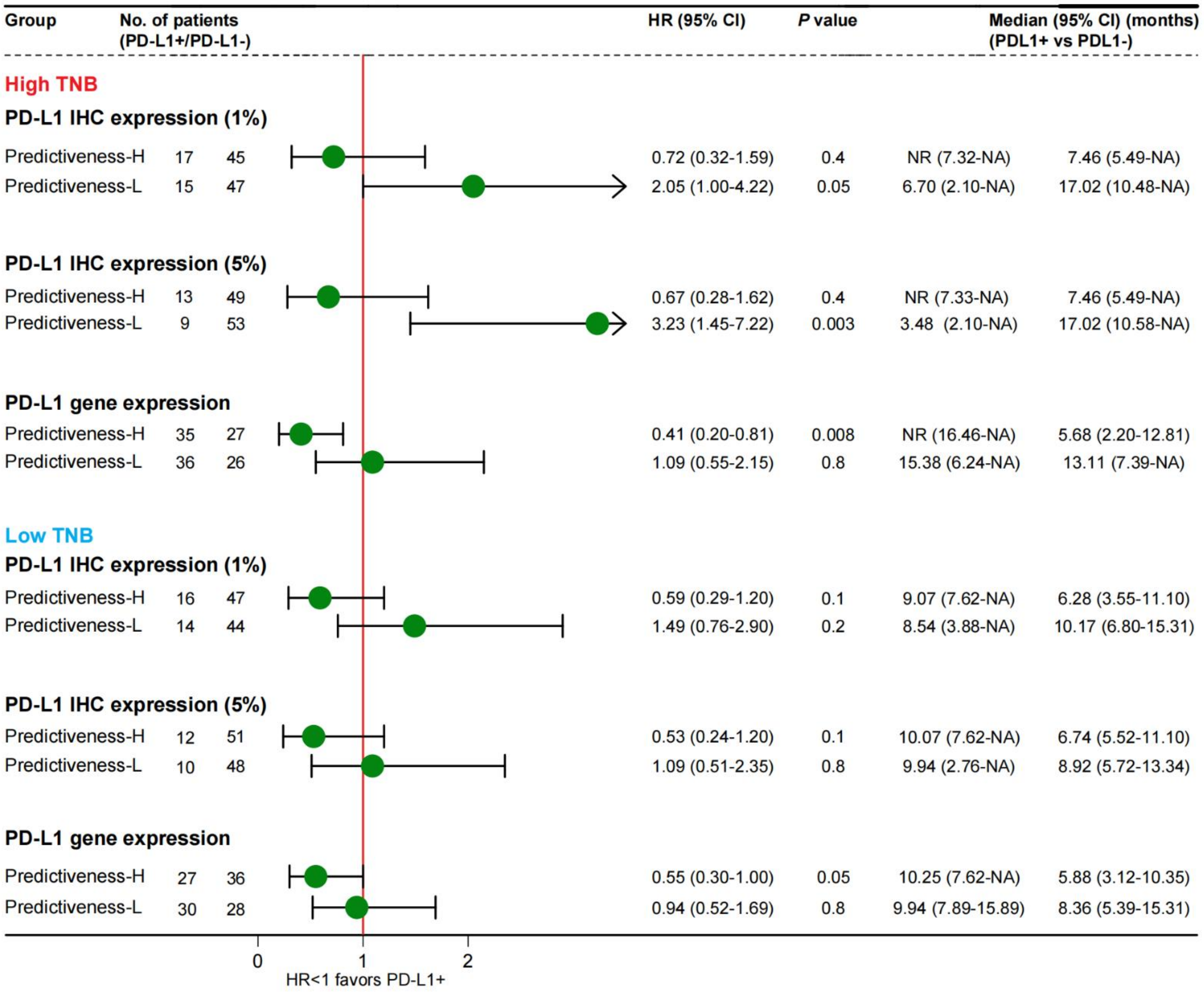

Hazard ratio of overall survival with atezolizumab stratified by PD-L1 immunohistochemistry (IHC) or gene expression among Predictiveness-High and Predictiveness-Low patients, analyzed separately for high tumor neoantigen burden (TNB) group and low TNB group. Error bars represent 95% CI. *P* value indicates log-rank test. The cutoffs of PD-L1 IHC expression were 1% and 5%. The cutoffs of PD-L1 predictiveness score, PD-L1 gene expression, and TNB were their median values of atezolizumab-treated patients. The 97.5% limit of a hazard ratio was labeled by an arrow if exceeding four. HR, hazard ratio; CI, confidence interval; IHC, immunohistochemistry; TNB, tumor neoantigen burden; NR, not reached; NA, not available.

Supplementary Figure 30. Kaplan-Meier overall survival curve with atezolizumab stratified by tumor mutation burden in Predictiveness-High and Predictiveness-Low group.

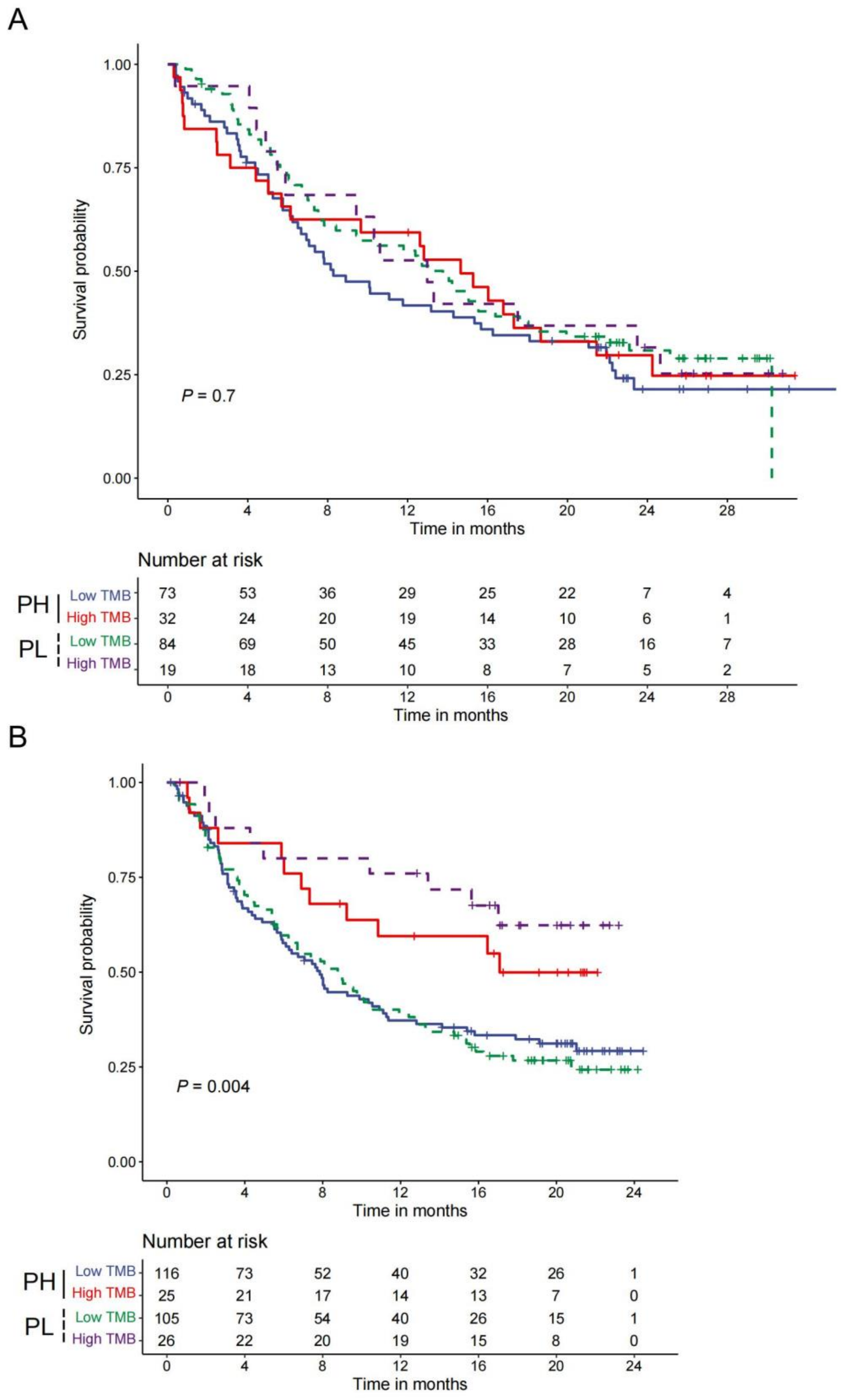

(A) OAK trial (B) IMvigor210 trial. *P* value indicates log-rank test. The cutoff of tumor mutation burden was 16 mut/Mb. The cutoff of PD-L1 predictiveness score was the median value of atezolizumab-treated patients in each trial. PH, Predictiveness-High; PL, Predictiveness-Low; TMB, tumor mutation burden.

Supplementary Figure 31. Kaplan-Meier overall survival curve with atezolizumab stratified by tumor neoantigen burden in Predictiveness-High and Predictiveness-Low group in IMvigor210 trial.

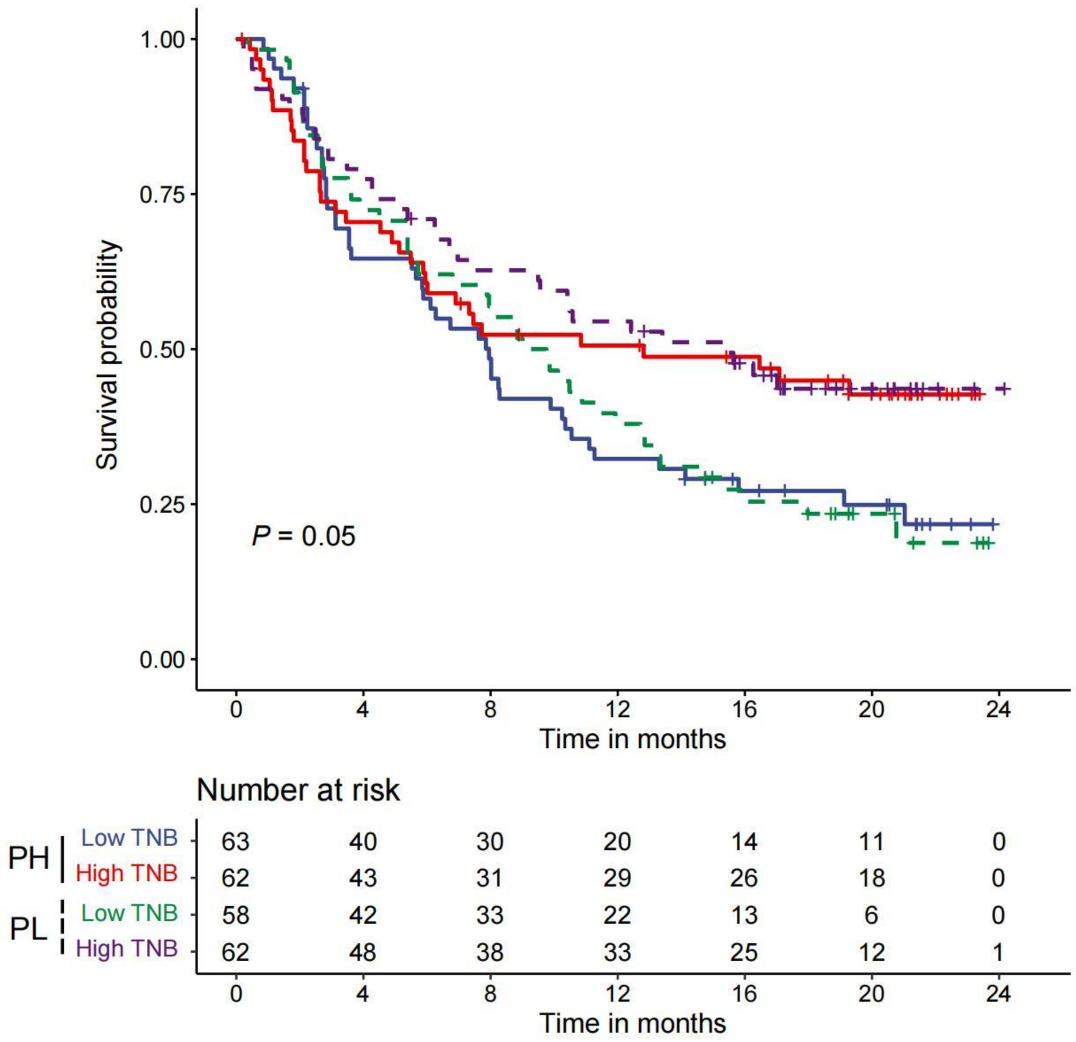

The cutoffs of PD-L1 predictiveness score and tumor neoantigen burden were their median values of atezolizumab-treated patients. *P* value indicates log-rank test. PH, Predictiveness-High; PL, Predictiveness-Low; TNB, tumor neoantigen burden.

Supplementary Figure 32. The fraction of Kassandra-based cells in Predictiveness-High group versus Predictiveness-Low group.

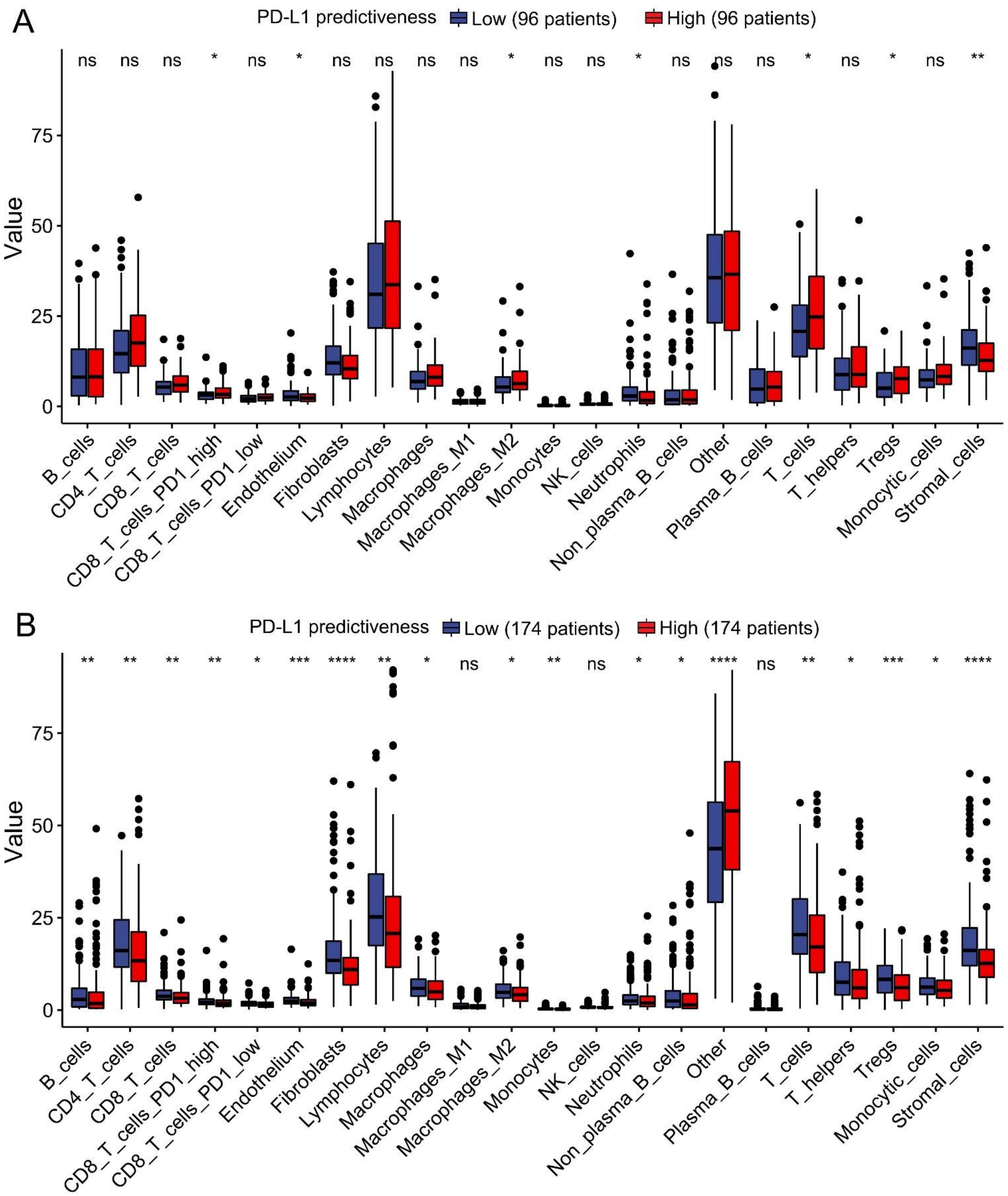

A) POPLAR trial. (B) IMvigor210 trial. The cutoff of PD-L1 predictiveness score was the median value of total intention-to-treat patients in each trial. The “Stromal cells” were calculated as a sum of "Endothelium" and "Fibroblasts" values. The ‘‘Other’’ indicated all cells not deconvolved by Kassandra, mainly including malignant cells and benign epithelial cells. The horizontal line in the boxes represents the median value. The bottom and top of the boxes are the lower and upper quartiles. The whiskers encompass 1.5 times the interquartile range. *P* value indicates Wilcoxon rank-sum test. The range of P values is labeled with asterisks. *, $P < 0.05$; **, $P < 0.01$; ***, $P < 0.001$; ****, $P < 0.0001$.

Supplementary Figure 33. Association of PD-L1 predictiveness score with pathways related to stromal activity.

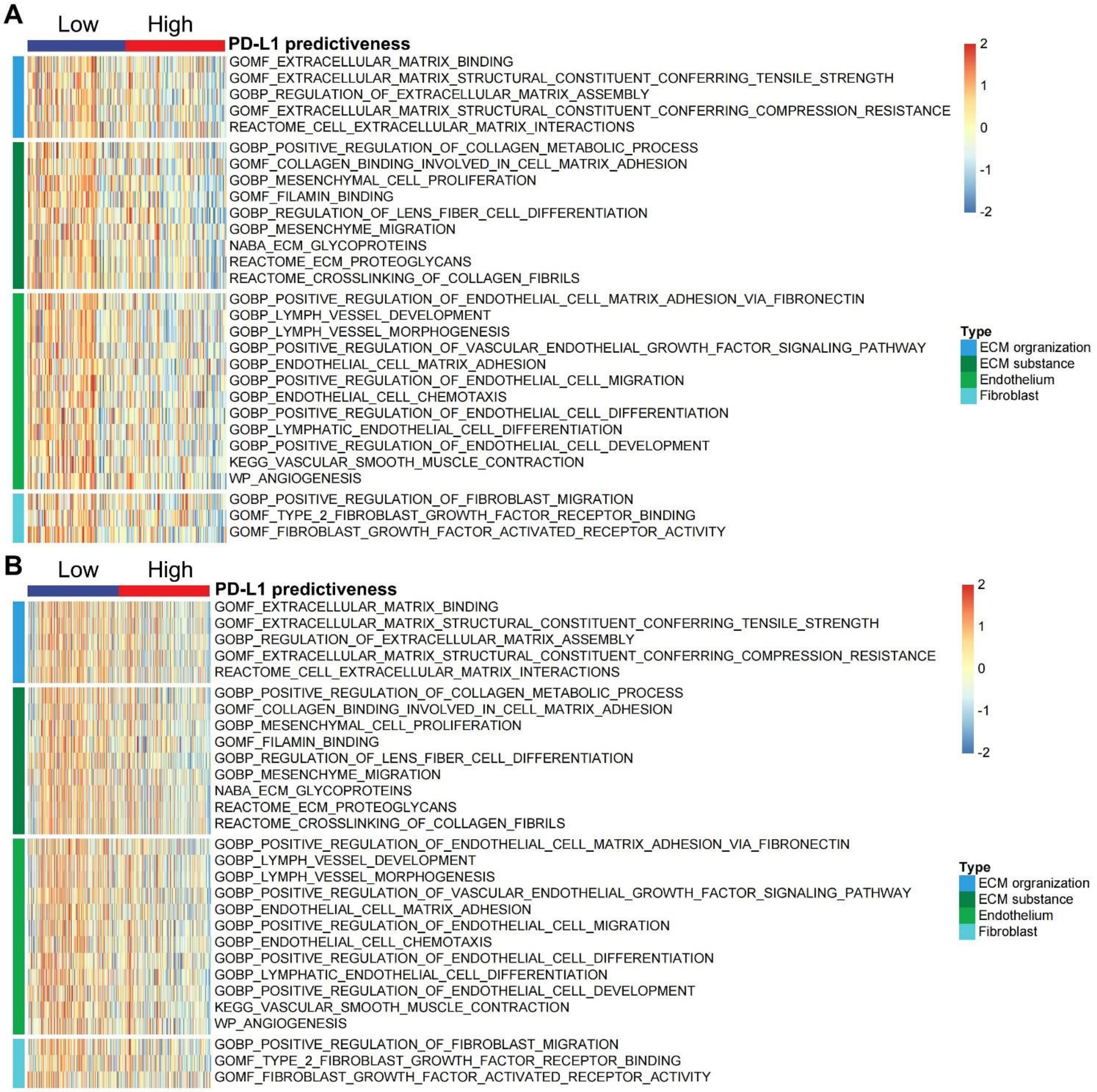

A) POPLAR trial. (B) IMvigor210 trial. Data were represented as the z-score of population enrichment across each trial. ECM, extracellular matrix.

Supplementary Figure 34. Spearman correlation of PD-L1 predictiveness score with fibroblast, endothelium, or stromal score in 31 TCGA cancer types.

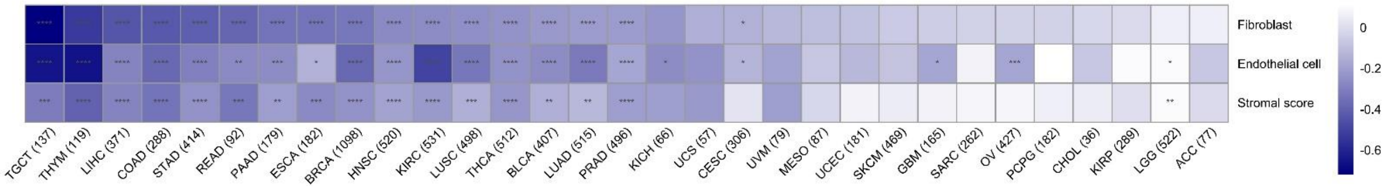

Cancer types were ranked based on the strength of correlation with fibroblast. The levels of fibroblast and endothelial cell were estimated by MCP-counter algorithm, and the stromal score was calculated using ESTIMATE algorithm. *P* value indicates the significance test for spearman correlation. The range of P values is labeled within each cell with asterisks. *, P < 0.05; **, P < 0.01; ***, P < 0.001; ****, P < 0.0001. TGCT, testicular cancer; THYM, thymoma; LIHC, liver cancer; COAD, colon cancer; STAD, stomach cancer; READ, rectal cancer; PAAD, pancreatic cancer; ESCA, esophageal cancer; BRCA, breast cancer; HNSC, head and neck cancer; KIRC, kidney clear cell carcinoma; LUSC, lung squamous cell carcinoma; THCA, thyroid cancer; BLCA, bladder cancer; LUAD, lung adenocarcinoma; PRAD, prostate cancer; KICH, kidney chromophobe; UCS, uterine carcinosarcoma; CESC, cervical cancer; UVM, ocular melanomas; MESO, mesothelioma; UCEC, endometrioid cancer; SKCM, melanoma; GBM, glioblastoma; SARC, sarcoma; OV, ovarian cancer; PCPG, pheochromocytoma or paraganglioma; CHOL, bile duct cancer; KIRP, kidney papillary cell carcinoma; LGG, lower grade glioma; ACC, adrenocortical cancer.

Supplementary Figure 35. Count distribution for patients in IMvigor210 trial.

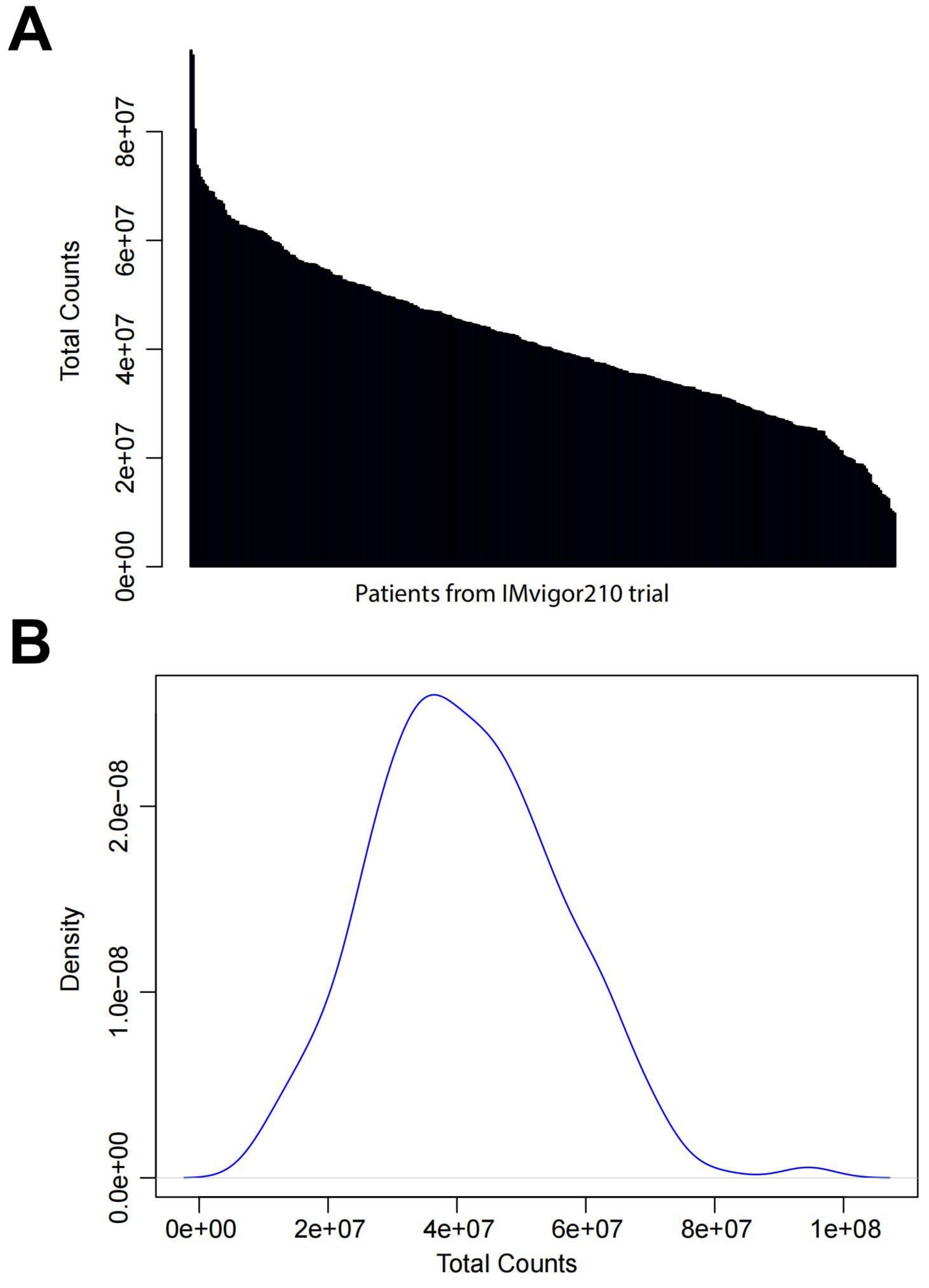

**Supplementary table 1.** Main characteristics of the trials used for evaluation of cross-cancer PD-L1 predictive capacity.												
Trial	Year	Cancer type	Study phase	Treatment line	Target of inhibitor	Treatment strategy	PD-L1 antibody clone	Cell subset for evaluation	PD-L1 scoring method	Total	No. of patients (ICI)	No. of patients (SoC)
Pooled CheckMate-017 and CheckMate-057[1]	2021	NSCLC	3	≥2	PD-1	nivolumab vs docetaxel	28-8	Tumor cells	TPS	854	427	427
OAK[2]	2021		3	≥2	PD-L1	atezolizumab vs docetaxel	SP142	Both	TC/IC	1225	613	612
POPLAR[2]	2021		2	≥2	PD-L1	atezolizumab vs docetaxel	SP142	Both	TC/IC	287	144	143
CheckMate-078[3]	2021		3	≥2	PD-1	nivolumab vs docetaxel	28-8	Tumor cells	TPS	504	338	166
KEYNOTE-010[4]	2021		2/3	≥2	PD-1	pembrolizumab vs docetaxel	22C3	Tumor cells	TPS	1033	690	343
ATTRACTION-3[5]	2022	ESCA	3	≥2	PD-1	nivolumab vs paclitaxel or docetaxel	28-8	Tumor cells	TPS	419	210	209
ESCORT[6]	2020		3	2	PD-1	camrelizumab vs docetaxel or irinotecan	6E8	Tumor cells	TPS	448	228	220
ORIENT-2[7]	2022		2	2	PD-1	sintilimab vs paclitaxel or irinotecan	22C3	Both	TPS/CPS	190	95	95
RATIONALE-302[8,a]	2022		3	2	PD-1	tislelizumab vs paclitaxel, docetaxel, or irinotecan	SP263	Both	TAP	512	256	256

KEYNOTE-181[9,a]	2020		3	2	PD-1	pembrolizumab vs paclitaxel, docetaxel, or irinotecan	22C3	Both	CPS	628	314	314
KEYNOTE-040[10]	2019	HNSC	3	≥2	PD-1	pembrolizumab vs methotrexate, docetaxel, or cetuximab	22C3	Both	CPS	495	247	248
CheckMate 141[11]	2018		3	≥2	PD-1	nivolumab vs methotrexate, docetaxel, or cetuximab	28-8	Tumor cells	TPS	361	240	121
EAGLE[12]	2020		3	≥2	PD-L1	durvalumab vs cetuximab, taxane, methotrexate, or fluoropyrimidine	SP263	Tumor cells	TC	489	240	249
JAVELIN Gastric 300[13]	2018	STAD[b]	3	3	PD-L1	avelumab vs paclitaxel or irinotecan	73-10	Tumor cells	TPS	371	185	186
KEYNOTE-061[14]	2018		3	≥2	PD-1	pembrolizumab vs paclitaxel	22C3	Both	CPS	592	296	296
KEYNOTE-045[15]	2017	BLCA[c]	3	≥2	PD-1	pembrolizumab vs paclitaxel, docetaxel, or vinflunine	22C3	Both	CPS	542	270	272
IMvigor211[16]	2021		3	≥2	PD-L1	atezolizumab vs vinflunine, paclitaxel, or docetaxel	SP142	Tumor-infiltrating immune cells	IC	931	467	464

KEYNOTE-002[17]	2017	SKCM	2	≥2	PD-1	pembrolizumab vs paclitaxel plus carboplatin, paclitaxel, carboplatin, dacarbazine, or temozolomide	22C3	Tumor cells	TPS	540	361	179
CheckMate-037[18,a]	2017		3	≥2	PD-1	nivolumab vs dacarbazine or carboplatin plus paclitaxel	22C3	Tumor cells	TPS	405	272	133
JAVELIN Ovarian 200[19]	2021	OV	3	≥2	PD-L1	avelumab vs pegylated liposomal doxorubicin	SP263	Both	TC/IC	378	188	190
NINJA[20]	2021		3	≥2	PD-1	nivolumab vs gemcitabine or pegylated liposomal doxorubicin	NR	Tumor cells	TPS	316	157	159
CheckMate-331[21]	2021	SCLC	3	≥2	PD-1	nivolumab vs topotecan or amrubicin	28-8	Both	CPS	569	284	285
CheckMate-025[22]	2015	KIRC	3	≥2	PD-1	nivolumab vs everolimus	22C3	Tumor cells	TPS	821	410	411
KEYNOTE-119[23]	2021	BRCA	3	≥2	PD-1	pembrolizumab vs capecitabine, eribulin, gemcitabine, or vinorelbine	22C3	Both	CPS	622	312	310

Imblaze-370[24]	2019	COAD	3	3	PD-L1	atezolizumab vs regorafenib	SP142	Tumor-infiltrating immune cells	IC	180	90	90
PROMISE-meso[25]	2020	MESO	3	≥2	PD-1	pembrolizumab vs gemcitabine or vinorelbine	SP263/E1L3N	Tumor cells	TPS	144	73	71
CheckMate-143[26]	2020	GBM	3	2	PD-1	nivolumab vs bevacizumab	28-8	Tumor cells	TPS	369	184	185

[a]All included trials used threshold of 1% (TPS/TC/IC) or 1 (CPS) to define PD-L1 positivity, except CheckMate-037 (TPS of 5%), RATIONALE-302 (TAP of 10%), KEYNOTE-181 (CPS of 10).

[b]There were a small proportion of patients with gastro-esophageal junction cancer in STAD trials.

[c]Trials of BLCA investigated urothelial cancer and primary tumor sites of most patients were bladder.

Abbreviations: ICI, immune checkpoint inhibitor; SoC, standard-of-care; COAD, colon cancer; BRCA, breast cancer; STAD, stomach cancer; SKCM, melanoma; ESCA, esophageal cancer; OV, ovarian cancer; HNSC, head and neck cancer; NSCLC, non-small-cell lung cancer; BLCA, bladder cancer; KIRC, kidney clear cell carcinoma; SCLC, small-cell lung cancer; MESO, mesothelioma; GBM, glioblastoma; TPS, tumor proportional score, CPS, combined positive score, TC, tumor cells, IC, tumor-infiltrating immune cells; NR, not reported.

Supplementary table 2. Hazard ratios of overall survival for anti-PD-1/PD-L1 versus standard treatment stratified by PD-L1 expression.

Cancer type	Trial and published year	No. of patients (ICI)	No. of patients (SoC)	Hazard ratio	2.5% Limit	97.5% Limit
**COAD**						
PD-L1+	Imblaze-370 2019	35	31	0.8	0.45	1.43
PD-L1-	Imblaze-370 2019	42	40	1.81	1.05	3.13
**BRCA**						
PD-L1+	KEYNOTE-119 2021	203	202	0.85	0.69	1.05
PD-L1-	KEYNOTE-119 2021	109	108	1.27	0.95	1.7
**STAD**						
PD-L1+	JAVELIN Gastric 300 2018	46	39	0.94	0.57	1.55
	KEYNOTE-061 2018	196	199	0.82	0.66	1.03
	**Pooled estimate**			**0.84**	**0.68**	**1.03**
PD-L1-	JAVELIN Gastric 300 2018	111	121	1.22	0.91	1.64
	KEYNOTE-061 2018	99	96	1.2	0.89	1.63
	**Pooled estimate**			**1.21**	**0.98**	**1.49**
**SKCM**						
PD-L1+	KEYNOTE-002 2017	196	98	0.76	0.57	1.03
	CheckMate-037 2017	134	67	0.73	0.49	1.09
	**Pooled estimate**			**0.75**	**0.59**	**0.95**
PD-L1-	KEYNOTE-002 2017	94	40	0.94	0.62	1.44
	CheckMate-037 2017	132	63	1.15	0.82	1.62
	**Pooled estimate**			**1.06**	**0.81**	**1.38**

**ESCA**						
PD-L1+	ATTRACTION-3 2022	101	102	0.7	0.52	0.95
	ESCORT 2020	93	98	0.58	0.42	0.81
	ORIENT-2 2022[a]	30	24	0.88	0.45	1.71
	ORIENT-2 2022[a]	63	50	0.73	0.47	1.12
	RATIONALE-302 2022	89	68	0.54	0.36	0.79
	KEYNOTE-181 2020	107	115	0.7	0.52	0.94
	**Pooled estimate**			**0.66**	**0.57**	**0.77**
PD-L1-	ATTRACTION-3 2022	109	107	0.85	0.63	1.14
	ESCORT 2020	129	118	0.82	0.62	1.09
	ORIENT-2 2022[a]	44	43	0.79	0.49	1.28
	ORIENT-2 2022[a]	11	17	1.3	0.51	3.27
	RATIONALE-302 2022	116	140	0.82	0.62	1.09
	KEYNOTE-181 2020	201	196	1	0.81	1.24
	**Pooled estimate**			**0.89**	**0.78**	**1.01**
**OV**						
PD-L1+	JAVELIN Ovarian 200 2021	100	88	0.83	0.57	1.23
	NINJA 2021	65	58	1.09	0.73	1.64
	**Pooled estimate**			**0.94**	**0.71**	**1.25**
PD-L1-	JAVELIN Ovarian 200 2021	70	77	1.38	0.91	2.08
	NINJA 2021	88	101	1.07	0.78	1.46
	**Pooled estimate**			**1.17**	**0.91**	**1.51**
**HNSC**						
PD-L1+	KEYNOTE-040 2019	196	191	0.74	0.58	0.93

	CheckMate 141 2018	96	63	0.55	0.39	0.78
	EAGLE 2020	169	178	0.96	0.77	1.22
	**Pooled estimate**			**0.75**	**0.55**	**1.01**
PD-L1-	KEYNOTE-040 2019	50	54	1.28	0.8	2.1
	CheckMate 141 2018	76	40	0.73	0.49	1.09
	EAGLE 2020	71	71	0.69	0.48	1
	**Pooled estimate**			**0.84**	**0.59**	**1.2**
**NSCLC**						
PD-L1+	Pooled CheckMate-017 and CheckMate-057 2021	185	179	0.61	0.49	0.76
	OAK 2021	347	337	0.78	0.66	0.93
	POPLAR 2021	93	102	0.7	0.51	0.95
	CheckMate-078 2021	168	84	0.7	0.53	0.92
	KEYNOTE-010 2021[b]	690	343	0.7	0.61	0.8
	**Pooled estimate**			**0.7**	**0.65**	**0.77**
PD-L1-	Pooled CheckMate-017 and CheckMate-057 2021	163	153	0.76	0.61	0.96
	OAK 2021	260	271	0.78	0.65	0.94
	POPLAR 2021	51	41	0.88	0.55	1.41
	CheckMate-078 2021	138	27	0.76	0.55	1.06
	**Pooled estimate**			**0.78**	**0.69**	**0.88**
**BLCA**						
PD-L1+	KEYNOTE-045 2017	NA	NA	0.61	0.43	0.86
	IMvigor211 2021	316	309	0.84	0.7	1
	**Pooled estimate**			**0.74**	**0.55**	**1.01**
PD-L1-	KEYNOTE-045 2017	NA	NA	0.89	0.66	1.2

	IMvigor211 2021	151	155	0.76	0.59	0.78
	**Pooled estimate**			**0.78**	**0.69**	**0.89**
**KIRC**						
PD-L1+	CheckMate-025 2015	97	87	0.79	0.53	1.17
PD-L1-	CheckMate-025 2015	276	299	0.77	0.61	0.98
**SCLC**						
PD-L1+	CheckMate-331 2021	78	68	0.96	0.67	1.38
PD-L1-	CheckMate-331 2021	93	82	0.91	0.66	1.25
**MESO**						
PD-L1+	PROMISE-meso 2020[c]	31	32	1.09	0.57	2.09
	PROMISE-meso 2020[c]	32	34	1.47	0.69	3.11
	**Pooled estimate**			**1.24**	**0.76**	**2.03**
PD-L1-	PROMISE-meso 2020[c]	36	30	0.96	0.53	1.75
	PROMISE-meso 2020[c]	19	17	0.72	0.26	2
	**Pooled estimate**			**0.89**	**0.53**	**1.49**
**GBM**						
PD-L1+	CheckMate-143 2020	48	35	1.35	0.83	2.19
PD-L1-	CheckMate-143 2020	107	114	0.97	0.72	1.3

[a]ORIENT-2 study used two scoring methods, tumor-proportion score (TPS) and combined positive score (CPS), to determine PD-L1 protein expression.

[b]The KEYNOTE-010 study was conducted in patients with positive PD-L1 expression (TPS≥1%). Therefore, there were no PD-L1-negative entries in this study.

[c]PROMISE-meso study used two antibody clones, SP263 and E1L3N, to determine PD-L1 protein expression.

Abbreviations: ICI, immune checkpoint inhibitor; SoC, standard-of-care; COAD, colon cancer; BRCA, breast cancer; STAD, stomach cancer; SKCM, melanoma; ESCA, esophageal cancer; OV, ovarian cancer; HNSC, head and neck cancer; NSCLC, non-small-cell lung cancer; BLCA, bladder cancer; KIRC, kidney clear cell carcinoma; SCLC, small-cell lung cancer; MESO, mesothelioma; GBM, glioblastoma; NR, not reported.

Supplementary table 3. Hazard ratios of progression-free survival for anti-PD-1/PD-L1 versus standard treatment stratified by PD-L1 expression.

Cancer type	Trial and published year	No. of patients (ICI)	No. of patients (SoC)	Hazard ratio	2.5% Limit	97.5% Limit
**COAD**		90	90			
PD-L1+	Imblaze-370 2019	35	31	0.71	0.42	1.21
PD-L1-	Imblaze-370 2019	42	40	2.17	1.31	3.59
**STAD**						
PD-L1+	JAVELIN Gastric 300 2018	46	39	1.75	1.02	3.01
	KEYNOTE-061 2018	196	199	1.27	1.03	1.57
	**Pooled estimate**			**1.35**	**1.06**	**1.72**
PD-L1-	JAVELIN Gastric 300 2018	111	121	1.95	1.44	2.63
	KEYNOTE-061 2018	99	96	2.05	1.5	2.79
	**Pooled estimate**			**2**	**1.61**	**2.48**
**HNSC**						
PD-L1+	KEYNOTE-040 2019	196	191	1.01	0.81	1.26
	CheckMate 141 2018	96	63	0.59	0.41	0.84
	**Pooled estimate**			**0.79**	**0.47**	**1.33**
PD-L1-	CheckMate 141 2018	76	40	1.13	0.75	1.71
**OV**						
PD-L1+	JAVELIN Ovarian 200 2021	100	88	1.45	1.03	2.04
PD-L1-	JAVELIN Ovarian 200 2021	70	77	1.66	1.15	2.4
**NSCLC**						

PD-L1+	Pooled CheckMate-017 and CheckMate-057 2021	185	179	0.66	0.53	0.84
	OAK 2021	347	337	0.87	0.73	1.02
	POPLAR 2021	93	102	0.85	0.63	1.16
	CheckMate-078 2021	168	84	0.74	0.55	0.98
	KEYNOTE-010 2021[a]	690	343	0.84	0.73	0.96
	**Pooled estimate**			**0.81**	**0.74**	**0.89**
PD-L1-	Pooled CheckMate-017 and CheckMate-057 2021	163	153	0.99	0.78	1.26
	OAK 2021	260	271	1.11	0.93	1.34
	POPLAR 2021	51	41	1.12	0.72	1.77
	CheckMate-078 2021	138	27	0.78	0.56	1.07
	**Pooled estimate**			**1.01**	**0.87**	**1.17**
**SCLC**						
PD-L1+	CheckMate-331 2021	78	68	1.52	1.06	2.18
PD-L1-	CheckMate-331 2021	93	82	1.68	1.23	2.3
**ESCA**						
PD-L1+	ESCORT 2020	93	98	0.6	0.43	0.84
	ORIENT-2 2022[b]	30	24	1.08	0.59	2.02
	ORIENT-2 2022[b]	63	50	1.03	0.68	1.57
	KEYNOTE-181 2020[c]	107	115	0.73	0.54	0.97
	**Pooled estimate**			**0.79**	**0.61**	**1.02**
PD-L1-	ESCORT 2020	129	118	0.79	0.59	1.05
	ORIENT-2 2022[b]	44	43	1.11	0.67	1.82
	ORIENT-2 2022[b]	11	17	1.21	0.43	3.52
	**Pooled estimate**			**0.9**	**0.67**	**1.2**

**MESO**						
PD-L1+	PROMISE-meso 2020[d]	31	32	1.1	0.63	1.94
	PROMISE-meso 2020[d]	32	34	1.06	0.63	1.8
	**Pooled estimate**			**1.08**	**0.73**	**1.58**
PD-L1-	PROMISE-meso 2020[d]	36	30	0.76	0.44	1.3
	PROMISE-meso 2020[d]	19	17	1.26	0.56	2.83
	**Pooled estimate**			**0.89**	**0.56**	**1.41**

[a]The KEYNOTE-010 study was conducted in patients with positive PD-L1 expression (TPS≥1%). Therefore, there were no PD-L1-negative entries in this study.

[b]ORIENT-2 study used two scoring methods, tumor-proportion score (TPS) and combined positive score (CPS), to determine PD-L1 protein expression.

[c]For PFS in KEYNOTE-181 trial, PD-L1-negtive results was not reported.

[d]PROMISE-meso study used two antibody clones, SP263 and E1L3N, to determine PD-L1 protein expression.

Abbreviations: ICI, immune checkpoint inhibitor; SoC, standard-of-care; COAD, colon cancer; STAD, stomach cancer; ESCA, esophageal cancer; OV, ovarian cancer; HNSC, head and neck cancer; NSCLC, non-small-cell lung cancer; SCLC, small-cell lung cancer; MESO, mesothelioma.

Supplementary table 4. Evaluation of PD-L1 predictive capacity by target of immune checkpoint.

Cancer type	Target	Number of trial	HR (95% CI) (PD-L1+)	I^2	HR (95% CI) (PD-L1-)	I^2	HRD	*P* for interaction
**Overall survival**								
BLCA	PD-1	1	0.61 (0.43-0.86)	NA	0.89 (0.66-1.20)	NA	0.28	0.11
	PD-L1	1	0.84 (0.70-1.00)	NA	0.76 (0.66-0.87)	NA	-0.08	0.39
HNSC	PD-1	2	0.66 (0.49-0.87)	48%	0.95 (0.55-1.61)	68%	0.29	0.24
	PD-L1	1	0.96 (0.76-1.21)	NA	0.69 (0.48-1.00)	NA	-0.27	0.14
NSCLC	PD-1	4	0.68 (0.61-0.75)	0%	0.76 (0.63-0.92)	0%	0.08	0.3
	PD-L1	2	0.76 (0.65-0.88)	0%	0.79 (0.67-0.94)	0%	0.03	0.72
OV	PD-1	1	1.09 (0.73-1.63)	NA	1.07 (0.78-1.46)	NA	-0.02	0.94
	PD-L1	1	0.83 (0.57-1.22)	NA	1.38 (0.91-1.29)	NA	0.55	0.08
STAD	PD-1	1	0.82 (0.66-1.02)	NA	1.2 (0.89-1.62)	NA	0.38	0.05
	PD-L1	1	0.94 (0.57-1.55)	NA	1.22 (0.91-1.64)	NA	0.28	0.38
**Progression-free survival**								
NSCLC	PD-1	4	0.76 (0.65-0.89)	39%	0.9 (0.72-1.13)	26%	0.14	0.23
	PD-L1	2	0.87 (0.75-1.00)	0%	1.11 (0.94-1.32)	0%	0.24	0.03
STAD	PD-1	1	1.27 (1.03-1.57)	NA	2.05 (1.50-2.80)	NA	0.78	0.01
	PD-L1	1	1.75 (1.02-3.01)	NA	1.95 (1.44-2.64)	NA	0.2	0.73

We evaluated PD-L1 predictive capacity by target of immune checkpoint (PD-1 or PD-L1) in five cancer types where trial data for anti-PD-1 and anti-PD-L1 therapies were available. NSCLC and gastric cancer showed consistent OS PD-L1 predictive capacity for the two targets, but PD-L1 predictive capacity seems to be different between the two targets in bladder cancer (PD-1 0.28, PD-L1 -0.08), head and neck cancer (PD-1 0.29, PD-L1 -0.27), and ovarian cancer (PD-1 -0.02, PD-L1 0.55). Abbreviations: HR, hazard ratio; CI, confidence interval; HRD, hazard ratio difference; STAD, stomach cancer; OV, ovarian cancer; HNSC, head and neck cancer; NSCLC, non-small-cell lung cancer; BLCA, bladder cancer; NA, not available.

Supplementary table 5. Median values of four previously established immunotherapy biomarkers across a cancer type.

Variable	COAD	BRCA	STAD	SKCM	ESCA	NSCLC	KIRC	MESO	GBM
Tumor mutation burden	2.84	0.91	3.04	8.34	2.12	5.71	1.34	0.63	1.34
Tumor neoantigen burden	143	48	137	210	128	225	56	28	55
IFN-γ signature	0.21	-0.01	-0.03	0.24	0.11	0.07	0.07	0.06	-0.02
CD8 score	0.06	0.03	0.1	0.17	0.17	0	-0.01	0	0.03

Small-cell lung cancer was excluded due to lack of molecular data from TCGA. We also excluded bladder cancer, ovarian cancer, and head and neck cancer from this analysis due to a substantial heterogeneity between trials. Abbreviations: COAD, colon cancer; BRCA, breast cancer; STAD, stomach cancer; SKCM, melanoma; ESCA, esophageal cancer; OV, ovarian cancer; NSCLC, non-small-cell lung cancer; KIRC, kidney clear cell carcinoma; MESO, mesothelioma; GBM, glioblastoma; IFN, interferon.

**Supplementary table 6.** Median TPM values of 1058 immune-related genes across a cancer type.									
**Variable**	**COAD**	**BRCA**	**STAD**	**SKCM**	**ESCA**	**NSCLC**	**KIRC**	**MESO**	**GBM**
A2M	5.82	7.22	7.16	8.25	6.6	7.1	8.66	6.74	8.13
ABO	3.32	-1.6	2.91	-4.04	2.58	1.72	1.52	0.4	-3.24
ACP5	4.92	5.87	5.36	7.42	5.29	6.28	5.67	3.99	2.38
ACTL6A	5.85	5.72	5.4	5.3	6.05	5.91	4.63	5.01	5.46
ACTR3B	2.86	2.88	2.23	2.78	2.04	2.43	1.99	2.1	3.8
ACVRL1	4.11	2.98	3.28	2.49	2.56	3.01	4.67	2.6	3.17
ADAM9	5.56	5	5.89	4.22	6.05	5.78	5.86	5.88	6.01
ADAMDEC1	3.33	1.43	2.43	0.91	1.51	2.29	0.61	0.33	0.6
ADAMTS1	2.08	3.16	3.25	3.31	3.51	3.14	5.1	3.51	3.13
ADAR	5.8	6.84	6.38	6.35	6.69	6.31	5.88	5.81	6.02
ADCY4	1.5	1.94	2.15	0.7	2.07	1.41	3.13	1.51	1.63
ADCY7	2.06	2.29	3	2.01	3.58	3.27	2.2	3.11	2.31
ADD3	5.77	4.2	5.65	4.24	5.63	4.81	5.45	3.69	5.34
AIMP2	5.5	4.76	4.85	5.16	4.78	4.88	4.07	4.77	5.31
AKT1	6.28	6.74	6.3	6.81	6.51	6.44	5.92	5.89	6.64
ALKBH7	6.34	5.91	5.81	6.26	5.53	5.66	5.74	6.82	6.23
ALOX5AP	3.72	4.75	4.71	3.31	3.84	5.15	4.21	5.35	6.86
AMICA1	1.91	2.24	2.37	2.38	1.55	3.26	3.52	2.96	0.74
AMPD3	2.18	2.24	2.21	1.15	2.67	2.74	2.84	2	2.55
ANAPC15	5.73	5.4	4.87	5.13	4.95	5.41	4.9	5.33	5.74
ANP32E	4.47	4.67	4.79	4.86	5.14	5.17	4.76	4.2	5.02
ANPEP	3.35	3.82	4.46	2.48	3.27	3.02	7.24	3.7	1.98
APITD1	4.11	4.38	3.68	3.89	3.81	4.25	4.7	3.84	4.32
APLNR	2.49	3.58	3.57	2.31	2.53	1.67	4.26	2.6	5.59
APOBEC3B	3	2.95	3.09	3.32	3.92	3.57	1.41	2.44	2.21
APOC1	5.66	7.55	6.27	8.56	5.28	7.96	8.15	7.55	9.25
APOD	2.81	7.04	5.05	9.59	3.18	4.14	2.32	4.18	7.38
APOE	5.87	7.87	7.59	10.49	6.43	7.87	8.64	8.4	10.75

APOO	4.56	4.82	4.19	5.03	4.25	4.31	3.83	3.92	5.62
APP	8.58	8.65	8.14	8.34	8.34	8.11	9.12	8.25	9.16
ARHGAP1	5.58	6.2	6.07	5.69	6.23	5.68	5.9	5.96	5.88
ARHGAP12	3.97	4.12	4.29	3.49	4.13	3.77	3.67	3.19	4.49
ARHGAP15	1.31	2.42	2.25	2.42	1.35	2.42	3.77	2.59	2.54
ARHGAP25	1.37	2.72	2.42	2.44	2.34	3.13	4.06	2.82	3.16
ARHGAP30	1.61	2.89	2.47	2.5	2.37	3.06	3.3	3.12	2.78
ARHGDIA	8.15	7.85	8	8.55	8.25	7.82	7.7	8.44	7.96
ARHGEF3	2.98	4.18	3.64	2.83	3.73	3.5	3.62	3.88	4.63
ARRB2	4.99	4.92	5.08	4.88	4.73	5.2	5.52	5.65	6.3
ASPM	2.53	2.3	2.72	2.44	3.73	2.36	-0.17	3.3	1.84
ATAD2	3.54	4.03	3.73	3.24	4.61	3.55	1.44	2.53	2.93
ATP6V0A1	4.77	4.9	4.73	5.86	5.21	4.61	5.37	4.78	6
ATP6V0B	7.21	7.61	7.1	7.59	7.21	7.33	6.78	7.45	8.03
AURKA	4.92	3.74	4.49	4.17	4.66	4.07	1.58	2.96	3.36
AURKB	4.92	3.56	4.5	4.72	4.97	4.46	1.29	3.33	4.2
AXIN2	5.63	1.79	2.73	0.74	1.94	1.91	-0.36	2.5	2.36
B2M	12.49	12.51	12.51	12.57	12.38	12.72	13.39	12.91	12.55
BAG1	6.1	6.15	5.48	5.47	5.45	5.89	5.67	5.86	5.96
BAK1	5.07	4.17	4.82	4.82	5.03	4.7	3.91	4.33	4.06
BATF2	4.05	1.35	3.12	2.99	2.43	2.36	2.59	1.35	2.17
BBC3	4.12	3.22	3.3	3.71	2.85	2.77	2.92	3.6	2.36
BCCIP	5.66	5.95	5.35	4.97	5.6	5.59	5.2	5.29	4.94
BCL2L14	4.16	-0.45	3.52	-2.73	1.2	0.44	-2.93	-3.17	-3.05
BCL6	2.6	4.45	4.15	3.54	5.07	4.95	4.79	4.39	5.35
BIRC3	3.11	2.73	4.5	1.81	3.78	3.53	5.2	3.77	1.26
BIRC5	4.91	4.08	4.04	4.5	4.55	4.74	1.32	3.67	4.86
BRCA2	1.79	1.19	2.06	0.97	2.54	1.01	-0.03	0.4	0.49
BRIP1	1.2	0.99	1.14	1	1.96	0.96	-0.83	0.2	0.48
BSG	9.34	8.8	8.86	9.92	8.89	8.81	9.23	9.94	9.24
BST2	5.86	7.92	7.05	7.69	6.96	7.13	7.5	8.17	6.97

BTG1	5.22	6.06	5.35	5.87	5.61	6.06	6.73	4.85	6.06
BTK	0.59	2.16	1.37	2.23	0.71	2.38	2.45	2.36	2.93
BTN3A2	4.32	4.38	4.7	5.2	4.63	4.41	5.63	4.97	4.86
BUB1B	3.47	2.8	3.24	2.99	4.06	2.98	0.38	1.84	2.54
C11orf24	5.01	5.05	5.28	6.19	5.15	4.95	4.3	5.23	4.76
C19orf48	6.32	5.41	5.33	5.5	5.47	5.5	4.48	5.88	5.32
C19orf66	4.08	4.19	4.46	4.75	4.42	4.15	4.47	5	4.83
C1orf112	2.53	2.91	2.41	2.7	2.94	2.65	1.13	1.81	2.42
C1orf54	2.92	4.16	3.37	3.6	2.76	3.78	5.08	4.34	5.68
C1QA	5.8	6.7	6.6	7.41	5.81	7.44	7.93	8.28	8.33
C1QB	5.96	6.87	7.44	7.5	6.68	7.6	8.07	8.36	9.12
C1S	5.96	7.18	6.54	5.96	6.43	7.25	6.83	9.76	6.54
C3AR1	1.88	3.32	2.82	2.59	2.05	3.22	3.81	3.71	4.6
CALCRL	1.69	3.21	3.22	1.08	3.08	2.91	4.97	1.74	3.77
CAMLG	4.82	5.39	4.25	4.62	4.25	4.61	5.43	5.33	5.63
CASP1	5.2	4.22	4.7	4.83	4.77	4.81	5	4.94	4.73
CASP8	4.37	4.05	4.59	3.57	4.63	4.16	4.26	4.13	2.93
CBX1	5.15	5.98	4.61	5.39	4.92	5.64	5.08	6.1	6.17
CCBL2	4.05	4.45	3.82	3.8	3.94	3.77	3.72	3.57	4.28
CCL18	3.7	1.67	4.52	3.73	4.07	6.38	2.45	0.72	-0.86
CCL19	1.09	4.11	2.62	3.55	1.12	4.7	0.93	2.76	-1.43
CCL2	4.11	5.23	5.59	5.16	4.6	5.94	6.94	7.9	6.66
CCL21	3.66	3	4.87	2.92	3.8	5.53	1.38	3.33	-9.97
CCL3	2.96	3.21	2.88	3.41	2.06	3.67	3.59	4.36	3.83
CCL4	2.98	3.14	3.98	3.28	2.85	3.81	4.37	4.41	3.23
CCL5	3.69	4.68	4.96	4.8	3.89	5.1	5.49	5.13	2.45
CCL8	-0.54	1.23	0.71	1.02	-0.29	1.14	0.9	1.64	0.11
CCNA2	4.45	3.16	4.21	3.35	4.56	3.6	1.22	2.55	3.46
CCNB1	6.24	5.46	5.41	5.6	5.82	5.63	3.15	5	5.69
CCNB2	5	4.02	4.78	4.21	5.31	4.53	1.34	3.96	4.34
CCNE2	2.07	2.38	2.07	1.5	2.63	1.99	0.14	0.61	2.46

CCNG2	3.81	5.78	3.82	3.8	3.89	4.24	5.14	3.81	3.96
CCNL2	5.55	5.5	5.3	5.54	5.9	5.8	5.27	5.87	6.06
CCR5	0.41	1.66	1.01	1.08	-0.01	1.54	2.42	1.96	1.22
CCR7	-0.06	1.07	1.14	0.6	0.36	1.35	0.67	0.07	-1.43
CCRL2	3.23	1.22	2.82	0.33	1.5	1.67	2.13	0.93	1.82
CCT5	7.88	7.68	7.42	7.67	7.9	8	6.82	7.38	7.24
CD14	4.62	5.36	5.3	5.73	4.83	5.82	6.24	7.27	7
CD163	2.8	4.34	3.62	4.29	3.16	4.85	5.22	5.71	6.27
CD19	-0.9	-1.45	0.13	-1.06	-0.83	0.75	-2.11	-1.6	-1.78
CD1A	-2.18	-2.31	-1.88	-3.05	-0.8	0.24	-3.82	-4.04	-5.01
CD1B	-2.78	-2.63	-2.61	-3.31	-2.83	-1.69	-3.82	-3.63	-9.97
CD1C	-0.73	0.22	0.09	-0.62	-0.46	0.74	0.07	0.06	-2.39
CD1D	-0.13	-0.2	-0.23	-0.22	-1.04	0.35	2.25	0.51	0.07
CD1E	-1.07	0.25	-0.66	-1.55	-1.18	0.1	-0.23	-1.21	-3.05
CD2	2.31	3.09	3.35	2.83	2.4	3.36	3.78	2.78	0.61
CD200	1.96	2.87	2.36	3.81	1.76	2.36	4.61	6.1	4.03
CD22	-0.48	1.76	0.82	2.19	-0.45	1.22	0.65	0.58	0.11
CD24	9.46	9.29	9.21	0.39	8.75	7.58	10.14	1.77	5.07
CD247	1.12	1.53	1.45	1.47	0.76	2.1	2.87	2.08	0.26
CD27	1.61	2.27	2.69	2.87	1.85	3.35	2.93	2.31	-0.38
CD274	0.31	0.6	1.16	0.74	1.52	1.98	1.24	0.47	0.96
CD33	0.26	1.92	1	1.8	0.29	2	2.61	2.95	3.4
CD34	3.36	4.64	4.29	3.54	3.8	3.72	6.57	3.73	4.26
CD36	1.42	4.29	2.52	2.76	2.18	3.04	6.27	2.45	1.74
CD37	2.69	4.46	4.6	4.1	3.67	4.74	4.97	4.94	5
CD38	0.56	0.42	1.74	1.47	1.38	2.77	0.81	2.11	2.07
CD3D	2.8	2.98	3.45	3.14	2.56	3.67	3.79	3.23	0.81
CD3E	2.81	3.36	3.96	3.62	3.17	3.89	4.3	3.45	0.89
CD3G	0.2	0.82	1.52	0.43	0.57	1.31	1.78	0	-1.69
CD4	3.3	4.51	4.31	4.31	3.6	4.92	5.21	4.91	5.1
CD40LG	-1.39	-0.77	-0.18	-1.94	-1.83	-0.81	0.06	-1.12	-3.63

CD44	7.25	7.59	7.48	8.53	8.35	7.53	5.56	6.88	7.73
CD48	2.65	3.92	3.82	3.32	2.57	4.15	4.61	3.91	2.9
CD5	0.99	0.86	1.77	0.71	0.47	1.26	1.35	0.54	-1.47
CD52	4.7	5.64	5.65	5.16	4.32	6.47	6.15	5.61	3.61
CD53	4	5.01	4.84	4.58	4.06	5.49	5.74	5.47	6.26
CD55	6	5.62	7.04	5.52	6.45	6.33	4.11	5.42	2.81
CD59	7.24	8.39	7.44	9.19	7.7	8.06	8.58	7.65	8.08
CD69	0.79	1.76	1.85	0.31	1.11	2.28	2.55	2.34	1.37
CD7	2.09	1.58	3.56	2.67	2.8	3.12	2.51	3.2	0.57
CD72	0.66	1.69	1.47	2	0.78	1.96	2.16	2.48	1.57
CD74	9.61	10.79	11.01	10.69	10.29	11.11	11.91	11.1	10.93
CD79A	2.2	2.17	3.81	2.28	2.33	4.27	0.86	1.5	-1.28
CD79B	0.66	1.81	1.28	2.55	0.18	2.37	2.88	1.66	0.04
CD83	2.22	3.18	2.37	3.92	2.35	3.49	3.06	2.91	3.67
CD84	-0.01	1.48	1.56	0.61	0.66	1.48	2.03	0.99	1.92
CD86	1.39	2.58	2.59	1.97	2.17	2.92	3.02	2.91	3.57
CD8A	1.36	2.28	2.47	2.61	1.38	2.65	3.53	2.38	0.39
CD8B	1.31	1.27	1.49	1.57	0.04	1.61	2.25	1.34	-0.73
CD9	9.21	8.92	7.82	7.2	8.67	9.17	7.71	8.28	8.49
CDC14B	2.71	1.64	2.93	2.4	3.09	2.43	2.97	2.33	3.53
CDC20	5.79	4.65	5.32	4.97	5.86	5.28	1.89	4.4	4.93
CDC45	3.7	2.65	3.21	3.61	3.66	3.56	0.55	2.18	3.17
CDC6	3.94	3.01	3.39	3.49	3.64	3.51	0.76	2.32	2.46
CDC7	1.93	2.04	1.93	2.48	2.47	2.06	0.78	1.18	2.98
CDCA4	3.8	3.49	3.46	4.11	4.26	4	1.9	2.95	3.08
CDH5	2.59	3.61	3.52	2.56	3.14	3.06	5.59	2.62	3.46
CDH6	-0.08	0.15	0.83	-1.28	0.93	-0.12	5.82	0.96	2.68
CDK1	5.36	4.79	4.97	4.2	5.57	4.93	2.41	3.75	4.43
CDK2	5.08	4.95	4.46	7.41	4.66	4.92	4.14	4.67	4.93
CDKN1A	6.16	5.64	5.84	6.19	6.28	6.21	6.79	6.61	6.41
CDKN1C	2.49	3.36	3.28	3.37	3.61	3.28	4.02	4.18	3.64

CDKN3	5.09	4.14	4.68	4.37	4.95	4.36	1.92	3.46	4.49
CEBPD	3.95	3.99	5.8	3.2	6.15	5.27	5.66	5.63	5.49
CECR1	2.76	4.23	4.18	4.16	3.38	4.61	4.94	3.86	4.14
CENPA	3.7	2.8	3.12	3.41	3.75	3.42	0.02	2.1	3.22
CENPE	1.6	1.41	1.99	1.08	2.75	1.11	-1.21	0.43	0.82
CENPJ	2.55	1.95	1.97	2.08	2.69	1.9	1.08	1.57	1.78
CENPN	4.68	3.36	4.2	4.4	4.8	4.16	2.07	3.32	4.07
CENPO	3.02	2.74	2.96	3.07	3.39	2.97	1.64	2.48	3.63
CENPW	5.48	3.91	3.97	4.02	4.51	4.69	1.85	3.72	3.13
CEP55	4.16	3.11	3.9	2.95	4.59	3.77	0.77	3.14	1.98
CEP57	4.23	4.1	4.25	4.05	4.41	4.25	4.15	3.93	4.21
CEP78	2.88	3.08	2.9	2.64	3.36	2.6	1.74	2.32	3.01
CHEK1	4.15	2.89	3.61	2.99	3.88	3.38	2.08	3.05	3.39
CIAPIN1	5.39	4.95	4.87	5.49	5.08	5.16	4.92	5.18	5.68
CIITA	1.32	2.08	3.25	2.05	2.59	2.56	2.8	2.41	2.07
CKAP2	4.36	3.79	4.15	3.76	4.69	3.58	2.8	3.15	4.19
CKLF	6.07	5.69	5.76	5.92	5.49	5.73	5.42	5.63	6.53
CLCN6	1.65	1.98	2.17	2.89	2.57	2.06	2.56	1.97	3.54
CLEC14A	2.05	3.27	2.98	2.15	2.63	3.02	5.44	2.21	3.23
CLEC3B	2.28	3.43	2.59	2.2	1.88	3.02	5.89	3.45	3.32
CLEC4A	1.13	1.74	1.21	1.06	0.95	2.26	2.3	2.06	2.19
CLIC2	1.11	2.51	2.31	1.51	2.09	3.23	3.3	3.08	1.95
CLIC5	3.43	0.26	1.87	-0.91	0.51	1.07	1.04	3.71	-1.78
CMPK2	1.96	2.63	3.13	1.34	2.97	2.55	2.44	2.46	2.49
CNRIP1	1.24	2.56	2.07	3.25	1.56	2.03	3.55	3.01	5.33
CNTNAP1	0.74	1.02	1.61	2.15	1.65	1.2	1.55	2.41	4.05
COL15A1	3.88	4.75	4.88	4.02	4.24	3.19	4.67	3.45	0.46
COL16A1	4.05	5.02	4.01	4.93	4.54	4.3	2.1	4.89	4.61
COL1A1	8.42	10.02	9.03	7.66	8.95	8.62	6.65	10.55	4.89
COL1A2	8.34	10.2	8.67	7.69	8.36	8.52	7.25	10.02	6.26
COL2A1	-3.5	-1	-4.61	-1.47	-2.63	-3.17	-6.51	-3.17	-1.99

COL3A1	8.17	9.68	8.43	7.09	8.17	8.47	6.65	9.87	5.31
COL6A1	6.23	7.91	7.13	7.02	7.19	6.43	6.31	9.12	7.01
COL6A3	5.77	7.21	6.31	5.19	6.12	6.16	4.1	6.07	1.66
COL8A1	2.89	4.37	3.75	0.83	2.97	4.25	4.54	4.74	2.38
COPS6	6.76	6.86	6.29	7.22	6.53	6.76	6.73	6.9	7.63
COQ2	3.75	3.12	3.18	3.03	3.3	3.31	2.23	3.12	3.5
CORO1A	4.88	4.9	4.8	5.01	4.11	5.41	5.48	5.82	5.81
CORO1C	5.6	5.1	5.62	6.07	6.08	5.71	5.78	5.75	6.2
COTL1	6.45	6.19	7.01	7.5	6.23	6.42	6.78	7.01	7.53
COX17	6.19	6.29	5.75	6.12	5.78	6.19	5.9	5.91	5.85
CPEB4	2.13	3.45	3.02	2.86	3.47	2.71	3.92	1.57	3.82
CPVL	3.66	4.19	4.76	5.47	3.99	4.75	5.98	5.64	6.13
CR1	-2.39	-2.31	-0.49	-1.73	-2.24	-0.39	-1.32	-0.83	-2.63
CR2	-1.86	-3.17	-0.05	-4.04	-2.13	-0.23	-5.01	-3.63	-9.97
CRABP1	-1.57	1.74	-0.8	-2.39	-1.45	-0.32	-4.61	-3.17	0.98
CRTAP	6.58	6.87	6.77	8.03	6.74	6.65	7.2	7.48	6.66
CSF1	2.61	3.99	4.1	3.85	4.08	3.76	4.45	5.31	5.03
CSF1R	2.74	4.07	3.66	3.69	3.03	4.08	4.99	5.26	5.84
CSF2	-1.49	-5.01	-1.55	-3.82	-0.72	-0.51	-5.01	-2.83	-9.97
CSF2RA	1.52	2.54	2.37	2.36	1.84	2.76	3.1	3.96	3.75
CSF2RB	1.18	1.57	2.35	0.92	1.63	2.09	1.91	1.33	0.43
CSF3	-0.45	-5.57	0.21	-5.57	1.38	-0.76	-5.01	-3.46	-2.11
CSF3R	1.49	3.24	2.5	1.89	1.61	3.5	3.08	3.12	3.92
CSNK2A1	5.48	5.65	5.78	5.52	6.11	5.48	5.35	4.86	5.56
CST3	8.96	8.79	9.59	9.12	9.07	8.51	8.73	9.19	11.16
CTGF	5.67	7.17	6.45	5.09	5.84	6.03	6.16	6.32	5.8
CTLA4	-0.13	-0.24	0.7	0.82	0.61	0.82	-0.51	-0.55	-1.94
CTNNA1	7.43	7.67	7.12	7.21	7.49	7.29	7.71	7.39	7.53
CTNNB1	7.53	7.46	6.89	8.09	7.17	7.24	6.77	6.77	7.46
CTNS	2.38	3.02	3.01	3.88	3.07	2.92	3.16	3.18	3.91
CTSC	6.75	5.94	7	6.81	7.18	7.9	7.13	6.73	5.91

CTSF	2.75	5.86	3.13	6.15	3.07	4.54	5.66	5.65	6.31
CTSL	5.24	6.41	5.9	7.81	5.66	6.67	7.63	7.42	7.1
CTSS	7.43	5.84	6.81	5.68	5.54	6.93	6.56	5.2	5.78
CXCL1	5.86	-0.89	5.65	1.43	5.92	4.43	1.92	3.05	0.74
CXCL10	3.7	4.65	3.99	3.99	3.72	4.61	4.67	4.11	3.58
CXCL11	2.45	2.35	2.08	1.06	1.91	1.5	1.86	0.99	0.8
CXCL13	1.63	2.75	3.76	2.66	2.86	4.34	1.11	1.9	-1.43
CXCL2	3.91	0.02	4.36	-0.51	3.68	3.87	2.4	2.36	2.19
CXCL8	4.72	0.59	4.96	2.18	5.14	4.73	1.34	2.11	4.05
CXCL9	3.04	4.02	4.27	4.23	3.59	4.36	4.33	2.65	0.58
CXCR4	4.27	5.87	5.41	4.61	4.31	5.68	7.21	5.33	5.77
CYBB	2.68	3.71	3.59	2.94	2.84	4.1	4.16	4.19	4.52
CYCS	7.5	6.31	6.9	6.66	7	6.65	5.88	6.18	7.14
CYP27A1	4.22	4.8	3.58	6.86	2.59	4.41	5.25	4.72	5.09
CYTH4	1.48	2.48	2.2	2.38	1.45	2.76	3.17	3.59	3.44
DAPK1	1.62	2.21	3.7	3.63	3.15	3.43	4.07	4.67	3.12
DBNDD1	3.74	4.48	3.7	5.26	3.73	3.66	3.4	3.07	4.68
DCBLD2	2.19	2.83	2.35	4.18	3.22	3.76	3.19	4.19	3.25
DCK	3.84	3.89	4.02	3.58	4.32	3.79	3.57	3.14	4.39
DCLRE1B	2.12	2.19	2.04	2.21	2.23	1.99	1.6	1.71	2.83
DDAH2	6.29	6.65	5.55	5.48	5	5.55	5.7	6.66	6.53
DDX58	2.15	3.79	2.73	1.91	3.19	2.74	3.43	2.74	2.54
DDX60	3.19	3.92	3.38	2.97	3.87	3.23	3.91	3.43	2.99
DEPDC1	2	1.48	2.25	1.61	3.05	1.91	-0.89	0.77	1.37
DHFR	4.53	4.14	4.33	4.15	4.33	3.63	3.4	3.59	4.75
DLEU1	3.58	2.59	3.03	3.62	3.28	2.92	3.19	2.07	3.19
DLEU2	1.64	1.51	2.13	1.58	2.67	1.65	0.99	1.29	1.66
DLGAP5	3.34	2.62	3.23	2.81	3.89	3.1	0.03	1.81	2.56
DNAL4	3.78	4.71	3.05	4.1	3.29	4.42	4.3	3.33	4.21
DNM1	1.89	2.52	2.71	1.82	3.46	2.25	2.91	2.67	4.82
DOCK2	1.25	2.5	2.17	2.25	1.62	2.66	3.12	2.94	2.84

DONSON	3.6	3.92	3.56	3.92	3.89	3.68	2.77	3.76	4.31
DPP7	6.61	6.76	6.23	7.71	6.09	6.77	7.03	7.1	7.16
DPYSL2	4.19	4.42	4.64	4.95	4.42	4.4	5.83	5.75	7.49
DSCC1	2.88	2.17	2.52	2.13	2.93	2.3	0.57	1.38	2.37
DSP	5.7	6.58	5.74	-0.03	7.38	6.21	3.36	4.67	-1.15
DUSP22	4.2	4.29	3.95	5.08	4.13	4.73	4.32	4.26	4.48
DUT	5.92	6.15	5.35	6.12	5.69	5.93	5.57	6.23	6.37
DVL1	5.17	4.64	4.77	5.06	5.38	4.97	4.32	4.79	4.95
DVL3	4.75	5.03	5.35	5.21	6	5.14	4.36	5.21	5.33
DYNLT1	7.17	7.53	6.89	6.07	6.94	7.27	7.03	6.95	7.86
E2F8	2.26	0.88	2.67	0.55	2.9	1.35	-0.97	0.91	0.61
EBF1	-0.76	1.27	1.18	0.06	0.91	-0.07	2.75	0.15	0.74
EBI3	0.06	1.13	1.12	1.13	0.14	1.16	2.31	2.07	2.64
EBNA1BP2	6.3	6.15	5.91	6.03	6.12	5.95	5.4	6.15	6.07
ECSCR	2.46	3.57	3.04	2.37	2.43	3.07	5.53	2.67	4.49
ECT2	4.92	4.25	5.02	3.13	5.59	4.46	2.28	3.33	3.54
EDNRB	1.06	1.32	2.02	7.15	1.24	1.23	5.35	0.9	5.77
EEF1E1	4.77	4.53	4.68	4.8	4.66	4.49	4.54	4.26	4.63
EFNA1	6.57	6.98	6	4.43	6.42	7	6.82	6.25	5.4
EFNA5	-0.54	2.13	1.93	1.86	3.23	2.53	2.12	4.06	0.14
EIF2AK1	6.23	6.51	6.52	5.9	6.85	6.17	5.55	5.96	6.22
EIF2B1	4.72	5.02	4.61	5.04	4.77	4.88	4.75	4.91	5.23
EIF4EBP1	6.34	5.93	6.3	6.96	6.82	6.34	5.84	6.54	6.1
EIF4G1	7.46	7.44	6.94	7.6	7.64	7.84	6.73	7.6	7.07
ELK1	4.53	4.44	4.36	4.75	4.33	4.23	3.91	4.6	4.54
ELK4	2.44	3.13	3.7	2.92	4.12	3.01	3.07	2.49	2.21
EMCN	1.14	2.92	2.37	1.58	1.62	1.76	5.01	1.65	2.48
EMP2	5.13	6.47	5.29	4.82	5.74	6	4.32	4.07	3.48
ENG	4.86	5.78	5.7	5.93	5.55	5.45	7.46	5.65	5.87
ENO1	9.84	9.13	8.89	10.33	9.54	10.4	10.94	10.22	10.26
EPHA2	4.88	2.03	5.15	3.47	5.94	4.28	4.01	5.04	2.23

EPHB1	-0.42	-0.78	-0.1	-1.21	-0.36	-0.64	-0.32	-1.51	2.53
EPHB2	5.83	1.39	4.13	0.31	3.46	2.56	0.06	3.95	3.19
EPHB3	5.63	4.24	3.49	2.47	4.55	3.67	0.52	3.74	3.32
EPHB4	5.63	5.37	5.19	4.56	5.51	5.27	4.19	4.5	3.78
EPO	-5.57	-3.46	-5.57	-5.01	-3.72	-4.61	-0.91	-4.29	-2.31
EPOR	0.83	1.78	1.14	2.98	1.22	1.99	2.69	1.84	2.52
EPS15	3.63	4.32	4.16	4.49	4.45	3.88	4.24	4.07	4.61
EPSTI1	4.8	3.32	4.4	2.4	3.9	3.44	3.79	3.77	2.69
ERCC6L	1.32	0.84	0.85	0.42	1.61	0.71	-2.31	-0.58	-0.25
ERLIN1	4.75	4.2	4.24	3.91	4.55	4.22	4.1	3.97	3.46
ESAM	3.55	4.33	4.45	3.16	3.92	4.13	6.56	3.24	5.32
ESPL1	2.52	1.77	2.63	2.39	3.48	2.04	0.52	1.1	1.69
ETV7	3.04	1.84	3.33	1.25	3.13	2.68	2.64	3.06	0.91
EVI2A	1.44	3.13	2.4	2.62	1.84	3.25	3.66	3.33	4.63
EVI2B	2.22	3.48	4.2	3.02	3.05	3.76	4.15	3.53	3.97
EXO1	2.72	2.5	2.45	2.9	3.08	2.53	-0.22	1.37	2.15
EXOC7	4.85	5.47	5.21	5.58	5.68	5.3	5.6	5.4	5.78
EXOSC8	5.22	4.4	4.77	4.47	4.68	4.39	4.23	4.42	5.13
EZH2	3.94	3.34	3.47	3.42	3.74	3.34	1.58	2.64	3.94
EZR	7.28	7.31	7.4	5.98	7.5	7.4	7.9	8.54	6.24
F10	1.14	0.13	0.93	-0.73	-0.04	0.26	1.54	-0.25	-0.58
F3	4.46	3.21	4.66	0.84	4.91	5.38	1.3	3	6.74
FABP3	2.29	3.32	3.16	3.66	2.25	3.3	6.75	4.06	4.65
FADS1	2.41	2.72	2.88	4.16	3.1	4.09	3.22	4.13	5
FADS2	3.31	5.34	3.96	4.32	3.9	5.37	4.32	5.57	7.74
FAM167A	-0.55	0.22	-0.04	-1.28	0.88	0.47	-1.28	2.84	3.17
FAM64A	3.31	2.33	2.39	3.55	3.07	2.84	0.49	1.67	4.55
FAM89B	4.97	5.63	5.35	5.56	5.54	5.59	5.14	5.74	5.55
FANCI	4.08	3.83	3.95	4.13	4.6	3.76	2	3.26	3.56
FARSA	6.11	5.61	5.34	6.15	5.61	5.44	5.05	5.62	5.81
FARSB	4.77	4.34	4.15	4.38	4.46	4.12	3.44	4	4.72

FAS	2.97	2.98	3.19	2.38	3.2	3.49	4.32	4.35	3.59
FASLG	-1.55	-1.06	-0.83	-0.89	-1.57	-0.76	0.18	-0.73	-3.63
FBXO41	1.75	1.63	1.54	1.52	2.08	1.37	0.14	1.27	2.07
FBXO5	2.87	2.62	2.76	2.37	3.13	2.57	1.31	2.28	3.43
FCER1G	5.31	6.45	7.03	6.43	6.29	6.74	6.82	7.09	7.69
FCER2	-3.17	-3.17	-1.66	-2.39	-3.63	-1.83	-3.05	-2.63	-3.31
FCGR1A	2.35	4.21	2.95	3.9	2.26	4.22	4.71	5.55	6.23
FCGR2A	3.92	4.65	4.92	5.74	4.27	5.11	5.43	5.15	5.6
FCGR2B	1.45	3.43	2.24	3.12	1.52	3.96	3.37	3.92	3.49
FCGR3A	3.78	5.49	4.43	4.9	3.62	5.56	6.35	6.21	7.08
FCGRT	7.24	6.79	7.04	6.75	6.05	6.7	7.61	7.19	7.34
FDPS	7.52	7.2	6.59	7.18	6.76	7.37	6.68	7	7.14
FEN1	4.87	4.48	4.25	5.01	4.68	4.52	3.1	4.39	4.46
FGD1	0.83	2.62	1.09	3.9	2.29	1.95	1.03	2.83	3.01
FGL2	1.92	3	3.26	2.35	2.39	2.88	4.6	3.17	3.14
FGR	1.47	2.05	2.11	1.77	1.73	2.98	3.23	2.26	2.85
FLI1	1.6	3.06	2.87	2.29	2.11	2.82	4.42	3.06	3.34
FLNC	1.83	0.35	2.48	-0.76	1.98	0.48	-0.12	5.4	3.87
FLT3	-3.46	-0.25	-2.47	-2.83	-3.05	-2.05	-1.21	-0.49	-3.31
FLT3LG	2.21	2.89	2.99	2.64	2.84	2.96	3.1	3.26	3.56
FMNL1	2.79	3.97	4	3.79	3.73	4.33	4.17	5.02	3.89
FN1	7.78	10.25	8.06	9.29	7.97	9.25	8.74	9.82	9.2
FNBP1	2.99	4.18	3.7	3.95	3.84	3.65	3.97	4.15	4.23
FOS	6.87	6.31	7.1	5.94	7.55	6.66	7.22	7.18	7.19
FOSL1	3.27	0.44	3.85	3.53	5.46	3.12	1.6	1.87	2.59
FOXM1	4.92	4.07	5.02	4.96	5.69	4.66	1.9	3.96	4.68
FOXP3	0.67	1.33	1.74	0.89	1.48	1.29	-0.04	-0.03	-1.06
FPR1	1.21	1.73	1.64	1.13	0.94	2.63	2.7	3.07	5
FPR3	2.23	3.52	3.16	2.5	2.26	3.46	3.96	3.17	2.5
FUT3	6.33	-0.23	4.94	-4.04	4.35	3.65	-0.6	-2.83	-3.17
FYB	1.15	2.65	3.45	2.74	2.89	2.82	3.49	2.46	3.21

FYCO1	2.87	3.75	3.23	3.74	3.39	2.93	3	3.2	2.36
GABARAPL1	3.91	4.87	4.2	4.97	4.7	5.43	5.92	5.92	5.6
GABBR1	2.31	3.48	2.15	3.63	3.15	2.91	2.56	2.92	5.26
GABRQ	-6.51	-5.57	-5.57	-4.29	-1.55	-3.31	-1.64	-2.83	0.06
GAS2	0.06	-1.73	-0.78	-1.35	-1.64	-1.6	-0.49	-1	-0.32
GATM	2.62	3.54	4.89	2.7	4.2	2.73	7.28	2.22	7.29
GBP1	4.07	4.63	4.73	4.35	4.97	4.86	5.25	4.82	5.19
GBP4	2.59	3.31	3.87	3.29	3.66	3.42	4.93	3.59	2.46
GBP6	-5.57	-2.93	-3.31	-4.29	3.4	0.69	-4.04	-4.04	-3.05
GBP7	-6.51	-5.01	-5.01	-4.61	-5.01	-5.57	-3.17	-6.51	-5.01
GGH	6.59	4.55	4.85	5.26	5.56	5.01	3.72	4.31	5.43
GIMAP1	-0.47	0.83	0.58	0.4	-0.32	0.56	2.55	1.23	1.25
GIMAP4	2.8	4.26	3.86	3.55	2.95	3.9	6.01	4.24	4.44
GIMAP5	1.88	2.69	2.08	3.07	1.68	3.18	5.27	3.03	3.01
GIMAP6	1.57	2.87	2.54	2.03	1.8	2.47	4.8	2.81	3.22
GIMAP7	2.05	3.34	3.09	2.6	2.14	3.12	5.34	3.23	3.1
GIMAP8	0.47	1.75	1.38	1	0.9	1.53	3.93	1.69	1.77
GINS1	3.33	2.6	2.71	2.49	3.19	2.83	0.81	2	3.3
GINS2	3.75	3.06	2.71	2.9	2.87	2.84	1.31	2.14	3.29
GLRX	5.78	5.33	4.8	4.28	4.21	5.21	7.32	4.2	6
GLRX3	6.25	5.75	5.69	5.4	6.18	6.24	5.38	5.64	5.54
GLS	5	4.4	4.69	5.19	5.43	4.87	6.36	6.05	3.97
GMFG	4.02	4.86	4.25	4.84	3.5	5.22	6.37	5.75	5.84
GNG11	1.92	3.13	2.53	3.23	2.04	2.59	5.59	3.52	3.75
GNLY	1.91	2.06	2.89	2.3	2.67	3.48	3.77	3.81	1.94
GNPTAB	3.9	4.31	3.97	5.04	4.2	4.21	4.09	3.76	3.98
GP1BA	-1.92	-1.6	-0.98	-1.18	-0.78	-1.25	-1.09	-1.09	-2.24
GP1BB	-2.31	-3.28	-0.51	-1.51	-0.43	-1.43	-9.97	-0.47	1.39
GP5	-5.01	-4.61	-4.04	-5.57	-4.61	-4.61	-3.82	-5.01	-5.57
GP9	-5.01	-9.97	-5.57	-9.97	-9.97	-5.57	-2.93	-9.97	-4.61
GPLD1	-2.47	-1.64	-1.28	-1.94	-1.18	-0.86	-1.43	-1.09	-0.32

GPNMB	4.49	6.64	5.6	9.49	6.21	7.42	6.17	6.72	5.85
GPR171	-0.07	0.15	1.17	-0.43	0.23	1.23	1.03	-0.18	-2.24
GPR18	-1.78	-0.91	-0.51	-0.81	-1.2	-0.91	-0.58	-1.15	-2.55
GPSM2	5.18	3.53	3.92	3.19	4.55	3.6	1.46	1.96	4.81
GRB2	5.87	6.37	5.9	6	5.76	6.02	5.82	5.96	6.2
GSN	6.96	7.65	7.26	8.26	7.58	7.61	8.11	7.74	7.6
GSTCD	2.06	2.63	2.41	1.31	2.45	1.86	1.57	0.74	2.34
GTSE1	3.18	2.17	2.92	3.37	3.54	2.65	0.19	1.77	2.77
GUSBP11	1.68	1.86	1.8	1.53	2.06	2.17	2.62	1.97	2.33
GVINP1	-1.88	-1.12	-0.36	-1.69	-1.12	-0.97	0.4	-1.83	-2.83
GYPA	-9.97	-9.97	-9.97	-9.97	-9.97	-9.97	-2.39	-6.51	-6.51
GZMA	2.41	2.68	2.9	2.55	1.94	3.45	4.24	3.65	0.96
GZMB	3.34	1.08	2.98	2.6	2.72	3.39	2.78	2.47	-0.04
GZMH	0.67	1.05	1.62	1.56	0.46	2.1	3.08	2.12	0.06
GZMK	-0.1	1.87	1.54	1.44	-0.23	1.91	3.39	2.03	-0.58
H2AFZ	8.39	8.1	7.32	8.92	7.69	8.01	7.03	7.46	8.16
HADH	6.03	5.68	5.09	4.91	4.91	4.99	4.9	4.39	5.59
HADHB	6	6.5	5.59	6.58	5.87	6.07	6.82	5.99	6.73
HAPLN1	-1.28	-1.47	-1.2	-3.63	-0.58	-1.99	1.7	-2.73	2.26
HAS2	1.06	0.27	0.18	1.95	0.6	0.64	-2.24	-0.15	2.3
HAUS1	4.41	4.81	3.76	4.45	3.61	4.24	4.02	4.51	5.19
HCK	2.55	3.26	3.11	3	2.7	4.22	3.78	3.8	4.14
HCLS1	3.36	4.46	3.89	4.23	3.47	5.2	5.56	5.16	5.5
HCP5	4.85	4.05	4.73	3.22	5.13	4.47	5.07	4.54	3.26
HDAC5	4.13	4.84	3.91	5.03	4.42	4.47	4.66	4.36	4.9
HECA	3.12	3.47	3.27	2.46	4.06	3.29	3.13	2.59	2.77
HELZ2	4.42	4.04	5.02	4.01	5.63	3.83	3.46	3.81	3.09
HERC5	0.26	2.14	1.58	2.78	2.06	1.98	2.25	1.78	2.91
HERC6	2.29	2.84	2.97	2.05	3.5	2.68	2.65	2.21	2.87
HIF3A	-1.23	-2.93	0.62	-2.73	0.3	0.37	0.38	-2.31	2.04
HJURP	3.39	2.53	3.23	3.14	3.78	3.07	0.31	2.17	2.93

HLA-A	9.97	9.35	9.62	10.65	9.57	9.97	10.86	10.22	9.95
HLA-B	10.27	10.01	10.04	10.35	10.08	10.22	11.27	10.66	9.97
HLA-C	9.5	9.37	9.51	9.59	9.55	9.47	10.16	9.61	9.22
HLA-DMA	5.15	6.17	6.38	6.32	5.23	6.66	7.16	6.65	6.52
HLA-DMB	4.59	5.6	5.6	5.2	4.43	6.19	6.53	5.8	6.59
HLA-DOA	1.49	3.22	3.01	2.96	1.62	3.7	4.58	3.3	3.93
HLA-DOB	-0.01	1.2	0.86	0.67	1.06	2.54	1.53	0.47	0.33
HLA-DPA1	6.07	7.72	7.61	7.46	6.36	7.88	8.95	7.65	8.03
HLA-DPB1	5.99	7.58	7.12	7.52	6.09	7.81	8.74	7.94	7.61
HLA-DPB2	-1.94	-0.1	-0.95	-0.47	-1.91	0.11	0.94	0.09	-1.25
HLA-DQA1	4.51	6.23	5.61	5.71	4.63	6.52	7.19	6.06	5.49
HLA-DQA2	1.03	3.27	2.43	2.62	2.08	3.46	3.99	3.03	1.98
HLA-DQB1	4.85	6.33	5.95	5.57	5.39	6.34	7.59	6.46	5.72
HLA-DQB2	0.84	2.89	2.37	2.23	2.09	3.63	3.57	1.96	1.9
HLA-DRA	8.09	9.36	9.2	8.87	8.29	9.86	10.41	9.46	9.75
HLA-DRB1	6.64	7.97	7.53	7.59	6.73	8.23	9.07	8.38	8.04
HLA-DRB5	4.04	5.68	4.87	5.37	3.77	5.92	6.86	6.05	5.08
HLA-DRB6	2.47	3.57	1.45	3.56	0.51	4.13	4.69	4.32	2.15
HLA-E	8.29	7.93	8.36	8.71	8.37	8.4	9.41	8.81	8.15
HLA-F	7.07	6.17	6.83	6.9	6.61	6.71	7.64	7	5.6
HLA-G	-0.76	-1.25	-0.17	-0.09	-0.49	0.56	4	-0.38	-1
HLA-J	0.04	-0.86	-0.64	-1.21	-1.07	-0.23	2.2	-0.38	-0.47
HMGB1	9.07	8.37	8.25	8.26	8.18	8.11	7.82	7.82	8.54
HMGCS1	5.36	4.82	4.79	3.81	5.56	5.07	4.12	3.99	4.9
HMGN2	8.81	9.07	8.28	8.7	8.51	8.71	8.46	8.95	9.35
HMHA1	4.76	4.35	3.91	4.46	3.84	4.52	4.28	4.77	4.05
HMMR	3.69	2.7	3.49	2.39	3.77	2.73	0.41	2.13	2.19
HN1L	5.75	6.51	5.56	5.31	5.65	5.88	5.74	5.45	4.54
HNRNPA2B1	9.28	9.21	8.77	9.11	9.13	8.95	8.45	8.99	9.25
HNRNPR	6.27	6.44	6.08	6.2	6.37	6.13	5.61	6.14	6.44
HRAS	5.51	5.72	4.77	5.98	5.68	5.89	5.53	6.16	6.31

HSPA4L	1.38	1.17	1.6	1.25	3.16	1.78	2.92	0.67	2.36
HSPB11	4.19	4.97	4.21	4.75	4.43	4.71	4.45	5.04	4.91
HSPG2	5.5	5.64	6.47	5.87	6.63	5.73	6.83	6.07	4.74
HYLS1	1.69	2.75	1.59	2.11	1.87	2.05	1.39	2.05	1.45
ICAM1	4.14	4.43	5.02	6.29	4.8	6.21	5.71	6.36	4.03
ID2	6.26	6.06	6.06	4.59	5.23	5.12	7.19	6.61	7.84
ID3	6.16	5.3	5.22	4.05	5.53	5.6	6.79	6.1	8.59
IDI1	5.37	5.12	4.74	4.66	5.12	5.09	4.1	3.97	4.85
IDO1	2.29	1.98	3.01	2.2	2.89	4.06	4.6	1.79	-0.55
IFI16	4.54	5.67	5.34	7.83	6.65	6.73	6.14	6.99	6.38
IFI27	9.95	8.41	9.31	8.08	9.66	9.15	8.31	9.45	7.03
IFI30	7.12	7.65	7.97	7.57	7.17	8.19	7.41	7.67	7.39
IFI35	5.33	5.43	4.87	5.7	4.92	5.26	5.43	6.04	5.42
IFI44	3.08	4.65	4.39	3.97	5.03	4.51	4.66	4.96	5.35
IFI44L	1.03	3.49	3.08	2.09	3.38	2.71	3.9	2.33	3.93
IFI6	6.84	8.22	8.14	8.1	8.32	7.55	6.84	7.69	8.17
IFIH1	3.11	3.77	3.47	3.2	4.04	3.33	3.61	3.16	2.79
IFIT1	2.09	4.87	3.42	3.17	3.96	3.67	5.2	4.74	4.56
IFIT2	1.41	3.39	2.21	2.72	2.14	2.56	3.98	3.34	3.31
IFIT3	3.22	4.39	3.98	4.22	4.24	4.04	4.93	4.42	4.71
IFIT5	2.35	3.1	2.6	2.59	2.88	2.82	3.49	2.56	3.03
IFITM1	9.2	8.41	8.11	7.48	7.86	7.82	7.62	8.51	6.19
IFITM3	9.6	9.64	10.02	9.63	9.48	9.48	9.94	11.14	9.31
IFNB1	-9.97	-5.57	-9.97	-9.97	-9.97	-9.97	-9.97	-5.01	-9.97
IFNG	-2.39	-2.83	-1.81	-1.6	-2.31	-1.32	-1.6	-1.94	-9.97
IFRD1	4.64	4.09	4.1	4.55	4.78	4.43	4.2	4.25	5.72
IFRD2	6.28	5.36	5.22	5.77	5.28	5.45	4.53	5.38	5.25
IGBP1	6.23	5.75	5.39	5.57	5.48	5.5	6.24	5.94	5.53
IGF2R	4.8	4.56	4.71	5.29	5.39	4.92	4.89	4.46	3.68
IGFBP2	7.42	7.38	6.68	5.77	6.75	8.06	4.27	7.66	9.27
IGFBP3	5.85	6.06	6.62	5.88	6.33	7.19	10.17	6.26	6.93

IGFBP4	7.47	8.63	7.45	6.6	7.35	7.76	9.1	8.67	5.99
IGFBP5	5.47	6.87	6.22	4.74	5.55	6	7.33	6.46	7.68
IGHA1	8.23	8.54	9.27	6.73	8.05	11.04	7.09	7.29	1.49
IGHD	-1.35	0.61	1.15	-0.69	-0.34	3.89	0.72	-0.17	-3.31
IGHG1	9.42	9.25	11.19	9.99	9.99	12.31	7.83	8.87	2.8
IGHG3	6.24	6.29	7.58	6.85	6.35	9.45	5.22	6.05	-0.6
IGHM	5.73	6.19	7.32	5.28	5.22	8.14	5.02	5.16	0.36
IGHV1-69	1.21	1.03	1.75	0.79	-0.35	4.34	-0.1	0.1	-9.97
IGKC	11	11.07	12.54	10.48	11.33	13.41	9.24	9.76	4.09
IGKV1-5	5.35	5.48	6.41	4.37	4.75	7.93	3.8	4.25	-0.94
IGKV1D-13	0.39	0.99	1.11	-0.76	-0.59	2.87	-0.39	-1.47	-9.97
IGKV1OR2-108	-9.97	-9.97	-2.93	-9.97	-2.55	-1.94	-9.97	-9.97	-9.97
IGLC1	7.48	7.72	9.5	7.13	8.49	10.23	6.21	6.93	1.07
IGLC2	8.96	9.06	10.83	8.59	9.48	11.64	7.46	8.25	2.47
IGLL5	4.21	4.62	5.52	4.18	4.31	6.97	3.25	3.18	-1.99
IGLV2-14	5.53	5.9	6.71	4.87	5.01	8.23	4.58	3.88	-0.43
IGLV3-25	3.96	4.45	5.52	4.23	4.56	7.32	3.07	3.23	-2.63
IGLV4-3	-9.97	-9.97	-3.72	-9.97	-9.97	-1.78	-9.97	-9.97	-9.97
IGSF6	1.43	2.33	2.47	1.79	1.6	2.77	2.94	2.08	3.14
IKZF1	0.47	1.69	2.2	1.18	1.27	1.92	2.38	1.95	1.57
IL10RA	1.86	2.83	3.03	2.82	2.15	3.33	3.96	3.55	3.28
IL11	0.04	-0.6	0.33	-1.83	0.62	-0.03	-4.29	0.33	-0.62
IL11RA	1.97	2.71	1.39	3.35	1.71	2.55	2.31	3.28	4.01
IL12B	-5.57	-3.82	-4.61	-5.01	-5.01	-3.63	-4.04	-5.01	-9.97
IL13	-4.29	-5.57	-4.29	-9.97	-5.01	-4.29	-5.01	-5.01	-3.46
IL13RA1	5.51	5.95	5.47	4.66	5.59	5.38	5.23	5.12	4.85
IL13RA2	-1.18	-1.83	-1.3	-0.1	-0.38	-0.89	-0.15	-1.55	4.28
IL15RA	4.18	3.12	4.66	2.1	4.41	4.05	4.05	4.47	1.79
IL18	4.61	3.31	4.75	2.29	5.15	4.35	4.48	6.83	4.25
IL18BP	2.91	3.18	4.05	3.81	3.43	3.83	4.71	4.61	4.01
IL1A	-0.61	-3.82	-1.43	-4.61	1.84	-0.58	-4.61	-3.17	-0.27

IL1B	3.12	1.46	3	0.55	3.37	2.52	1.83	2.44	3.1
IL1R1	2.74	4.09	4.18	2.26	4.09	4.76	4.15	5.32	1.77
IL1R2	2.36	-0.56	2.64	0.03	3.24	1.75	1.8	0.23	0.78
IL2	-9.97	-4.29	-4.61	-9.97	-5.57	-4.04	-3.82	-5.57	-9.97
IL2RA	0.41	0.14	1.49	0.42	1.06	1.55	0.16	0.9	-0.32
IL2RB	1.55	1.95	3.09	2.06	2.52	2.45	2.81	2.33	-0.39
IL2RG	6.02	4.07	6.23	4.09	4.69	4.77	4.49	4.07	2.04
IL3RA	2.12	2.32	2.07	1.69	1.41	2.24	3.77	2.16	1.55
IL4R	4.92	4.27	4.9	3.47	5.3	4.98	4.87	4.62	3.31
IL5	-4.61	-9.97	-4.29	-9.97	-5.01	-9.97	-5.01	-9.97	-4.61
IL5RA	-4.29	-3.46	-3.05	-4.29	-3.72	-1.43	-3.82	-3.63	-6.51
IL6	1.3	0.46	1.88	0.41	1.78	2.82	1.12	2.89	1.54
IL6R	1.05	1.61	2.31	3.22	2.68	2.94	3.41	1.72	1.94
IL6ST	3.1	6.09	4.52	4.37	4.68	4.25	5.73	5.34	5.21
IL7	2.05	-0.64	1.8	-0.53	1.41	0.99	1.55	1.21	0.28
IL7R	2.18	2.12	2.72	0.75	3	3.42	2.62	0.97	-0.1
IL9R	-1.66	-0.66	-0.86	-2.31	-1.07	-0.69	-1.47	-1.73	-3.17
IMP4	6.4	5.85	5.84	6.41	6	6.1	5.7	6	6.18
INPP5D	4.31	2.8	3.68	2.38	2.96	2.94	3.9	3.75	3.95
INSIG1	5.25	4.66	4.56	4.11	4.78	4.48	3.68	4.37	4.4
IPO4	4.94	4.91	4.15	5.22	4.77	4.58	4.02	4.47	3.99
IRF1	5.68	5.2	5.95	4.82	5.64	5.39	5.53	5.27	4.7
IRF3	5.98	5.51	5.53	5.74	5.62	5.96	5.36	5.93	5.65
IRF7	4.02	4.9	4.64	4.44	4.8	4.74	4.24	5.2	3.98
IRF8	4.32	2.67	4.33	2.09	2.69	2.89	3.42	2.94	2.99
IRF9	5.81	6.59	6.07	5.9	6.23	6.26	6.17	6.26	6.32
IRGM	-9.97	-5.01	-4.61	-6.51	-4.61	-5.57	-4.61	-4.61	-4.04
IRS1	2.42	3.16	3.06	1.64	3.43	2.23	1.28	0.53	0.96
ISG15	6.07	6.78	6.53	5.99	6.78	6.69	5.94	6.69	6.63
ISG20	4.62	4.74	5.19	5.03	4.94	5.48	4.27	4.78	3.82
ITGA1	3.08	2.94	3.73	1.84	3.27	2.6	5.03	2.04	2.3

ITGA2	3.79	2.71	3.54	1.08	4.29	3.71	2.6	0.65	1.57
ITGA2B	-2.93	-1.21	-2.08	-2.05	-1.09	-0.62	-1.03	-0.53	-0.17
ITGA3	5.21	4.87	5.85	6.21	6.8	6.49	6.55	7.68	4.74
ITGA4	1.26	1.74	2.32	1.59	1.78	2.02	3.13	1.4	1.31
ITGA5	4.43	5.27	5.26	4.47	5.72	5.17	6.18	5.66	5.11
ITGA6	6.55	4.53	5.99	4.53	6.93	4.79	5.64	3.8	4.23
ITGA9	1.54	1.48	1.49	1.87	0.88	1.4	2.25	0.61	0.2
ITGAL	1.36	3.05	3.05	2.31	2.25	2.86	3.37	2.5	1.94
ITGAM	0.67	2.4	1.6	1.61	1.25	2.46	2.66	3.01	2.87
ITGB2	4.08	5.21	4.77	5.17	3.96	5.88	6	6.44	6.31
ITGB3	0.26	0.71	1.2	3.73	1.23	0.31	2.64	0.28	1.18
ITGB5	6.03	7.04	5.94	6.65	6.4	6.41	6.28	6.95	5.62
ITGB8	1.58	1.66	2.85	1.36	4.15	2.71	4.12	1.14	5.04
ITK	-0.48	0.18	0.77	0.35	0.17	0.76	1.13	-0.06	-0.78
ITPKB	2.45	3.66	2.85	5.27	3.3	3.41	3.28	2.9	5.24
JAG1	4.31	4.01	4.8	3.19	6.08	4.27	5.56	2.94	4.57
JAK1	5.23	5.71	5.6	5.27	5.8	5.21	6.17	5.33	5.22
JAK2	1.77	2.36	2.85	1.59	2.64	1.83	2.3	2	1.96
JAK3	1.22	1.25	2.35	2.03	2.13	2.25	2.54	2.18	1.1
JAM2	0.41	2.45	1.77	0.52	1.14	1.58	2.76	1.53	5.76
JUN	6.4	5.84	5.98	4.58	6.08	5.82	6.69	6.22	6.68
JUNB	6.84	6.25	7.24	5.68	7.61	6.8	6.82	6.93	6.31
JUP	7.73	7.47	8.01	4.21	9.51	7.66	6.3	5.62	3.47
KDR	1.71	3.07	2.43	1.96	2.08	2.37	5.54	4.87	2.53
KIAA1109	3.31	4.19	3.59	3.4	4.09	3.37	4.06	3.41	3.62
KIF11	3.42	3.15	3.49	2.76	4.19	3.03	0.84	2.28	2.36
KIF14	1.07	0.65	1.24	0.94	2.26	0.95	-1.99	0	0.39
KIF15	2.93	1.98	1.93	1.89	2.47	1.84	-0.76	1.38	2.9
KIF18A	1.76	1.33	2.01	1.28	2.91	1.57	-0.43	1.14	1.29
KIF23	3.6	3.49	3.5	3.86	4.35	3.54	0.9	3.4	3.04
KIF2C	4.05	3.3	3.7	3.82	4.35	3.66	0.61	2.27	3.59

KIF4A	3.31	2.78	2.73	2.95	3.42	2.82	0.18	1.93	2.78
KIT	0.13	2.05	1	2.19	0.84	1.97	1.4	-1.51	1.21
KITLG	2.71	3.98	3.95	0.46	4.05	4.23	3.08	1.95	3
KLHL5	2.5	4.12	3.65	3.45	4.35	4.48	4.37	3.89	5.07
KLRC3	-4.04	-3.63	-2.51	-3.17	-3.31	-3.46	-3.17	-3.31	-0.45
KLRK1	-0.06	0.95	0.57	1.36	0.08	1.32	2.23	1.37	0.02
KRR1	4.69	5.08	4.81	4.51	5.2	4.92	4.26	4.42	4.18
LAG3	0.66	0.88	1.76	2.06	1.19	1.81	1.48	2	-0.04
LAIR1	2.69	4	3.84	3.57	2.99	4.1	4.55	4.72	4.97
LAMA4	3.92	4.42	4.43	5.48	4.2	3.56	5.27	5.32	4.62
LAMB1	5.87	5.73	5.68	6.12	5.88	5.41	6.04	6.92	5.03
LAMP3	1.51	1.2	2.7	0.61	3.49	4.48	0.96	1.46	-0.71
LAPTM5	5.55	6.8	6.48	6.22	5.82	6.94	7.3	7.24	8.02
LAX1	-1.21	-0.62	0.26	-1.47	-0.76	0.6	-0.45	-1.78	-3.46
LCK	2.41	1.93	2.96	1.99	2.29	2.66	2.88	2.01	-0.41
LCP1	4.07	5.5	5.1	4.76	4.59	5.67	5.54	4.72	4.43
LCP2	3	3.71	4.12	4.28	3.43	4.05	4.75	3.76	4.32
LDB1	4.37	5.35	4.67	5.2	5.08	5.08	4.81	4.68	4.48
LDB2	1.55	2.82	2.46	1.51	2.02	2.39	5.38	2.3	2.97
LDLR	5.4	4.13	5.3	3.39	6.01	4.92	1.8	3.96	3.13
LGALS3BP	8.89	8.41	9.19	9.59	9.22	8.97	8.06	9.45	8.03
LHFPL2	3.4	4.08	3.91	4.97	3.82	3.52	3.66	3.99	4.33
LILRA4	-2.88	0.09	-1.71	-1.15	-2.55	-0.47	-0.03	-1.47	1.05
LILRA6	-0.1	0.29	0.51	0.81	0.28	1.36	0.99	1.19	0.9
LILRB1	0.62	1.52	1.7	1.15	1.03	1.94	2.69	2.2	2.7
LILRB2	1.32	1.72	2.05	2.16	1.33	2.38	2.9	2.92	1.5
LILRB3	0.67	0.76	1.73	1.3	1.13	2.16	2.04	2.25	1.7
LILRB4	2.65	3.78	3.21	3.52	2.47	4.12	3.72	4.13	5.1
LIPA	4.84	5.53	5.37	5.53	5.31	5.7	6.49	4.93	5.77
LMNB1	5.56	4.92	4.75	4.49	4.99	4.69	2.97	4.08	5.02
LMNB2	5.48	4.21	4.83	4.85	5.56	4.74	3.12	4.63	4.53

LOXL2	4.23	4.49	4.4	3.54	4.57	3.78	5.04	4.83	4.12
LPIN1	3.15	2.78	3.36	3.55	3.8	3.01	2.17	2.74	3.27
LPXN	2.86	3.59	3.26	4.1	2.74	3.66	3.86	3.41	3.77
LRIG2	1.6	1.95	2.05	1.6	2.74	1.74	1.69	1.29	1.73
LRMP	0.51	1.21	1.42	0.55	0.77	2.06	3.58	1.4	2.11
LRP1	5.5	5.5	5.46	5.35	5.89	5.61	5.52	7.44	7.04
LRP8	2.76	1.33	2.24	2.92	3.67	2.54	-0.36	1.26	2.86
LRRC17	-0.08	2.89	0.13	-0.81	0.51	1.02	2.24	0.84	3.53
LRRC32	3.79	4.37	3.96	3.16	3.21	3.39	5.88	4.08	3.29
LRRC40	2.36	2.86	2.75	2.54	3.13	2.8	3.14	2.22	3.51
LSM3	3.73	3.99	3.86	3.49	3.6	3.34	3.09	3.98	4.25
LSM4	7.53	7.08	6.67	7.06	6.67	7	5.97	7.14	7.31
LSS	4.43	4.12	3.74	4.25	4.21	4.13	3.14	4.46	4.75
LST1	3.39	4.33	4.25	4.47	3.36	4.81	5.38	5.37	5.55
LTB	2.85	2.71	3.88	2.85	2.85	3.83	3.25	3.93	0.44
LUM	7.85	9.3	8.23	6.2	7.96	8.82	4.8	8.7	4.25
LY86	1	3.16	2.41	2.4	1.29	2.88	3.87	3.84	4.92
LY9	-0.9	0.2	0.76	0.03	-0.15	1.32	0.71	0.15	-1.94
LYAR	4.74	3.54	4.24	4.34	4.31	3.89	3.18	3.48	3.54
LYN	4.38	3.25	4.95	2.92	4.65	4.94	4.01	3.77	4.15
LYZ	7.96	6.44	9.83	6.08	7.17	7.91	7.35	5.75	5.16
MAD2L1	5.2	3.98	4.44	4.03	4.85	4.24	2.21	3.15	4.53
MAF	1.55	2.59	3.35	3.38	3.83	3.11	5.06	4.37	3.63
MAFB	1.95	4.44	3.6	3.51	4.59	4.06	3.94	4.21	4.55
MAGOHB	4.78	5	4.76	4.69	4.82	4.83	4.05	4.91	4.76
MAN1A1	3.73	4.25	4.38	1.7	4.16	3.51	4.47	3.21	2.3
MAP2K1	4.93	4.94	4.61	5.01	5.15	5.33	5.26	4.95	5.61
MAP3K10	2.84	2.63	2.88	3.34	3.68	2.95	2.53	2.86	3.71
MAP3K8	2.8	2.97	3.22	1.4	3.52	3.42	2.63	4.25	2.59
MAPK3	6.7	6.5	6.88	6.05	6.35	5.8	6.53	6.02	6.44
MAPK8	4.19	4.51	4.15	3.25	4.38	4.06	3.98	3.09	3.52

MAPRE1	6.38	6.34	5.87	6.3	6.24	6.16	5.83	5.93	6.73
MARCKSL1	7.93	8.1	7.62	8.13	7.07	6.89	5.72	6.28	8.45
MARVELD2	3.38	3.52	3.57	-2.39	3.68	3	1.26	0.84	-0.71
MATN1	-6.32	-4.61	-5.57	-2.73	-5.01	-4.29	-3.46	-3.17	-2.39
MBP	3.39	2.99	3.78	7.23	3.79	3.65	3.93	4.22	8.11
MCM10	2.03	1.55	1.99	1.39	2.55	1.77	-0.94	0.35	1.02
MCM3	6.13	5.74	5.49	6.17	5.93	5.49	4.68	5.33	5.28
MCM4	5.72	5.65	6.09	5.62	6.55	5.55	3.54	4.42	5.5
MCM5	5.23	4.76	5.38	5.87	5.81	5.32	4.62	4.54	5.17
MCM7	6.84	5.94	5.84	6.52	6.35	6.26	5.22	5.87	6.56
MCTS1	5.93	5.68	5.37	5.38	5.57	5.54	4.9	5.66	5.37
MEF2D	3.86	4.64	4.8	4.79	4.92	4.26	4.44	4.15	3.79
MELK	4.01	3.07	3.73	3.03	4.24	3.41	0.64	2.56	3.2
MERTK	1.59	1.86	2.08	1.98	2.04	2.87	3.1	1.92	3.75
MET	4.85	1.7	4.48	3.7	4.99	4.63	5.68	5.08	0.96
MFGE8	5.56	6.28	6.01	8.35	6.06	6.08	6.16	5.98	6.49
MFSD11	3.72	4.39	3.97	4.06	4.37	4.31	3.82	3.86	4.22
MKI67	4.93	3.49	4.7	3.52	5.1	3.74	1.13	3.27	2.56
MKKS	4.78	4.33	4.54	4.49	4.67	4.39	4.12	4.11	4.88
MME	1.46	2.41	1.14	1.66	1.27	1.56	4.93	1.48	-1.64
MMP1	4.71	1.83	5.09	1.06	5.81	4.88	0.59	-0.2	-1.09
MMP11	5.72	7.38	4.96	3.18	5.4	5.24	3.44	4.32	1.84
MMP14	6.35	7.14	6.65	8.06	6.8	6.76	6.33	7.86	6.23
MMP17	0.39	2.43	0.48	5.74	1.32	1.67	-1.06	3.16	2.59
MMP19	1.77	2.68	2.63	1.32	2.44	2.76	1.74	3.02	2.03
MMP2	6.37	7.46	6.68	5.94	6.6	6.91	4.77	7.07	5.74
MMP3	3.54	2.63	2.08	-1.73	3.68	0.1	-5.57	-0.27	-2.31
MMP9	4.23	4.88	3.92	4.59	3.91	5.05	2.06	3.61	3.87
MNAT1	3.98	4.82	3.88	4.19	4.44	4.72	3.99	4.13	4.4
MNDA	1.37	2.91	2.5	1.84	1.69	3.03	3.53	3.29	3.47
MPP1	4.11	3.62	3.38	4.45	2.99	3.72	5.04	4.26	4.71

MRPL12	7.03	6.03	6.05	6.65	6.11	6.44	5.62	6.5	6.23
MRPL37	6.49	6.11	6.04	6.42	6.16	5.97	5.82	6.09	6.07
MRPS16	6.51	6.56	5.92	6.08	5.74	6.26	5.73	6.35	5.58
MRPS28	5.81	5.92	5.12	5.67	5.36	5.87	5.58	5.48	6.6
MRTO4	4.92	4.61	4.34	5.03	4.78	4.68	4.04	4.44	5.15
MS4A1	-1.18	0.06	1.04	-0.73	-1.06	1.21	-0.71	-1.28	-3.82
MSN	5.35	6.62	6.44	8.08	7.15	7.49	8.02	7.88	7.62
MT1F	3.63	3.91	4.03	3.8	4.08	3.89	4.77	5.19	5.91
MT3	0.03	-2.55	-3.05	-1.64	-3.31	-0.62	3.57	1.39	10.89
MTA1	5.09	5.38	5.49	5.11	6.22	5.22	4.34	5.02	5.52
MTFR1	4.5	4.36	4.32	3.66	4.64	4.29	4.26	3.22	3.5
MTHFD1	5.59	5.33	5.28	5.66	5.6	4.99	5.31	4.79	5.48
MVD	5.33	4.1	4.45	4.5	4.68	4.86	3.95	4.61	4.68
MX1	4.3	5.54	5.34	4.85	5.64	5.32	4.81	5.65	5.04
MX2	2.76	3.47	4.16	3.83	4.33	4.17	2.48	3.64	2.51
MXI1	4.22	4.47	3.86	5.4	4.31	4.33	5.88	3.99	4.84
MYBL1	-0.18	1.54	0.28	1.37	0.71	0.49	0.81	0.35	1.48
MYBL2	5.39	4.09	5.26	4.38	5.82	4.96	0.82	3.65	4.31
MYCBP	4.82	5.02	4.98	3.93	4.99	4.55	3.49	4.33	3.68
MYCT1	0.28	1.83	1.21	0.06	0.78	1.05	3.67	0.33	1.8
MYH2	-9.97	-9.97	-9.97	-9.97	-9.97	-6.51	-9.97	-6.51	-9.97
MYL6	10.65	10.92	10.05	10.07	9.87	10.68	10.73	11.4	11.11
MYO1F	2.19	2.7	3.27	2.51	2.61	3.06	3.58	3.55	3.69
NCAPD2	5.03	4.48	5.06	4.88	5.88	4.88	3.45	4.24	4.44
NCAPG	3.24	2.66	3.15	3.13	3.82	2.78	0.38	2.13	3.04
NCAPG2	3.86	3.52	3.28	3.8	3.96	3.49	2.21	2.82	3.68
NCAPH	4.07	3.1	3.33	3.4	3.92	3.63	0.52	2.25	3.25
NCEH1	3.63	3.07	4.02	1.73	4.08	3.51	4.48	2.37	2.77
NCF2	2.44	3.1	2.44	2.38	2.63	4.16	3.29	3.06	3.39
NCF4	2.75	2.65	2.8	2.83	2.03	3.51	3.1	3.57	3.46
NCKAP1L	1.68	2.9	2.8	2.5	1.99	3.09	3.48	3.11	3.58

NCLN	5.99	5.02	5.71	5.78	5.92	5.26	4.7	5.35	5.02
NCOA3	3.11	3.63	3.75	2.97	4.44	3.63	3.15	3.04	2.49
NDC80	3.1	2.9	3.22	2.98	3.71	3.1	1.13	2.92	3.23
NEK2	4.22	3.98	3.64	3.1	4.02	3.62	0.26	2.47	2.84
NEO1	5	4.58	4.37	4.24	4.52	4.28	4.65	5.1	4.57
NID1	3.79	4.64	4.86	4.85	4.84	4.04	5.57	3.01	4.69
NLN	3.56	3.75	3.01	3.21	3.72	3.42	2.81	2.89	3.63
NME1	7.44	7.08	6.68	7.78	6.79	7.24	5.84	7.33	7.78
NMI	4.51	4.23	4.8	3.42	4.9	4.4	4.32	4.58	3.95
NMU	2.09	-0.11	3.01	-1.47	3.43	2.15	-1.51	5.8	0.84
NOP16	5.64	4.66	4.67	5.11	4.85	4.68	4.42	4.99	4.76
NOTCH2	3.07	4.89	3.86	4.38	4.53	3.87	3.91	4.35	4.31
NPC2	7.46	7.83	7.67	8.57	7.4	8.74	8.15	8.59	8.29
NPL	2.59	3.38	3.06	4.44	3.34	4.35	4.12	3.77	4.64
NRIP3	-1.06	1.25	-0.78	1.43	0.11	0.94	-1.09	-0.83	1.32
NTN3	-6.51	-4.61	-4.29	-3.82	-3.38	-4.29	-6.51	-2.93	-2.05
NUDT1	5.56	4.99	4.44	5.91	5.04	5.41	4.16	5.4	5.88
NUDT15	4.56	3.61	3.84	3.62	4.01	3.77	3.56	3.11	2.99
NUP107	4.66	4.81	4.49	4.47	4.84	4.6	4.06	4.58	4.3
NUP35	3.6	3.56	3.53	3.31	3.74	3.42	3.26	3.09	3.89
NUP85	5.21	5.24	4.66	5.03	5.14	5.2	4.4	5.36	5.21
NUP93	5.06	4.83	4.53	5.7	4.99	4.98	4.22	4.99	5.21
NUPL1	4.24	4	4.09	3.89	4.45	3.89	3.98	3.15	3.59
NUSAP1	4.88	4.68	4.98	4.72	5.37	4.62	3.54	3.9	5.11
NUTF2	7.08	6.56	6.89	6.83	6.75	6.73	6.3	6.97	7
OAS1	6.11	5.21	6.13	4.35	6.02	5.31	5.45	5.36	4.42
OAS2	3.14	4.58	4.88	4.04	5.36	4.3	3.98	3.86	3.23
OAS3	4.18	4.3	4.97	3.67	5.62	4.22	3.54	4.15	3.49
OASL	2.51	2.65	3.05	1.74	3.44	2.37	2.14	2.5	1.58
OCLN	4.33	4.26	4.77	-0.73	4.24	3.77	2.3	1.25	0.88
OIP5	2.96	2.06	2.69	1.88	3.03	2.38	0	1.52	2.59

OSBPL3	3.35	2.01	3.71	2.75	4.2	3.32	3.06	2.99	3.56
OSBPL8	3.06	4	3.83	3.75	4.28	3.62	4.01	2.88	3.66
OSCAR	0.71	1.74	1.28	1.11	0.77	2.21	1.59	2.28	2.36
P2RX5	0.93	-0.04	0.75	0.5	0.47	1.32	-0.86	1.08	0.02
PA2G4	7.28	6.74	6.63	6.99	6.78	6.68	6.35	6.49	6.55
PAICS	6.46	6.14	5.56	6.29	5.99	5.82	5.18	5.48	6.21
PAK1	5.64	5.21	5.49	4.36	5.63	5.51	4.72	4.58	4.65
PAK2	4.72	5.14	4.81	4.66	5.62	4.98	4.7	4.51	4.76
PARP1	5.69	6.59	5.86	6.46	6.23	6.03	5.01	5.69	6.68
PARP12	4.6	4.61	4.92	3.99	5.15	4.75	4.46	4.75	3.84
PARP14	3.67	4.22	4.33	3.67	4.91	4.35	4.53	4.05	3.3
PARP4	5.44	4.16	5.12	4.38	5.02	4.2	4.56	4.23	3.81
PARP9	4.45	5.43	4.83	4.54	5.23	5.18	4.71	5.2	5.24
PBK	3.71	3	3.54	3.81	3.83	2.86	0.48	2.56	4.27
PBXIP1	4.92	5.97	5.06	5.87	4.92	5.81	6.19	6.22	6.33
PCSK7	4.98	4	4.85	3.98	4.77	4.17	3.93	4.48	4.45
PDAP1	7.24	6.93	6.15	7.26	6.1	7.08	6.62	7.12	7.33
PDCD1	-0.49	-0.65	0.56	0.71	-0.16	0.52	0.35	0.84	-1.83
PDGFD	0.41	2.61	1.55	1.46	0.78	0.9	4.82	3.04	1.83
PDIA4	7.41	6.74	6.75	6.72	6.73	7.44	6.36	7.17	6.81
PDK2	4.85	4.94	5.34	4.83	4.83	4.38	5.9	4.4	5.36
PDLIM7	5.23	5.48	5.86	5.44	6.06	5.46	4.9	5.99	5.57
PDSS1	4.13	2.37	3.47	2.31	3.24	2.54	1.98	1.75	2.84
PECAM1	3.82	5.02	5.47	3.89	4.54	4.76	7.03	3.86	4.62
PFKP	5.82	4.43	5.75	5.38	6.22	6.01	8	5.18	5.72
PFN1	9.52	9.13	9.63	9.58	9.47	9.19	8.93	9.53	9.09
PGM2	4.21	4.24	4.01	4.26	4.63	4.18	3.82	4.19	4.16
PHF19	5.24	4.03	4.61	5.46	4.76	4.5	3.23	4.27	5.62
PHLDA1	4.71	3.26	3.94	6.26	4.85	4.14	4.87	2.48	5.58
PHLDA2	5.55	3.13	6.11	4.76	6.21	4.57	2.09	3.72	1.52
PIK3CD	0.74	1.81	2.13	4.27	2.61	2.63	2.8	3.05	2.01

PIK3CG	-0.85	0.18	0.85	-0.66	-0.09	0.33	1.01	-0.45	0.14
PIM2	4.14	3.8	4.3	3.87	3.76	5.06	3.61	3.44	3.07
PIR	3.31	3.3	3.04	6.51	3.8	4.49	3.15	2.99	3.91
PITPNC1	3.86	3.68	3.4	3.52	3.51	3.91	4.94	3.56	5.11
PKIG	5.06	5.28	4.78	5.86	4.4	4.96	6.23	5.57	6.49
PLA2G7	2.07	1.93	2.79	1.8	2.29	2.86	1.94	0.38	0.9
PLA2R1	1.38	2.22	1.17	-0.58	2.34	1.58	1.37	2.18	-0.41
PLAC8	5.57	1.33	5.88	0.81	4.85	3.87	2.27	2.51	2.01
PLAT	4.32	5.24	4.74	6.43	5.05	4.77	4.31	3.96	5.43
PLAUR	5.86	4.97	5.99	4.81	5.87	5.79	3.94	6.54	4.77
PLCG2	1.22	2.45	2.63	2.26	2.77	2.9	3.4	2.43	2.26
PLD3	6.79	7.47	7.16	8.07	7.07	8.17	7.47	7.52	7.91
PLEK	2.23	3.22	3.02	2.94	2.18	3.62	3.7	3.48	3.58
PLG	-0.91	-3.46	-1.64	-4.29	-1.94	-1.73	-0.25	-2.93	-3.63
PLK1	5.11	3.84	5.09	4.47	5.43	4.37	1.11	3.49	4.14
PLK4	2.87	2.56	2.52	2.25	3.18	2.36	0.68	1.58	2.39
PLOD2	4.46	4.53	4.16	3.67	4.84	5.03	6.46	7.09	5.17
PLSCR1	6.31	5.4	6.05	5.45	6.36	5.99	6.15	5.48	6.11
PLTP	5.64	5.35	5.92	7.59	6.42	6.92	5.41	7.41	8.29
PLVAP	5.29	5.76	6.15	5.3	5.82	5.09	8.61	4.98	4.47
PLXNB1	5.42	5.14	4.87	4.22	5.77	4.89	4.3	5.26	5.93
PMAIP1	2.96	3.23	2.46	2.45	3.72	3.78	0.66	2.46	1.06
PML	5.04	4.96	5.7	5.53	6.24	5.33	5.54	5.72	4.58
PNN	5.12	5.1	5.17	4.72	5.55	5.01	4.53	4.84	4.72
PNO1	4.2	4.12	3.74	4.07	4.25	4.22	3.49	3.73	4.24
POLE2	2.86	2.59	2.92	2.01	3.43	2.59	0.6	1.28	2.52
POLR3K	4.44	4.67	3.38	4.38	3.32	4.1	2.99	3.82	4.04
POR	6.19	5.62	5.74	5.86	6.1	6.39	5.42	5.45	6.21
POU2AF1	1.03	0.72	2.69	0.43	2.18	3.17	-0.64	-0.45	-2.31
PPIH	5.99	5.58	5.33	5.28	5.5	5.47	4.63	5.54	5.79
PPP3CA	3.71	4.21	4.1	3.26	4.24	3.98	3.55	3.7	4.29

PRAME	-1.47	-1.69	-0.47	8.11	2.04	3.5	1.21	-4.29	-2.73
PRC1	5.43	5.2	5.11	5.45	5.81	5.18	3.09	4.81	5.33
PRF1	1.25	0.82	1.77	2.09	1.14	2.31	3.35	2.07	1.01
PRKCB	-0.43	0.06	1.44	0.53	0.25	1.06	1.02	0.66	1.7
PRRG3	-4.04	-2.83	-2.31	-4.29	-2.88	-2.18	-1.88	-2.47	-1.78
PRTN3	-9.97	-4.04	-5.57	-9.97	-9.97	-5.01	-4.04	-2.63	-1.86
PSMA7	8.46	7.76	7.56	8.02	7.81	7.99	7.12	7.99	8.24
PSMB10	6.97	5.96	5.97	6.69	5.76	6.26	6.36	6.61	6.34
PSMB8	7.43	6.53	6.85	7.05	6.67	6.77	7.31	7.48	6.74
PSMB9	6.79	5.62	6.62	6.37	6.41	6.37	6.85	7.39	6.38
PSMC3	6.69	6.86	6.33	6.96	6.45	6.83	6.67	7.11	7.35
PSMD12	4.73	5.09	4.87	4.75	5.53	5.1	4.42	4.5	4.53
PSMD14	5.74	5.59	5.09	5.41	5.68	5.8	4.82	5.52	6.06
PSMD2	7.05	7.35	6.75	7.5	7.41	7.7	6.64	7.42	7.75
PSME1	8.1	8.21	7.6	7.68	7.56	7.93	8.06	7.96	7.32
PSME2	8.23	7.91	7.55	7.82	7.35	7.93	7.37	7.85	7.22
PTGDS	3.72	4.19	5.34	5.76	3.66	5.92	3.55	6.7	7.95
PTPN7	1.69	1.78	2.34	2.12	1.78	2.56	2.2	2.34	1.74
PTPRB	2.55	1.84	2.33	0.64	1.92	1.55	4.18	0.42	1.32
PTPRC	2.45	3.48	3.97	2.61	3.04	3.86	4.32	3.29	3.15
PTPRU	1.25	3.38	3.1	1.98	3.12	3.87	3.4	4.15	1.92
PTS	4.8	4.81	4.3	4.03	4.59	4.87	4.44	4.93	5.11
PTTG1	6.31	5.32	5.77	6.54	6.14	5.93	3.34	4.98	6.11
PXN	5.97	5.42	5.89	6.37	6.11	5.49	5.98	5.46	5.25
PYHIN1	-1.07	-0.06	0.63	0.71	-0.39	0.23	1.14	-0.04	-2.83
RAB3B	-2.02	-2.11	-0.78	-2.83	-0.11	-1.69	-5.57	-2.24	-1.78
RAB8A	5.47	5.68	5.78	5.14	5.85	5.14	5.23	5.5	5.39
RAC1	8.13	8.03	8.47	7.96	8.79	8.14	7.78	7.85	8.34
RAC2	4.87	4.88	5.44	4.82	5.33	5.77	4.93	5.08	3.9
RACGAP1	4.92	4.42	4.56	4.32	5.05	4.47	2.95	3.78	4.6
RAD21	6.35	6.98	6.24	6.14	6.78	6.3	5.52	6.22	6.3

RAD51AP1	3.59	2.96	3.26	2.59	3.83	3.24	1.31	2.2	2.94
RAD54B	2.1	2.2	2.27	1.96	2.78	1.96	1.31	0.89	2.39
RAD54L	3.16	1.97	2.17	2.64	2.79	2.56	-0.01	1.8	2.28
RAF1	5.15	5.64	5.18	5.46	5.63	5.31	4.97	5.35	5.95
RARRES3	5.04	7.58	6.34	5.2	5.41	6.12	7.24	7.41	6.78
RASGRP1	-0.62	1.51	1.03	0.02	1.47	1.4	2.52	1.51	1.54
RASSF2	1.18	2.62	2.25	3.12	1.69	2.34	3.41	2.38	5.87
RASSF4	2.66	3.09	3.44	3.36	2.64	3.97	6.1	3.83	5.99
RBBP4	6.36	7.18	6.4	6.66	6.57	6.51	6.28	6.41	7.24
RBM14	4.95	4.89	5.1	4.91	5.31	4.7	4.29	4.69	4.9
RBMX	7	7.02	6.71	6.58	6.91	6.71	6.84	6.77	7.08
REV3L	1.91	2.5	2.59	2.91	3	2.2	2.36	2.4	3.54
RFC3	4.16	3.36	3.74	3.55	3.98	3	2.08	2.2	3.34
RFC4	4.72	4.5	4.3	4.51	4.76	4.92	3.45	4.41	4.88
RFX5	4.19	4.88	4.63	4.81	4.56	4.57	4.09	4.11	4.44
RFX7	1.31	2.06	2.36	1.82	2.72	1.54	1.64	1.57	1.83
RGS4	-0.02	2.4	1.14	0.23	0.69	1	1.31	2.59	2.22
RGS5	4.25	5.1	6.28	3.39	5.04	4.19	9.08	3.7	5.33
RGS8	-6.51	-4.29	-6.51	-9.97	-6.51	-6.51	-7.85	-5.01	-0.28
RHOA	8.4	8.65	8.19	8.5	8.05	8.21	8.11	8.47	8.84
RHOB	6.36	7.91	6.85	5.93	6.95	6.56	8.47	7.22	8.04
RHOC	8.15	8.59	7.66	8.12	7.25	7.94	8.38	8.44	9.1
RHOG	5.68	5.71	6.21	6.02	6.22	5.79	5.74	6.17	6.23
RHOJ	1.12	2.15	2.34	3.96	1.88	1.46	3.51	1.02	4.13
RHOQ	3.85	4.92	4.52	6.26	4.52	4.98	5.29	5.42	5.63
RMND5B	4.4	5.16	4.23	4.23	4.44	4.33	4.48	4.23	4.75
RNASE6	3.22	3.98	3.86	3.66	2.96	3.94	4.67	4.6	4.97
RNASEH2A	5.47	5.17	4.5	5.42	4.86	4.96	3.81	4.86	5.79
RND3	4.29	4.11	3.86	4.22	4.95	4.52	4.23	4.75	4.44
RNF138	3.74	3.82	3.98	3.55	4.17	3.79	3.44	3.37	4.26
RNF41	4.45	4.72	4.47	4.41	4.36	4.57	4.56	4.47	5.17

ROBO4	1.75	2.48	2.79	1.73	2.65	2	4.76	2.54	3.71
RPL23AP7	2.47	2.77	2.26	3.15	2.1	2.89	2.92	2.92	4.15
RPN1	7.59	7.66	7.5	7.48	7.64	7.77	7.25	7.76	7.46
RPP40	3.54	3.32	2.95	3.09	3.39	3.21	1.91	2.76	3.61
RPS28	10.09	9.41	9.91	9.9	9.71	9.39	9.47	10.04	9.68
RPS6KB1	3.25	3.9	3.45	3.18	3.95	3.6	3.35	3.49	3.31
RRM2	5.91	4.49	5.79	4.83	6.14	5.06	2.4	3.94	4.85
RRM2B	2.93	3.71	3.27	3.34	3.53	3.48	4.29	3.29	3.86
RSAD2	1.31	3.16	2.34	0.91	2.78	2.37	2.05	3	2.58
RTP4	2.18	2.45	3.01	3.67	2.84	2.46	3.55	3.05	2.38
RUNX3	1.84	2.03	2.81	4.72	3.32	2.88	2.75	1.44	1.1
RUVBL1	5.68	5.57	4.72	5.54	5.32	5.65	4.52	5.49	5.73
S1PR1	1.97	3.02	3.5	1.84	2.72	2.63	5.31	2.32	4.62
SAMD9	1.61	2.57	3.13	1.98	4.45	2.35	2.15	3.27	2.12
SAMHD1	3.99	4.73	4.96	4.08	4.71	4.62	4.89	4.76	4.84
SAMSN1	1.59	2.43	2.9	1.53	2.38	2.87	2.66	2.86	3.65
SAR1A	5.17	5.94	5	5.18	5.12	5.47	5.43	6.08	5.17
SAR1B	4.82	5.42	4.87	4.51	4.92	4.95	5.13	4.44	5.69
SASH3	1.99	2.86	2.77	2.64	1.84	3.24	3.49	3.27	3.99
SAT1	8.48	8.11	8.31	8.47	8.62	9.18	8.48	8.79	8.83
SATB1	3.01	2.27	3.02	2.56	2.91	2.53	2.52	1.72	3.33
SCD	6.66	6.76	5.43	6.95	6.39	6.52	6.46	4.24	6.42
SDC1	6.85	6.99	6.59	3.72	7.85	8.09	5.58	4.16	2.75
SDCBP	6.72	6.7	6.77	9.08	6.83	6.73	6.81	6.88	7.49
SECTM1	3.44	4.31	4.86	2.86	4.17	4.16	3.54	5.39	3.12
SELENBP1	6.77	6.82	5.18	5.08	3.92	5.24	4.53	6.51	3.78
SELL	0.95	2.51	2.66	2.23	1.47	2.72	2.48	2.3	2.36
SELPLG	2.6	3.85	3.51	3.34	2.71	3.85	4.02	4.05	4.59
SEMA3F	3.64	5.38	4.02	2.35	5.31	4.33	5.5	4.1	2.64
SERPINE1	3.38	4.3	4.5	3.63	5.53	5.18	6.04	6.08	5.71
SERPING1	5.98	7.84	7.49	7.29	6.8	7.62	8.82	9.56	7.47

SF3A1	5.6	5.74	6.53	6.41	6.56	5.61	5.47	5.12	5.66
SFTPB	-4.61	-5.01	-3.63	-6.51	-4.04	9.63	-5.01	-5.01	-5.57
SH2B3	2.48	3.11	3.57	3.85	3.31	2.69	4.37	3.22	3.49
SH2D1A	-0.81	0.02	0.23	-0.28	-0.75	0.51	0.92	-0.1	-2.18
SH3BP5L	3.52	4.21	3.2	4.21	3.58	3.8	3.67	3.73	4.15
SHC1	5.77	6.46	5.96	7.04	6.37	6.41	6.37	6.87	5.45
SHCBP1	2.56	2.04	2.32	2.95	3.01	2.38	0.44	2.32	2.54
SHFM1	8.14	7.81	7.81	8.09	8.4	8.15	7.48	8.48	7.98
SIGLEC10	1.03	1.64	2.25	1.08	1.35	2.03	2.65	1.91	3.94
SIGLEC14	-0.55	0.61	0.61	0.25	-0.46	1.02	1.24	1.02	2.31
SIGLEC5	-1.78	-0.91	-0.76	-1.69	-1.86	-0.78	-0.91	-0.78	0.25
SIGLEC7	-0.31	0.72	0.96	0.69	-0.03	1.14	1.56	1.63	2.33
SIGLEC9	-0.26	0.92	0.52	0.86	-0.17	1.23	1.65	1.83	2.88
SKA1	2.04	1.51	2.29	1.96	2.45	1.82	-0.91	1.21	1.84
SKA2	4.88	5	5.18	5.06	5.45	4.98	4.43	5.13	6.05
SKP2	3.64	2.76	3.69	2.74	4.13	3.43	1.82	3.89	3.2
SLA	1.76	2.74	2.74	2.23	2.09	3.13	3.65	3.04	3.95
SLAMF1	-0.76	-0.54	0.14	-0.64	-0.77	0.19	-0.36	-0.97	-2.47
SLAMF6	0.44	0.59	1.33	0.54	0.47	1.38	1.67	0.3	-1.25
SLAMF7	1.97	2.41	3.08	3.33	2.42	3.79	2.63	2.52	-0.62
SLAMF8	2.25	2.9	3.03	3.12	2.46	3.71	3.22	3.19	2.49
SLC16A1	5.22	2.96	4.87	4.77	5.27	3.73	4.8	6.13	5.71
SLC1A3	0.03	2.38	1.82	2.17	2.63	2.62	2.79	3.3	8.52
SLC25A40	3.5	3.66	3.57	3.65	4.02	3.41	3.53	2.61	3.68
SLC25A5	9.24	8.09	8.34	8.67	8.54	8.54	7.51	7.78	8.3
SLC35E2	0.92	1.59	2.01	1.18	2.19	1.09	1.11	0.88	1.6
SLC40A1	5.98	5.98	6.12	3	5.05	5.39	6.15	4.39	4.8
SLC5A3	1.51	2.03	1.53	2.42	2.21	2.06	2.91	1.37	1.69
SLC7A5	5.58	4.39	4.97	7.35	6.64	5.34	3.4	3.61	5.96
SLC7A7	3.59	3.49	4.28	3.11	3.45	3.89	4.87	5.55	4.3
SLCO2B1	4.23	3.69	4.73	3.2	3.34	4.03	4.93	4.25	5.71

SLFN13	0.13	1.74	2.77	0.56	3.17	2.98	3.76	2.62	0.57
SLPI	6.62	5.31	7.38	-0.55	8.2	8.81	2.2	10.28	4.35
SMC2	3.26	3.1	3.25	2.66	4.02	2.72	2.3	2.62	3.2
SMO	1.56	2.95	1.71	3.91	2.53	2.81	3.75	3.93	4.56
SMS	6.43	6.57	5.87	6.39	6.01	6.14	6.13	5.83	6.39
SMURF2	3.08	3.46	3.29	2.89	3.75	3.71	3.1	3.21	3.29
SNRPA	6.26	5.83	6.85	5.97	6.88	5.83	5.29	5.93	5.82
SNRPA1	5.83	5.52	5.33	5.36	5.63	5.81	4.85	5.44	5.48
SNRPB	8.23	7.56	7.94	7.88	8.12	7.86	6.5	7.58	7.4
SNRPC	7.26	7.02	6.7	7.57	6.73	7.13	6.29	7	7.34
SNRPD1	6.71	6.49	6.64	6.31	6.89	6.54	5.52	6.59	6.25
SNRPE	7.42	7.84	6.97	7.41	7.11	7.42	6.77	7.33	7.26
SNX10	2.52	3.62	3.37	5.17	3.82	3.65	5.7	3.3	4.9
SNX17	6.01	6.34	5.63	6.64	5.87	6.35	6.3	6.66	6.88
SOS1	2.95	3.5	4.12	3.11	4.41	3.24	3.59	2.89	3.2
SOX11	-3.63	-1.6	-3.31	-5.01	-3.05	-4.61	-1.39	-0.91	2.86
SP100	5	5.54	5.59	5.37	5.78	5.48	5.69	5.94	5.06
SP110	3.48	4.65	4.35	4.28	4.35	4.13	4.19	4.49	4.03
SP140	0.29	1.13	1.67	0.92	1.3	1.8	1.12	1.45	0.79
SPAG17	-4.04	-0.3	-2.47	-3.17	-0.11	0.76	1.35	0.57	-1.18
SPARC	9.41	11.02	9.6	10.67	8.95	9.76	11.07	11.24	11.77
SPARCL1	4.43	6.63	5.77	4.46	5.49	5.81	7.72	5.44	9.68
SPC25	3.54	2.81	3.03	2.7	3.18	2.74	0.59	1.96	3.43
SPOCK2	2.01	2.36	3.16	2.38	2.45	3.37	4.78	7.43	5.62
SPP1	5.68	7.1	5.58	7.85	5.99	8.27	10.13	8.45	11.38
SPRYD7	3.15	2.32	2.59	2.53	2.86	2.4	2.87	2.31	2.78
SQLE	5.47	5.38	4.3	4.56	5.49	5.24	3.04	4.52	4.54
SQRDL	6.37	6.05	5.44	4.19	5.8	5.77	6.01	5.45	4.47
SRGN	5.19	5.82	6.45	5.87	5.73	6.71	7.16	6.72	7.23
SRM	6.35	6.02	6.84	7.63	7.18	6.17	5.3	6.73	6.81
SRP9	7.61	8.38	7.43	7.13	7.64	7.72	7.23	7.27	8.29

SSR3	6.71	7.1	7.24	6.57	7.32	7.1	6.58	6.82	6.83
SSSCA1	5.62	5.72	5.18	5.75	5.21	5.8	5.42	5.81	5.59
STAT1	5.82	6.47	6.5	6.6	6.83	6.51	6.14	6.04	5.76
STAT2	4.83	5.82	5.36	6.34	5.74	5.97	5.57	6.21	5.65
STAT4	-0.27	0.51	0.93	-0.12	0.86	1.33	1.42	0.46	-0.83
STAT5A	3.83	4.22	4.01	5.05	3.78	3.45	3.72	4.67	3.37
STAT5B	4.05	4.33	4.13	4.32	4.31	3.9	4.81	3.94	4.46
STAT6	6.46	6	6.57	6.26	6.8	6.33	6.72	6.21	3.95
STIL	2.48	1.9	2.86	1.6	3.2	1.84	-0.07	0.8	1.61
STK17A	3.15	3.55	3.41	3.55	4.13	4.41	3.55	3.71	4.91
STRA13	6.45	6.19	6.05	5.9	6.01	6.19	5.09	5.93	6.35
SVIL	3.72	4.11	4.24	3.92	5.15	4.32	3.87	3.17	1.32
SYK	4.51	3.12	3.92	2.04	4.07	3.94	3.18	2.67	2.99
TAF2	3.5	4.01	3.98	3.47	4.61	3.65	3.2	3.08	3.41
TAGLN	7.88	8.81	8.2	6.16	7.06	7.86	8.27	8.94	7.51
TAP1	6.45	5.61	6.2	6.47	6.42	6.09	6.12	6.28	5.79
TAP2	5.12	4.24	4.69	5.68	5.12	4.95	4.74	5.13	4.45
TAPBP	6.26	6.47	7.17	6.73	7.3	6.63	6.94	7.04	5.78
TARS	6.24	6.03	5.89	5.91	6.28	6.25	5.49	6.18	5.62
TAS2R5	-1.28	-1.35	-1.64	-2.31	-1.03	-1.35	-0.86	-1.39	-0.73
TBRG1	3.95	3.71	3.9	3.76	3.98	4.1	4	4.07	4.19
TBX21	-2.51	-1.47	-1.32	-1	-2.11	-0.91	0	-0.94	-2.55
TBXAS1	4.18	3.32	3.41	3.29	2.65	3.71	4.39	3.65	4.49
TCEB1	6.38	6.56	6.1	6.47	6.22	6.61	5.98	6.37	6.88
TCF7L2	4.28	3.5	4.88	2.97	4.61	3.32	3.48	3.5	3.16
TEAD4	4.32	3.64	4.26	4.07	4.37	3.54	3.79	4.79	2.9
TEK	0.27	1.57	1.11	-0.34	0.79	0.63	3.17	0.83	0.9
TFEC	-0.59	1.26	0.87	0.1	0.03	1.13	3.59	1.18	1.5
TFPI2	-0.4	0.94	0.25	1.06	0.32	2.35	3.75	4.33	-0.23
TFRC	6.47	5.11	5.63	4.75	6.52	5.79	4.19	5.04	5.17
TGFB1	3.97	4.78	5.33	4.92	5.93	5.3	5.98	5.75	5.43

TGFB1I1	4	4.44	4.73	4.77	4.41	3.92	4.58	4.83	4.97
TGFB3	1.99	5.27	2.61	2.75	2.57	3.56	3.46	5.85	4.45
TGFBI	8.76	6.5	6.31	6.24	6.63	6.98	8.11	9.01	6.98
TGFBR1	3.25	3.96	3.93	4.18	4.39	3.55	4.24	3.59	4.23
TGIF1	6.54	6.39	6.18	5.97	6.31	6.43	5.62	6.16	5.81
TGIF2	3.82	3.9	3.59	3.8	3.66	3.6	3.1	3.41	3.06
THBS1	5.24	6.89	5.98	3.66	6.06	5.26	6.27	4.53	3
THBS2	4.34	5.91	4.5	3.77	4.36	4.84	3.77	6.91	3.98
THPO	-3.46	-0.15	-2.55	-3.31	-2.83	-1.69	-0.43	-0.55	-0.53
TIE1	1.94	3.1	2.87	2.19	2.52	2.61	4.71	2.22	3.42
TIMP1	8.68	8.92	9.73	9.55	8.82	9.16	9.08	10.88	9.16
TIMP2	5.98	7.22	6.55	8.56	6.29	6.45	6.55	8.31	7.98
TK1	5.9	5.47	5.63	5.38	6.07	6.02	3.72	5.02	4.08
TLR2	0.66	2.24	1.51	1.63	2.33	2.97	2.84	3.15	3.08
TM4SF18	0.66	2.72	1.9	0.82	1.2	0.9	5.62	0.59	3.31
TMEM130	-2.55	-0.38	-0.81	-3.46	-1.83	0.55	1.97	2.3	1.82
TMEM176B	7.96	6.18	7.33	5.39	6.14	6.54	9.34	8.2	7.01
TMPO	5.59	5.16	5.6	4.79	5.7	5.16	4.54	4.56	5.14
TMSB15A	-0.5	0.89	-0.95	1.04	-0.94	-0.28	0.03	0.23	3.72
TNC	4.07	5.42	4.64	6.5	5.71	5.66	4.23	4.02	7.07
TNF	-0.77	0.23	-0.56	-1.21	-0.27	-0.22	-1.15	-0.47	-0.27
TNFAIP2	4.47	5.87	5.54	4.69	6.07	6.29	5.86	7.1	4.55
TNFAIP3	3.78	3.3	4.68	3.26	4.89	4.09	4.39	3.31	2.56
TNFRSF12A	6.15	5.85	6.04	5.64	6.1	6.22	6.51	7.22	6.62
TNFRSF17	-0.2	-0.03	0.26	-1.12	-0.82	1.55	-1.64	-1.99	-3.82
TNFRSF1A	6.13	6.6	6.7	6.19	7.02	6.89	6.86	7.49	7.06
TNFRSF1B	4.57	3.48	4.41	3.61	3.6	3.93	4.66	4.51	4.02
TNFSF10	5.31	6.68	5.46	3.01	6.11	6.49	7.28	4.34	3.31
TNFSF12	3.56	4.65	4.2	5.13	3.62	4.46	5.84	4.91	5.57
TNFSF13B	1.09	2.56	2.74	2.32	2.21	2.66	2.71	3.74	3.42
TNXB	0.83	2.06	2.72	1.04	1.91	1.84	2.35	6.73	3.46

TOMM40	6.29	5.51	5.84	5.85	6.11	5.77	4.82	5.43	5.59
TOP2A	5.44	4.98	5.39	4.59	6.21	5.09	2.25	4.41	5.14
TP53INP1	1.97	4.38	2.6	2.64	2.99	3.51	3.66	3.26	3
TPI1	9.5	8.89	8.72	9.37	9.12	9.69	10.24	9.24	9.89
TPM1	7.89	7.97	7.42	5.47	6.97	6.77	8.59	8	6.17
TPM2	6.68	6.61	6.71	5.54	6.29	6.43	6.66	8.41	5.98
TPM3	8.4	8.57	8.23	8.49	8.29	8.24	7.78	8.1	7.74
TPO	-2.63	-0.86	-2.24	-4.29	-2.28	-3.46	-5.57	-3.46	-6.51
TPRKB	5.33	5.49	5.07	4.78	5.33	5.13	5.13	5.29	5.39
TPX2	5.46	4.49	5.09	4.89	5.71	4.72	1.85	3.96	4.51
TRA2B	6.37	6.5	6.43	6.41	6.78	6.46	5.85	6.29	6.61
TRAC	4.1	4.87	5.19	4.85	4.23	4.98	5.17	4.25	2.33
TRAF3	2.97	2.78	3.21	2.91	3.69	3.13	2.49	2.74	2.61
TRBV19	-1.2	0.09	0.2	-0.38	-1.51	0.33	0.69	-0.23	-3.17
TRBV21-1	-9.97	-9.97	-3.31	-9.97	-9.97	-3.46	-3.26	-9.97	-9.97
TREM1	2.03	1.65	1.78	1.16	1.81	4.65	1.2	3.01	3.74
TRIM21	4.37	4.02	3.99	3.91	3.83	3.98	4.48	4.26	3.98
TRIM22	3.64	4.71	4.34	3.93	4.46	4.92	5.67	4.93	5.27
TRIP13	3.67	3.11	3.42	3.25	4.08	3.9	1.17	2.64	2.74
TTF2	3.94	3.48	3.62	3.4	4.34	3.34	2.16	2.16	2.56
TTK	3.1	2.29	3.01	1.94	3.72	2.67	-0.28	1.44	2.35
TUBA4A	6.82	4.96	6.32	3.25	6.83	6.38	5.66	3.66	4.6
TUBG1	5.37	5.39	5.32	5.78	5.66	5.5	4.4	4.98	5.6
TYK2	5.55	5.62	5.5	5.86	5.91	5.72	5.96	6.04	6.12
TYROBP	5.65	6.99	7.06	6.93	6.14	7.32	7.76	7.91	8.24
UAP1	5.36	6.25	4.69	4.91	4.91	5.26	4.62	5.89	4.61
UBA7	4.45	4.49	4.56	4.38	4.49	4.39	4.6	4.89	4.78
UBD	6.24	4.55	6.62	4.91	5.03	5.49	6.77	3.09	2.62
UBE2C	7.03	5.74	6.55	6.12	6.69	6.26	2.59	5.11	5.95
UBE2J1	4.68	4.69	4.86	4.37	4.74	4.57	4.76	4.35	4.55
UBE2L6	5.86	6.75	6.1	6.52	6.14	6.51	6.76	6.84	7.17

UMPS	4.92	4.87	4.19	4.54	4.68	4.77	4.15	4.45	4.55
UQCR10	6.93	7	6.82	6.9	6.62	7.15	6.65	6.72	7.34
USP18	2.57	3.71	2.92	3.27	3.04	3.53	2.68	2.66	2.97
USPL1	3.08	2.98	3.01	2.73	3.28	2.49	2.89	2.3	3.18
VAMP4	2.37	3.3	2.6	2.96	3.07	3.15	2.82	2.33	3.71
VAT1	5.15	6.71	5.81	8.61	5.99	6.16	7.24	6.92	6.72
VAV1	1.6	2.27	2.13	1.74	1.61	3.13	2.56	2.72	2.68
VCAM1	2.21	3.67	3.34	2.7	2.88	3.46	7.77	4.8	4.66
VDAC1	8.19	7.46	7.54	7.7	7.51	7.09	7.87	7.12	7.44
VEGFC	0.61	2.52	2.1	1.06	2.06	1.7	3.11	2.87	-0.13
VSIG4	2.02	3.34	3.11	3.65	2.31	4.33	4.33	5.18	6.78
VTA1	4.95	5.13	5.12	4.52	5.46	4.87	4.43	4.63	5.14
VWF	4	4.88	4.47	4.11	4.69	4.69	7.84	4.69	5.66
WBP2	6.05	6.57	6.29	6.98	6.3	6.46	6.77	6.08	7.52
WDHD1	2.34	2.34	1.97	2.56	2.97	2.59	0.65	1.51	1.95
WDR54	3.41	4.1	3.12	4.47	3.63	4.4	5.21	4.41	5.27
WDR77	5.38	4.43	4.78	4.72	5.01	4.66	3.74	3.74	4.95
WIPF1	3.27	4.33	4.61	5.56	4	4.28	5.09	4.4	4.5
WNT2B	-0.01	-0.94	0.64	-1.6	1.16	-0.06	0.03	3.76	-0.15
WNT8B	-4.16	-4.61	-3.82	-6.51	-3.82	-4.61	-5.57	-6.51	-6.51
WSB1	4.91	4.94	5.09	5.03	5.46	5.82	5.47	5.76	5.76
WSB2	5.41	5.48	5.64	5.65	5.73	5.58	5.57	5.26	6.38
WTAP	5.74	5.91	5.53	5.13	5.93	5.91	5.82	5.66	6.66
XAF1	3.66	4.08	4.61	3.1	5.03	3.93	4.26	4.23	3.78
XCL2	-1.51	-0.03	-0.01	0.2	-1.28	0.46	1.23	1.34	-1.78
XRCC6	7.66	7.73	7.07	7.88	7.45	7.84	7.27	7.1	7.54
ZAP70	0.85	0.9	1.47	1.47	0.79	1.81	1.92	1.69	0.15
ZBP1	1.56	0.71	1.74	0.1	1.24	1.53	0.23	0.51	-0.78
ZC3HAV1	3.5	4.15	3.96	3.94	4.36	3.78	3.47	3.66	3.61
ZFP36L2	6.7	5.91	7.42	5.14	7.1	5.49	5.66	5.6	6.2
ZNF219	3.85	3.32	4.59	3.91	4.71	3.65	3.5	4.23	4.29

ZNF3	4.53	4.53	3.94	4.04	4.19	4.1	3.82	3.95	4.75
ZNF362	2.8	4.08	3.94	4.88	4.02	3.87	3.89	4.17	4.48
ZNF423	0.21	1.24	0.95	-0.76	0.53	-0.41	0.95	1.33	2.46
ZNF83	3.31	4.6	3.65	3.25	3.57	3.62	5.01	4.21	4.61
ZWILCH	3.31	3.24	3.4	3.17	3.92	3.06	1.6	2.78	2.93
ZWINT	5.51	5.09	4.8	4.75	5	4.95	3.13	4.2	4.06
ZYX	7	6.77	7.26	6.88	7.33	6.77	6.58	7.86	7.68

We normalized all gene expression as log2(transcripts per million+0.001). Small-cell lung cancer was excluded due to lack of molecular data from TCGA. We also excluded bladder cancer, ovarian cancer, and head and neck cancer from this analysis due to a substantial heterogeneity between trials. Abbreviations: COAD, colon cancer; BRCA, breast cancer; STAD, stomach cancer; SKCM, melanoma; ESCA, esophageal cancer; OV, ovarian cancer; NSCLC, non-small-cell lung cancer; KIRC, kidney clear cell carcinoma; MESO, mesothelioma; GBM, glioblastoma.

Supplementary table 7. The median levels of 31 genes significantly correlated with PD-L1 predictiveness for overall survival and progression-free survival across cancer types.

Variable	Overall survival		Progression-free survival	
	Rs	***P* value**	***Rs***	***P* value**
CXCL11	0.88	<0.01	0.9	0.04
ADAMDEC1	0.78	0.01	1	<0.01
BCL2L14	0.8	0.01	0.9	0.04
MSN	-0.67	0.05	-0.9	0.04
CTNS	-0.68	0.04	-0.9	0.04
DCBLD2	-0.68	0.04	-0.9	0.04
RHOQ	-0.68	0.04	-0.9	0.04
CORO1C	-0.72	0.03	-0.9	0.04
CNTNAP1	-0.73	0.02	-0.9	0.04
IFIT3	-0.73	0.02	-1	<0.01
MBP	-0.75	0.02	-0.9	0.04
CDKN1C	-0.77	0.02	-0.9	0.04
IFI44	-0.77	0.02	-0.9	0.04
LRP1	-0.77	0.02	-0.9	0.04
MAF	-0.77	0.02	-0.9	0.04
MATN1	-0.77	0.02	-0.9	0.04
SMO	-0.77	0.02	-0.9	0.04
UBE2L6	-0.78	0.01	-0.9	0.04
FADS1	-0.82	0.01	-0.9	0.04
MT1F	-0.82	0.01	-0.9	0.04
TRIM22	-0.83	0.01	-0.9	0.04
FAS	-0.85	<0.01	-0.9	0.04
CDKN1A	-0.87	<0.01	-0.9	0.04
GABARAPL1	-0.87	<0.01	-0.9	0.04
TNFRSF1A	-0.87	<0.01	-1	<0.01

GABRQ	-0.88	<0.01	-0.9	0.04
SERPINE1	-0.88	<0.01	-1	<0.01
TYK2	-0.9	<0.01	-0.9	0.04
TLR2	-0.92	<0.01	-0.9	0.04
ITGA2B	-0.93	<0.01	-0.9	0.04
SLC1A3	-0.93	<0.01	-1	<0.01

P value indicates the significance test for spearman correlation.

Supplementary table 8. The interaction test between the PD-L1 and candidate genes using atezolizumab-treated patients from OAK trial.

Gene	Source (Gene set)	Overall survival				Progression-free survival			
		coef	se(coef)	z	Pr(>|z|)	coef	se(coef)	z	Pr(>|z|)
CDKN1C	CSR_Activated_15701700	0.65	0.26	2.50	0.01	0.53	0.23	2.28	0.02
TRIM22	CSR_Activated_15701700	0.36	0.27	1.32	0.19	0.32	0.23	1.37	0.17
MBP	CSR_Activated_15701700	0.33	0.26	1.23	0.22	0.22	0.23	0.97	0.33
BCL2L14	Rotterdam_ERneg_PCA_15721472	0.31	0.26	1.18	0.24	0.39	0.23	1.71	0.09
FAS	IFNG_score_21050467	0.30	0.28	1.07	0.28	0.32	0.24	1.32	0.19
CTNS	CSR_Activated_15701700	0.12	0.27	0.47	0.64	0.03	0.23	0.15	0.88
TNFRSF1A	TGFB_score_21050467	0.07	0.27	0.28	0.78	-0.02	0.23	-0.08	0.94
IFI44	STAT1_score, Interferon_19272155	0.07	0.28	0.25	0.80	0.13	0.24	0.56	0.58
CDKN1A	CSR_Activated_15701700	0.04	0.26	0.15	0.88	-0.22	0.23	-0.97	0.33
ITGA2B	KEGG_HEMATOPOIETIC_CELL_LINEAGE	-0.01	0.26	-0.05	0.96	-0.22	0.23	-0.95	0.34
CNTNAP1	CSR_Activated_15701700	-0.03	0.26	-0.10	0.92	0.09	0.23	0.41	0.68
GABARAPL1	CSR_Activated_15701700	-0.07	0.26	-0.25	0.80	-0.23	0.23	-0.99	0.32
LRP1	CSR_Activated_15701700, TGFB_score_21050467	-0.08	0.26	-0.31	0.76	-0.31	0.23	-1.37	0.17
CXCL11	STAT1_score, Chemokine12_score, TAMsurr_score, TAMsurr_TcClassII_ratio	-0.11	0.28	-0.38	0.71	0.09	0.24	0.37	0.71
MT1F	CSR_Activated_15701700, CHANG_CORE_SERUM_RESPONSE_UP	-0.12	0.26	-0.45	0.65	0.16	0.23	0.71	0.48
MAF	CSR_Activated_15701700	-0.12	0.27	-0.45	0.65	-0.13	0.23	-0.57	0.57
MSN	CSR_Activated_15701700, CHANG_CORE_SERUM_RESPONSE_UP, CSF1_response	-0.12	0.27	-0.46	0.65	0.01	0.24	0.05	0.96
SERPINE1	TGFB_score_21050467	-0.15	0.26	-0.57	0.57	-0.28	0.23	-1.24	0.22
TYK2	IL13_score_21050467	-0.16	0.26	-0.59	0.55	-0.08	0.23	-0.37	0.71
TLR2	CSF1_response	-0.16	0.27	-0.61	0.54	-0.15	0.23	-0.66	0.51
ADAMDEC1	STAT1_score	-0.16	0.26	-0.62	0.54	0.25	0.23	1.08	0.28
GABRQ	Rotterdam_ERneg_PCA_15721472	-0.17	0.26	-0.64	0.52	0.13	0.23	0.58	0.56
SMO	TGFB_score_21050467	-0.17	0.26	-0.66	0.51	-0.01	0.23	-0.05	0.96

CORO1C	CSR_Activated_15701700, CHANG_CORE_SERUM_RESPONSE_UP	-0.25	0.27	-0.95	0.34	-0.25	0.24	-1.07	0.28
IFIT3	STAT1_score, IFNG_score_21050467	-0.30	0.28	-1.07	0.28	0.00	0.24	-0.01	1.00
UBE2L6	Interferon_Cluster_21214954	-0.40	0.27	-1.46	0.14	0.11	0.24	0.46	0.65
SLC1A3	CSF1_response	-0.39	0.26	-1.48	0.14	-0.08	0.23	-0.36	0.72
RHOQ	TGFB_score_21050467	-0.40	0.26	-1.53	0.13	0.07	0.23	0.30	0.76
DCBLD2	CHANG_CORE_SERUM_RESPONSE_UP	-0.45	0.27	-1.68	0.09	-0.26	0.23	-1.12	0.26
FADS1	CSR_Activated_15701700	-0.60	0.26	-2.31	0.02	-0.22	0.23	-0.96	0.34

MATN1 was significantly correlated with OS and PFS PD-L1 predictiveness but was not analyzed here because patients could not be grouped into two groups based on the median value of MATN1.

**Supplementary table 9.** Baseline characteristics of the OAK trial according to PD-L1 gene expression within Predictiveness-High and Predictiveness-Low groups.						
	**Predictiveness-High**			**Predictiveness-Low**		
	**PD-L1 Low**	**PD-L1 High**	***P* value**	**PD-L1 Low**	**PD-L1 High**	***P* value**
**Total**	157	192		192	158	
**Histology (%)**			0.194			0.445
Non-squamosus	111 (70.7)	122 (63.5)		148 (77.1)	128 (81)	
Squamous	46 (29.3)	70 (36.5)		44 (22.9)	30 (19.0)	
**Arm (%)**			0.288			1
Atezolizumab	67 (42.7)	94 (49.0)		100 (52.1)	83 (52.5)	
Docetaxel	90 (57.3)	98 (51.0)		92 (47.9)	75 (47.5)	
**Gender (%)**			0.265			0.716
Male	108 (68.8)	120 (62.5)		119 (62.0)	94 (59.5)	
Female	49 (31.2)	72 (37.5)		73 (38)	64 (40.5)	
**PD-L1 IHC expression (%)**			**<0.001**			**<0.001**
[0,1)	69 (43.9)	19 (9.9)		71 (37.0)	18 (11.4)	
[1,50)	25 (15.9)	34 (17.7)		20 (10.4)	26 (16.5)	
[50,100]	1 (0.6)	50 (26.0)		1 (0.5)	27 (17.1)	
Unknown	62 (39.5)	89 (46.4)		100 (52.1)	87 (55.1)	
**TMB (%)**			0.592			**0.019**
<16	69 (43.9)	89 (46.4)		103 (53.6)	62 (39.2)	
>=16	32 (20.4)	31 (16.1)		21 (10.9)	18 (11.4)	
Unknown	56 (35.7)	72 (37.5)		68 (35.4)	78 (49.4)	
**STK11 status (%)**			**0.003**			**0.001**
MUT	27 (17.2)	12 (6.2)		51 (26.6)	16 (10.1)	
WT	88 (56.1)	133 (69.3)		94 (49.0)	96 (60.8)	
Unknown	42 (26.8)	47 (24.5)		47 (24.5)	46 (29.1)	
**KEAP1 status (%)**			0.343			0.449
MUT	26 (16.6)	23 (12.0)		29 (15.1)	18 (11.4)	
WT	89 (56.7)	122 (63.5)		116 (60.4)	94 (59.5)	

Unknown	42 (26.8)	47 (24.5)		47 (24.5)	46 (29.1)	
**EGFR status (%)**			0.518			0.304
MUT	14 (8.9)	12 (6.2)		17 (8.9)	19 (12.0)	
WT	101 (64.3)	133 (69.3)		128 (66.7)	93 (58.9)	
Unknown	42 (26.8)	47 (24.5)		47 (24.5)	46 (29.1)	

P value indicates χ2 test. The cutoffs of PD-L1 predictiveness score and PD-L1 gene expression were their median values of total intention-to-treat patients. Abbreviations: IHC, immunohistochemistry; TMB, tumor mutation burden; MUT, mutant; WT, wild-type.

Supplementary table 10. Baseline characteristics of the POPLAR trial according to PD-L1 gene expression within Predictiveness-High and Predictiveness-Low groups.

	**Predictiveness-High**			**Predictiveness-Low**		
	**PD-L1 Low**	**PD-L1 High**	***P* value**	**PD-L1 Low**	**PD-L1 High**	***P* value**
**Total**	38	58		58	38	
**Histology (%)**			0.827			0.358
Non-squamosus	22 (57.9)	31 (53.4)		43 (74.1)	24 (63.2)	
Squamous	16 (42.1)	27 (46.6)		15 (25.9)	14 (36.8)	
**Arm (%)**			0.121			0.583
Atezolizumab	24 (63.2)	26 (44.8)		29 (50.0)	16 (42.1)	
Docetaxel	14 (36.8)	32 (55.2)		29 (50.0)	22 (57.9)	
**Gender (%)**			0.848			0.14
Male	24 (63.2)	39 (67.2)		39 (67.2)	19 (50.0)	
Female	14 (36.8)	19 (32.8)		19 (32.8)	19 (50.0)	
**PD-L1 IHC expression (%)**			**<0.001**			**<0.001**
[0,1)	24 (63.2)	18 (31.0)		40 (69.0)	9 (23.7)	
[1,50)	6 (15.8)	20 (34.5)		6 (10.3)	12 (31.6)	
[50,100]	0 (0.0)	16 (27.6)		1 (1.7)	3 (7.9)	
Unknown	8 (21.1)	4 (6.9)		11 (19.0)	14 (36.8)	

P value indicates $\chi2$ test. The cutoffs of PD-L1 predictiveness score and PD-L1 gene expression were their median values of total intention-to-treat patients. Abbreviations: IHC, immunohistochemistry.

Supplementary table 11. Baseline characteristics of the IMvigor210 trial according to PD-L1 gene expression within Predictiveness-High and Predictiveness-Low groups.

	Predictiveness-High			Predictiveness-Low		
	**PD-L1 Low**	**PD-L1 High**	***P* value**	**PD-L1 Low**	**PD-L1 High**	***P* value**
**Total**	98	76		76	98	
**Gender (%)**			0.353			0.617
Male	73 (74.5)	62 (81.6)		58 (76.3)	79 (80.6)	
Female	25 (25.5)	14 (18.4)				
**Race (%)**			0.796			0.73
White	88 (89.8)	70 (92.1)		71 (93.4)	89 (90.8)	
Other	10 (10.2)	6 (7.9)		5 (6.6)	9 (9.2)	
**ECOG (%)**			0.228			0.591
0	29 (29.6)	30 (39.5)		35 (46.1)	30 (40.8)	
1 and 2	69 (70.4)	46 (60.5)		41 (53.9)	58 (59.2)	
**Lund molecular subtype (%)**			**<0.001**			**<0.001**
UroA	61 (62.2)	7 (9.2)		29 (38.2)	5 (5.1)	
Genomically unstable	11 (11.2)	14 (18.4)		21 (27.6)	24 (24.5)	
Infiltrated	9 (9.2)	21 (27.6)		20 (26.3)	42 (42.9)	
UroB	7 (7.1)	6 (7.9)		2 (2.6)	3 (3.1)	
Basal/SCC-like	10 (10.2)	28 (36.8)		4 (5.3)	24 (24.5)	
**PD-L1 IHC expression (%)**			**<0.001**			**<0.001**
[0,1)	92 (93.9)	45 (59.2)		70 (92.1)	68 (69.4)	
[1,5)	4 (4.1)	6 (7.9)		5 (6.6)	7 (7.1)	
[5,100]	2 (2.0)	25 (32.9)		0 (0.0)	23 (23.5)	
Unknown	0 (0)	0 (0)		1 (1.3)	0 (0.0)	
**TMB (%)**			0.174			0.431
<16	71 (72.4)	45 (59.2)		50 (65.8)	55 (56.1)	
>=16	11 (11.2)	14 (18.4)		10 (13.2)	16 (16.3)	
Unknown	16 (16.3)	17 (22.4)		16 (21.1)	27 (27.6)	

**TNB (Median, IQR)**	0.78 (0.43-1.41)	0.98 (0.59-1.98)	0.09	0.75 (0.51-1.35)	1.07 (0.46-1.91)	0.47

Categorical and continuous variables were compared by the $\chi 2$ test and Wilcoxon rank sum test, respectively. The cutoffs of PD-L1 predictiveness score and PD-L1 gene expression were their median values of total intention-to-treat patients. The median and interquartile range values of tumor neoantigen burden was calculated after removing unknown values. Abbreviations: EGOG, Eastern Cooperative Oncology Group; IHC, immunohistochemistry; TMB, tumor mutation burden; TNB, tumor neoantigen burden; IQR, interquartile range.

Supplementary table 12. The interaction test between the PD-L1 and CDKN1C using atezolizumab-treated patients from POPLAR and IMvigor210 trials.

	coef	se(coef)	z	Pr(>|z|)
**POPLAR OS**	1.41	0.46	3.09	0.002
**POPLAR PFS**	1.43	0.44	3.27	0.001
**IMvigor210 OS**	0.70	0.27	2.57	0.01

Abbreviations: OS, overall survival; PFS, progression-free survival.

Supplementary table 13. Distribution of immune subtype according to PD-L1 gene expression within Predictiveness-High and Predictiveness-Low groups.

	**Predictiveness-High**			**Predictiveness-Low**		
	**PD-L1 Low**	**PD-L1 High**	***P* value**	**PD-L1 Low**	**PD-L1 High**	***P* value**
**OAK**						
**Total**	157	192	**3.00E-05**	192	158	**0.008**
Immune-Enriched Subtype (%)	62 (39.5)	120 (62.5)		68 (35.4)	79 (50)	
Non-Immune Subtype (%)	95 (60.5)	72 (37.5)		124 (64.6)	79 (50)	
**POPLAR**						
**Total**	38	58	**0.01**	58	38	**0.02**
Immune-Enriched Subtype (%)	14 (36.8)	38 (65.5)		17 (29.3)	21 (55.2)	
Non-Immune Subtype (%)	24 (63.2)	20 (34.5)		41 (70.7)	17 (44.8)	
**IMvigor210**						
**Total**	98	76	**2.59E-13**	76	98	**3.44E-05**
Immune-Enriched Subtype (%)	17 (17.3)	56 (73.7)		34 (44.7)	75 (76.5)	
Non-Immune Subtype (%)	81 (82.7)	20 (26.3)		42 (55.3)	23 (23.5)	

P value indicates $\chi 2$ test. The cutoffs of PD-L1 predictiveness score and PD-L1 gene expression were their median values of total intention-to-treat patients in each trial.

Supplementary table 14. Gene signatures used in this study.

Gene signature	Number	Gene	Source
Interferon-γ signature	6	CXCL10, CXCL9, HLA-DRA, IDO1, IFNG, STAT1	Ayers et al. 2017 PMID: 28650338
T-cell inflamed gene expression profile	18	CCL5, CD27, CD274, CD276, CD8A, CMKLR1, CXCL9, CXCR6, HLA-DQA1, HLA-DRB1, HLA-E, IDO1, LAG3, NKG7, PDCD1LG2, PSMB10, STAT1	Ayers et al. 2017 PMID: 28650338
CD8 score	2	CD8A, CD8B	Jiang et al PMID: 30127393
GOBP_INTERFERON_GAMMA_MEDIATED_SIGNALING_PATHWAY	100	ADAR,ARG1,AZI2,CACTIN,CDC37,CNOT7,DCST1,FADD,HCK,HPX,IFI27,IFITM1,IFITM2,IFITM3,IFNA1,IFNA10,IFNA13,IFNA14,IFNA16,IFNA17,IFNA2,IFNA21,IFNA4,IFNA5,IFNA6,IFNA7,IFNA8,IFNAR1,IFNAR2,IFNB1,IFNE,IFNG,IFNGR1,IFNGR2,IFNK,IFNL1,IFNL2,IFNL3,IFNL4,IFNLR1,IFNW1,IKBKE,IL10RB,IRAK1,IRF1,IRF3,IRF7,IRGM,ISG15,JAK1,JAK2,LSM14A,MAVS,MED1,METTL3,MIR21,MMP12,MUL1,MYD88,NLRC5,NR1H2,NR1H3,OAS1,OAS2,OAS3,OTOP1,PARP14,PARP9,PPARG,PTPN1,PTPN11,PTPN2,PTPN6,RBM47,RNF185,SAMHD1,SP100,STAT1,STAT2,STING1,TANK,TBK1,TBKBP1,TP53,TRAF3,TREX1,TRIM41,TRIM56,TRIM6,TTLL12,TXK,TYK2,UBE2K,USP18,USP27X,USP29,WNT5A,YTHDF2,YTHDF3,ZBP1	MSigDB

GOBP_POSITIVE_REGULATION_OF_INTERFERON_GAMMA_PRODUCTION	77	ABL1,ARID5A,BCL3,BTN3A1,BTN3A2,CCR2,CD14,CD160,CD2,CD226,CD244,CD276,CD3E,CEBPG,CLEC7A,CRTAM,CYRIB,EBI3,F2RL1,FADD,FZD5,HAVCR2,HLA-A, HLA-DPA1, HLA-DPB1, HMHB1, HRAS, HSPD1, IFNL1, IL12A,IL12B,IL12RB1,IL12RB2,IL18,IL18R1,IL1B,IL1R1, IL2,IL21,IL23A,IL23R,IL27,IL27RA,IRF8,ISG15,ISL1,JAK2,KLRC4-KLRK1, KLRK1, LGALS9, LILRB1, LTA, PDE4B, PDE4D, PTPN22, PYCARD, RASGRP1, RIPK2, SASH3,SCRIB,SLAMF1,SLAMF6,SLC11A1,SLC7A5,TLR3,TLR4,TLR7,TLR8,TLR9,TNF,TNFSF4,TRIM27,TXK,TYK2,WNT5A,ZFPM1,ZP3	MSigDB
GOBP_INTERFERON_ALPHA_PRODUCTION	29	CHUK,DDX3X,DHX36,DHX9,HAVCR2,HSPD1,IFIH1,IL10,IRF3,IRF5,IRF7,LILRA4,MAVS,MMP12,NLRC3,NMB, NMBR,NMI,PTPRS,RIGI,RIPK2,SETD2,STAT1,TBK1,TLR3,TLR4,TLR7,TLR8,TLR9	MSigDB
GOBP_CELLULAR_RESPONSE_TO_INTERFERON_BETA	32	ACOD1,AIM2,BST2,CAMK2A,CAPN2,CDC34,HTRA2,IFI16,IFITM1,IFITM2,IFITM3,IFNAR2,IFNB1,IKBKE,IRF1,IRGM,MNDA,NDUFA13,OAS1,PLSCR1,PNPT1,PYDC5,PYHIN1,SHFL,STAT1,STING1,TLR3,TREX1,TRIM6,UBE2G2,UBE2K,XAF1	MSigDB
GOBP_POSITIVE_REGULATION_OF_ADAPTIVE_IMMUNE_RESPONSE	129	ADA,AKIRIN2,ARID5A,ATAD5,B2M,BTK,C17orf99,C3, CARD9,CCR2,CD1A,CD1B,CD1C,CD1D,CD1E,CD226,CD274,CD28,CD4,CD40,CD55,CD81,CLCF1,CLEC6A,CLEC7A,CYRIB,DENND1B,EIF2AK4,EXOSC3,EXOSC6,FADD,FBXO38,FCER2,FCGR1A,FOXP3,FZD5,GATA3,HFE,HLA-A,HLA-B,HLA-C,HLA-DRA,HLA-DRB1,HLA-DRB3,HLA-E,HLA-F,HLA-G,HLA-H,HMCES,HPX,HSPD1,IL12A,IL12B,IL12RB1,IL18,IL18R1,IL1B,IL1R1,IL2,IL23A,IL23R,IL27RA,IL4,IL6,IL6ST,J	MSigDB

		AK2,KMT5B,KMT5C,LTA,MAD2L2,MALT1,MAP3K7,MICA,MICB,MIR21,MLH1,MR1,MSH2,NECTIN2,NFKBID,NFKBIZ,NLRP10,NLRP3,NOD2,NSD2,P2RX7,PAXIP1,PLA2G4A,PRKCQ,PRKCZ,PTPRC,PVR,PYCARD,RAET1E,RAET1G,RAET1L,RIF1,RIPK2,RSAD2,SASH3,SHLD1,SHLD2,SHLD3,SIRT1,SKAP1,SLAMF1,SLC11A1,SLC22A13,STAT6,STX7,TAP2,TBX21,TFRC,TGFB1,TNF,TNFSF13,TNFSF13B,TNFSF4,TP53BP1,TRAF2,TRAF6,TREM2,TYK2,ULBP1,ULBP2,ULBP3,XCL1,ZBTB1,ZP3	
GOBP_T_CELL_CHEMOTAXIS	27	ADAM10,ADAM17,CCL21,CCL26,CCL3,CCL5,CCR2,CXCL10,CXCL11,CXCL13,CXCL16,DEFA1,DEFA1B,GPR183,OXSR1,PIK3CD,PIK3CG,PLEC,S100A7,SLC12A2,STK39,TMEM102,TNFSF14,WNK1,WNT5A,XCL1,XCL2	MSigDB
GOBP_RECEPTOR_SIGNALING_PATHWAY_VIA_STAT	185	ADIPOR1,AGT,ARL2BP,BCL3,CAMK2A,CAV1,CCL2,CCL5,CCR2,CD300A,CD40,CDK5,CDK5R1,CENPJ,CLCF1,CLEC12B,CNOT7,CNTF,CRLF1,CRLF2,CRLF3,CSF1R,CSF2,CSF2RA,CSF2RB,CSH1,CSH2,CSHL1,CTF1,CTR9,CYP1B1,DAB1,DOT1L,EGF,ELP2,EPHB2,EPO,ERBB4,F2,F2R,FER,FGFR3,FLT3,FYN,GADD45A,GBP7,GGNBP2,GH1,GH2,GHR,HAMP,HCLS1,HDAC2,HES1,HES5,HGS,HPX,HSF1,IFNA1,IFNA10,IFNA13,IFNA14,IFNA16,IFNA17,IFNA2,IFNA21,IFNA4,IFNA5,IFNA6,IFNA7,IFNA8,IFNAR1,IFNAR2,IFNB1,IFNE,IFNG,IFNK,IFNL1,IFNL2,IFNL3,IFNL4,IFNW1,IGF1,IL10,IL10RA,IL10RB,IL12A,IL12B,IL13,IL15,IL18,IL2,IL20,IL21,IL22RA2,IL23A,IL23R,IL24,IL26,IL3,IL31RA,IL4,IL5,IL6,IL6R,IL6ST,IL7R,IL9,INPP5F,ISL1,JAK1,JAK2,JAK3,KIT,LEP,LEPROT,LIF,LYN,MGAT5,MIR125A,MIR125B1,MIR146A,MIR149,MIR19A,MIR19B1,MIR221,MIR9-1, MIR99A, MIRLET7C, MIRLET7E, MST1, MST1L, NEUROD1, NF2, NLK, NMI,NOTCH1,OCIAD1,OCIAD2,OSBP,OSM,PARP14,PARP9,PIAS1,PIBF1,PIGU,PKD1,PKD2,PPARG,PPP2CA,PPP2R1A,PRL,PRLR,PTK2B,PTK6,PTPN2,PTPRC,PTPRD,P	MSigDB

		TPRT,PWP1,RET,SH2B3,SOCS1,SOCS2,SOCS3,SOCS5,SOCS6,STAMBP,STAT1,STAT2,STAT3,STAT4,STAT5A,STAT5B,STAT6,TGFB1,THPO,TNF,TNFRSF18,TNFRSF1A,TNFSF18,TSLP,TYK2,VEGFA,VHL	
GOBP_ACTIVATED_T_CELL_PROLIFERATION	50	ABL1,AGER,ARG1,BTN2A2,BTN3A1,CADM1,CASP3,CD24,CD274,CLC,CRTAM,EPO,FADD,FOXP3,FYN,GPAM,HHLA2,HMGB1,ICOSLG,IDO1,IGF1,IGF2,IGFBP2,IL12B,IL12RB1,IL18,IL2,IL23A,IL23R,IL2RA,JAK3,LGALS9,LILRB4,LRRC32,MIR181C,MIR21,MIR30B,PDCD1LG2,PPP3CA,PRKAR1A,PRNP,PYCARD,RC3H1,RIPK3,RPS3,SCRIB,SLAMF1,STAT5B,TMIGD2,TNFSF9	MSigDB
GOBP_REGULATION_OF_T_CELL_ACTIVATION	377	ABL1,ABL2,ACTB,ACTL6A,ACTL6B,ADA,ADAM8,ADORA2A,AGER,AIF1,AKT1,AMBRA1,ANXA1,AP3B1,AP3D1,ARG1,ARG2,ARID1A,ARID1B,ARID2,B2M,BAD,BATF,BCL10,BCL6,BID,BMI1,BMP4,BRAF,BRD7,BTN2A2,CAMK4,CARD11,CASP3,CAV1,CBFB,CBLB,CCDC88B,CCL19,CCL2,CCL21,CCL5,CCR2,CCR7,CD160,CD1D,CD2,CD209,CD24,CD27,CD274,CD276,CD28,CD300A,CD3E,CD4,CD40LG,CD46,CD47,CD5,CD55,CD6,CD70,CD74,CD80,CD81,CD83,CD86,CEACAM1,CEBPB,CGAS,CLC,CLEC4G,CLECL1P,CLPTM1,CORO1A,CR1,CRTAM,CSK,CTLA4,CTNNB1,CTSG,CYP26B1,CYRIB,DHPS,DLG1,DLG5,DNAJA3,DOCK8,DPP4,DROSHA,DTX1,DUSP10,DUSP22,DUSP3,EBI3,EFNB1,EFNB2,EFNB3,EGR3,EPO,ERBB2,FADD,FANCA,FANCD2,FCGR2B,FCHO1,FGL1,FGL2,FLOT2,FOXJ1,FOXN1,FOXO3,FOXP3,FYN,GATA3,GLI2,GLI3,GLMN,GPAM,GPNMB,HAVCR2,HES1,HFE,HHLA2,HLA-A,HLA-DMA,HLA-DMB,HLA-DOA,HLA-DOB,HLA-DPA1,HLA-DPB1,HLA-DQA1,HLA-DQA2,HLA-DQB1,HLA-DQB2,HLA-DRA,HLA-DRB1,HLA-DRB3,HLA-DRB4,HLA-DRB5,HLA-E,HLA-G,HLX,HMGB1,HSPD1,HSPH1,ICOS,ICOSLG,IDO1,IFNA2,IFNB1,IFNG,IFNL1,IGF1,IGF2,IGFBP2,IHH,IL10,IL12	MSigDB

		A,IL12B,IL12RB1,IL15,IL18,IL1A,IL1B,IL1RL2,IL2,IL20RB,IL21,IL23A,IL23R,IL27,IL2RA,IL2RG,IL36B,IL4,IL4I1,IL4R,IL6,IL6ST,IL7,IL7R,ILDR2,IRF1,IRF4,ITCH,ITPKB,JAK2,JAK3,JUNB,KAT2A,KAT5,KITLG,KLHL25,KLRC4,KLRK1,KLRK1,LAG3,LAPTM5,LAT,LAX1,LCK,LEF1,LEP,LGALS1,LGALS3,LGALS9,LGALS9B,LGALS9C,LILRB1,LILRB2,LILRB4,LMO1,LOXL3,LRRC32,LYN,MAD1L1,MALT1,MAP3K8,MAPK8IP1,MARCHF7,MDK,METTL3,MIR181C,MIR21,MIR27A,MIR30B,NCK1,NCK2,NCKAP1L,NDFIP1,NFKBID,NFKBIZ,NKAP,NLRP3,NOD2,NRARP,PAG1,PAWR,PBRM1,PCK1,PDCD1LG2,PDE5A,PELI1,PHF10,PIK3CA,PIK3R6,PLA2G2A,PLA2G2D,PLA2G2E,PLA2G2F,PLA2G5,PNP,PPP3CA,PRDM1,PRDX2,PRELID1,PRKAR1A,PRKCQ,PRKCZ,PRNP,PSG9,PTPN11,PTPN2,PTPN22,PTPN6,PTPRC,PYCARD,RAC2,RAG1,RAG2,RARA,RASAL3,RASGRP1,RC3H1,RC3H2,RHOA,RHOH,RIPK2,RIPK3,RIPOR2,RPS3,RUNX1,RUNX3,SART1,SASH3,SCGB1A1,SCRIB,SDC4,SELENOK,SFTPD,SH3RF1,SHB,SHH,SIRPA,SIRPB1,SIRPG,SIT1,SLAMF1,SLC46A2,SLC4A2,SLC7A1,SMAD7,SMARCA2,SMARCA4,SMARCB1,SMARCC1,SMARCC2,SMARCD1,SMARCD2,SMARCD3,SMARCE1,SOCS1,SOCS5,SOCS6,SOD1,SOS1,SOS2,SOX12,SOX13,SOX4,SPINK5,SPN,SPTA1,SRC,STAT5B,SYK,TARM1,TBX21,TCF7,TESPA1,TFRC,TGFBR2,THY1,TIGIT,TMEM131L,TMIGD2,TNFAIP8L2,TNFRSF13C,TNFRSF14,TNFRSF1B,TNFRSF21,TNFRSF9,TNFSF11,TNFSF13B,TNFSF14,TNFSF18,TNFSF4,TNFSF8,TNFSF9,TOX,TRAF6,TREX1,TSPAN32,TWSG1,TYK2,VAV1,VCAM1,VNN1,VSIG4,VSIR,VTCN1,WNT10B,XBP1,XCL1,YES1,ZAP70,ZBTB1,ZBTB16,ZBTB7B,ZC3H12A,ZC3H8,ZEB1,ZMIZ1,ZNF683,ZP3,ZP4	
GOBP_POSITIVE_REGULATION_OF_TRANSFORMING_GROWTH_FACTOR_BETA_PRODUCTION	22	ATF2,ATP6AP2,BMPR1A,CD200,CD34,CD46,CX3CL1,FERMT1,FOXP3,FURIN,GATA6,LGALS9,LUM,MIR149,P	MSigDB

		TGS2,SERPINB7,SERPINF2,SMAD3,THBS1,TNXB,WNT11,XCL1	
REACTOME_MTOR_SIGNALLING	41	AKT1,AKT1S1,AKT2,CAB39,CAB39L,EEF2K,EIF4B,EIF4E,EIF4EBP1,EIF4G1,FKBP1A,LAMTOR1,LAMTOR2,LAMTOR3,LAMTOR4,LAMTOR5,MLST8,MTOR,PPM1A,PRKAA1,PRKAA2,PRKAB1,PRKAB2,PRKAG1,PRKAG2,PRKAG3,RHEB,RPS6,RPS6KB1,RPTOR,RRAGA,RRAGB,RRAGC,RRAGD,SLC38A9,STK11,STRADA,STRADB,TSC1,TSC2,YWHAB	MSigDB
BILD_MYC_ONCOGENIC_SIGNATURE	210	ABCC3,ABL2,ACSS2,ADAM8,ADAMTS15,ADGRF1,AGPAT5,AHNAK,AIMP2,AKAP12,ALDH1B1,ALG3,ALS2CL,ANAPC1,ANGEL1,ATIC,ATP2B4,BCS1L,BEND3,BID,BMAL1,BMAL2,BOP1,C19orf48,C20orf27,CCDC137,CCDC78,CCDC86,CCNG2,CDCA8,CDK2,CDK4,CFLAR,CGN,CIART,COL1A1,COL5A1,COL8A2,CYTH2,DAB2,DCAF4,DDAH2,DDX10,DDX18,DDX28,DGKA,DHODH,DHRS1,DKC1,DPH1,DTX2P1-UPK3BP1-PMS2P11, DUOX1,DUSP2,DUSP22,EEF1AKMT4,EMP1,EPS8L1,ERBB3,ERCC1,ERVMER34-1, EXOSC5, FAM168A, FAM193B,FAM86B1,FAM86B3P,FAM86C1P,FARP1,FARSA,FARSB,FCHSD1,FGD6,FGF11,FGFBP1,FLAD1,FLNB,GEMIN5,GNL3,GOLGA2,GPATCH4,GPD1L,GPRC5A,H2AC6,HK2-DT, HS6ST2, HSPA1A, HSPA6, HSPD1, IMP4,INTS6L,IPO4,IRF9,KCNQ5,KHNYN,KICS2,KRTAP2-3,LAMC2,LBHD1,LINC00173,LSG1,LTBP2,MAP3K6,MCOLN2,MCRIP2,MEAK7,MEG3,METTL1,MICAL1,MKNK1,MON1A,MRTO4,MST1R,MTMR11,MYBBP1A,MYC,NCL,NCR3LG1,NEU3,NFKB2,NGFR,NIT1,NLE1,NLRP1,NOL6,NOLC1,NOP16,NOP2,NOP56,NPM1,NRP1,NTF4,NUFIP1,OR2A4,OSBP2,PA2G4,PAICS,PC,PCOLCE2,PCYOX1L,PDP1,PES1,PFKM,PHB1,PHF1,PLAU,PLD6,PLEC,PLEKHG2,PLEKHG3,PMM2,PNPT1,PRR5,PTRH2,RABEPK,RCL1,RHBDF1,RNF213,RPP40,RRP9,RUNX1,SCFD2,SERP	MSigDB

		INB1,SESTD1,SFXN4,SLC12A8,SLC19A1,SLC29A2,SLC6A15,SLC6A8,SLITRK6,SMTN,SMURF1,SNHG16,SNORD22,SORD,SORL1,SPTAN1,SRM,SSH3,STMN3,SUPV3L1,TAF4B,TCOF1,TFB2M,TGM1,TGM2,TINF2,TMEM97,TNFAIP2,TRIOBP,TRMT1,TTLL12,TUBA1A,TWNK,UBALD1,UBE2D4,UBIAD1,UTP14A,UTP14C,UTP20,UTP25,UTP4,VAMP1,VMP1,WDR12,WDR74,WSB1,XPO5,YPEL3,ZBED2,ZNF667	
REACTOME_PTEN_REGULATION	139	AGO1,AGO2,AGO3,AGO4,AKT1,AKT2,AKT3,ATF2,ATN1,BMI1,CBX2,CBX4,CBX6,CBX8,CHD3,CHD4,CSNK2A1,CSNK2A2,CSNK2B,EED,EGR1,EZH2,FRK,GATAD2A,GATAD2B,HDAC1,HDAC2,HDAC3,HDAC5,HDAC7,JUN,KDM1A,LAMTOR1,LAMTOR2,LAMTOR3,LAMTOR4,LAMTOR5,MAF1,MAPK1,MAPK3,MBD3,MECOM,MKRN1,MLST8,MOV10,MTA1,MTA2,MTA3,MTOR,NEDD4,NR2E1,OTUD3,PHC1,PHC2,PHC3,PML,PPARG,PREX2,PSMA1,PSMA2,PSMA3,PSMA4,PSMA5,PSMA6,PSMA7,PSMA8,PSMB1,PSMB10,PSMB11,PSMB2,PSMB3,PSMB4,PSMB5,PSMB6,PSMB7,PSMB8,PSMB9,PSMC1,PSMC2,PSMC3,PSMC4,PSMC5,PSMC6,PSMD1,PSMD10,PSMD11,PSMD12,PSMD13,PSMD14,PSMD2,PSMD3,PSMD4,PSMD5,PSMD6,PSMD7,PSMD8,PSMD9,PSME1,PSME2,PSME3,PSME4,PSMF1,PTEN,RBBP4,RBBP7,RCOR1,REST,RHEB,RING1,RNF146,RNF2,RPS27A,RPTOR,RRAGA,RRAGB,RRAGC,RRAGD,SALL4,SCMH1,SEM1,SLC38A9,SNAI1,SNAI2,STUB1,SUZ12,TNKS,TNKS2,TNRC6A,TNRC6B,TNRC6C,TP53,TRIM27,UBA52,UBB,UBC,USP13,USP7,WWP2,XIAP	MSigDB
PID_MET_PATHWAY	79	AKT1,AKT1S1,AKT2,APC,ARF6,ARHGEF4,BAD,BCAR1,CBL,CDC42,CDH1,CRK,CRKL,CTNNA1,CTNNB1,DEPTOR,EGR1,EIF4E,EIF4EBP1,EPS15,ETS1,F2RL2,GAB1,GAB2,GRB2,HGF,HGS,HRAS,INPPL1,JUN,KPNB1,MAP2K1,MAP2K2,MAP2K4,MAP3K1,MAPK1,MAPK3,MAPK8,M	MSigDB

		ET,MLST8,MTOR,MUC20,NCK1,NCK2,NUMB,PAK1,PAK2,PAK4,PARD6A,PDPK1,PIK3CA,PIK3R1,PLCG1,PRKCI,PRKCZ,PTK2,PTPN1,PTPN11,PTPN2,PTPRJ,PXN,RAB5A,RAC1,RAF1,RANBP10,RANBP9,RAP1A,RAP1B,RAPGEF1,RHOA,RIN2,RPTOR,SH3GL2,SH3KBP1,SHC1,SNAI1,SOS1,SRC,WASL	
REACTOME_PI3K_AKT_ACTIVATION	9	IRS1,IRS2,NGF,NTRK1,PIK3CA,PIK3CB,PIK3R1,PIK3R2,RHOA	MSigDB
REACTOME_ONCOGENIC_MAPK_SIGNALING	82	ACTB,ACTG1,AGGF1,AGK,AGTRAP,AKAP9,AP3B1,APBB1IP,ARAF,ARRB1,ARRB2,ATG7,BCL2L11,BRAF,BRAP,CALM1,CAMK2A,CAMK2B,CAMK2D,CAMK2G,CLCN6,CNKSR1,CNKSR2,CSK,DUSP10,DUSP16,DUSP6,DUSP7,DUSP8,DUSP9,ESRP1,FAM114A2,FAM131B,FGA,FGB,FGG,FN1,FXR1,HRAS,IQGAP1,ITGA2B,ITGB3,JAK2,KDM7A,KIAA1549,KRAS,KSR1,KSR2,LMNA,MAP2K1,MAP2K2,MAP3K11,MAPK1,MAPK3,MARK3,MPRIP,MRAS,NF1,NRAS,PAPSS1,PEBP1,PHB1,PPP1CB,PPP1CC,QKI,RAF1,RAP1A,RAP1B,SHOC2,SND1,SPRED1,SPRED2,SPRED3,SRC,TENT4A,TLN1,TRAK1,TRIM24,VCL,VWF,YWHAB,ZC3HAV1	MSigDB
REACTOME_SIGNALING_BY_EGFR	50	AAMP,ADAM10,ADAM12,ADAM17,AREG,ARHGEF7,BTC,CBL,CDC42,CSK,EGF,EGFR,EPGN,EPN1,EPS15,EPS15L1,EREG,GAB1,GRB2,HBEGF,HGS,HRAS,KRAS,LRIG1,NRAS,PAG1,PIK3CA,PIK3R1,PLCG1,PTPN11,PTPN12,PTPN3,PTPRK,PXN,RPS27A,SH3GL1,SH3GL2,SH3GL3,SH3KBP1,SHC1,SOS1,SPRY1,SPRY2,SRC,STAM,STAM2,TGFA,UBA52,UBB,UBC	MSigDB
BIOCARTA_CDK5_PATHWAY	13	CDK5,CDK5R1,DPM2,EGR1,HRAS,KLK2,MAP2K1,MAP2K2,MAPK1,MAPK3,NGF,NGFR,RAF1	MSigDB
GOMF_EXTRACELLULAR_MATRIX_BINDING	55	ACHE,ADAM9,ADAMTS15,ADAMTS5,ADGRG1,ADGRG6,AGRN,BCAM,BGN,CCN1,CD248,CLEC14A,COL11A1,CTSS,DAG1,DCN,DMP1,ECM1,ELN,FBLN2,GPC1,ITGA2,ITGA2B,ITGA3,ITGA7,ITGAV,ITGB1,ITGB3,LACRT,	MSigDB

		LGALS1,LGALS3,LRRC15,LYPD3,NID1,NTN4,OLFML2A,OLFML2B,PLEKHA2,PXDN,RPSA,SHH,SLIT2,SMOC1,SMOC2,SPARC,SPARCL1,SPOCK2,SPP1,SSC5D,TGFBI,THBS1,THSD1,TINAGL1,VEGFA,VTN	
GOMF_EXTRACELLULAR_MATRIX_STRUCTURAL_CONSTITUENT_CONFERRING_TENSILE_STRENGTH	41	COL10A1,COL11A1,COL11A2,COL12A1,COL13A1,COL14A1,COL15A1,COL16A1,COL17A1,COL18A1,COL19A1,COL1A1,COL1A2,COL21A1,COL23A1,COL24A1,COL25A1,COL27A1,COL28A1,COL2A1,COL3A1,COL4A1,COL4A2,COL4A3,COL4A4,COL4A5,COL4A6,COL5A1,COL5A2,COL5A3,COL6A1,COL6A2,COL6A3,COL6A5,COL6A6,COL7A1,COL8A1,COL8A2,COL9A1,COL9A2,COL9A3	MSigDB
GOBP_REGULATION_OF_EXTRACELLULAR_MATRIX_ASSEMBLY	17	AGT,ANTXR1,CLASP1,CLASP2,DAG1,EMILIN1,HAS2,MAD2L2,MIR29B1,NOTCH1,PHLDB1,PHLDB2,RGCC,SMAD3,SOX9,TGFB1,TIE1	MSigDB
GOMF_EXTRACELLULAR_MATRIX_STRUCTURAL_CONSTITUENT_CONFERRING_COMPRESSION_RESISTANCE	22	ACAN,AMBN,AMELX,AMELY,ASPN,BGN,CHADL,DCN,ENAM,FMOD,HAPLN1,HSPG2,LUM,OGN,PODN,PRELP,PRG2,PRG3,PRG4,STATH,TUFT1,VCAN	MSigDB
REACTOME_CELL_EXTRACELLULAR_MATRIX_INTERACTIONS	18	ACTB,ACTG1,ACTN1,ARHGEF6,FBLIM1,FERMT2,FLNA,FLNC,ILK,ITGB1,LIMS1,LIMS2,PARVA,PARVB,PXN,RSU1,TESK1,VASP	MSigDB
GOBP_POSITIVE_REGULATION_OF_COLLAGEN_METABOLIC_PROCESS	28	AMELX,ARRB2,BMP4,CCN2,CREB3L1,DDR2,ENG,F2,F2R,HDAC2,IHH,INHBA,ITGA2,LARP6,MIR149,PDGFRB,RGCC,RUNX1,SCX,SERPINB7,SERPINF2,SUCO,TGFB1,TGFB3,UCN,VIM,VSIR,WNT4	MSigDB
GOMF_COLLAGEN_BINDING_INVOLVED_IN_CELL_MATRIX_ADHESION	5	ITGA1,ITGA10,ITGA11,ITGA2,ITGB1	MSigDB
GOBP_MESENCHYMAL_CELL_PROLIFERATION	43	BMP2,BMP4,BMP7,BMPR1A,CHRD,CTNNB1,CTNNBIP1,DCHS1,DCHS2,FGF4,FGF7,FGF9,FGFR2,FOXF1,GPC3,HAND2,IHH,IRS2,KDR,LMNA,LRP5,MSX1,MYC,MYCN,NFIB,OSR1,PDGFA,PHF14,PRRX1,SHH,SHOX2,SIX1,SIX2,SMO,SOX9,STAT1,TBX1,TBX2,TGFBR2,WNT11,WNT2,WNT5A,ZEB1	MSigDB

GOMF_FILAMIN_BINDING	14	CEACAM1,CRMP1,DPYSL3,DPYSL4,FBLIM1,HSPB7,MICALL2,NEBL,PDLIM2,RFLNA,RFLNB,SMAD4,SYNPO2,TMEM67	MSigDB
GOBP_REGULATION_OF_LENS_FIBER_CELL_DIFFERENTIATION	9	CDKN1B,CDKN1C,FGF2,FOXE3,SPRED1,SPRED2,SPRED3,SPRY1,SPRY2	MSigDB
GOBP_MESENCHYME_MIGRATION	5	ACTA1,ACTA2,ACTC1,ACTG2,FOXF1	MSigDB
NABA_ECM_GLYCOPROTEINS	196	ABI3BP,ADIPOQ,AEBP1,AGRN,AMBN,AMELX,AMELY,ANOS1,BGLAP,BMPER,BSPH1,CCN1,CCN2,CCN3,CCN4,CCN5,CCN6,CDCP2,CILP,CILP2,COCH,COLQ,COMP,CRELD1,CRELD2,CRIM1,CRISPLD1,CRISPLD2,CTHRC1,DMBT1,DMP1,DPT,DSPP,ECM1,ECM2,EDIL3,EFEMP1,EFEMP2,EGFLAM,ELN,ELSPBP1,EMID1,EMILIN1,EMILIN2,EMILIN3,EYS,FBLN1,FBLN2,FBLN5,FBLN7,FBN1,FBN2,FBN3,FGA,FGB,FGG,FGL1,FGL2,FN1,FNDC1,FNDC7,FNDC8,FRAS1,GAS6,GLDN,HMCN1,HMCN2,IBSP,IGFALS,IGFBP1,IGFBP2,IGFBP3,IGFBP4,IGFBP5,IGFBP6,IGFBP7,IGFBPL1,IGSF10,INTS14,INTS6L,KCP,LAMA1,LAMA2,LAMA3,LAMA4,LAMA5,LAMB1,LAMB2,LAMB3,LAMB4,LAMC1,LAMC2,LAMC3,LGI1,LGI2,LGI3,LGI4,LRG1,LTBP1,LTBP2,LTBP3,LTBP4,MATN1,MATN2,MATN3,MATN4,MEPE,MFAP1,MFAP2,MFAP3,MFAP4,MFAP5,MFGE8,MGP,MMRN1,MMRN2,MXRA5,NDNF,NELL1,NELL2,NID1,NID2,NPNT,NTN1,NTN3,NTN4,NTN5,NTNG1,NTNG2,OIT3,OTOG,OTOL1,PAPLN,PCOLCE,PCOLCE2,POMZP3,POSTN,PXDN,PXDNL,RELN,RSPO1,RSPO2,RSPO3,RSPO4,SBSPON,SLIT1,SLIT2,SLIT3,SMOC1,SMOC2,SNED1,SPARC,SPARCL1,SPON1,SPON2,SPP1,SRPX,SRPX2,SSPOP,SVEP1,TECTA,TECTB,TGFBI,THBS1,THBS2,THBS3,THBS4,THSD4,TINAG,TINAGL1,TNC,TNFAIP6,TNN,TNR,TNXB,TSKU,TSPEAR,USH2A,VIT,VTN,VWA1,VWA2,VWA3A,VWA3B,VWA5A,VWA5B1,	MSigDB

		VWA5B2,VWA7,VWCE,VWDE,VWF,ZP1,ZP2,ZP3,ZP4,ZPLD1	
REACTOME_ECM_PROTEOGLYCANS	76	ACAN,AGRN,APP,ASPN,BCAN,BGN,COL1A1,COL1A2,COL2A1,COL3A1,COL4A1,COL4A2,COL4A3,COL4A4,COL4A5,COL4A6,COL5A1,COL5A2,COL5A3,COL6A1,COL6A2,COL6A3,COL6A5,COL6A6,COL9A1,COL9A2,COL9A3,COMP,DAG1,DCN,DMP1,DSPP,FMOD,FN1,HAPLN1,HSPG2,IBSP,ITGA2,ITGA2B,ITGA7,ITGA8,ITGA9,ITGAV,ITGAX,ITGB1,ITGB3,ITGB5,ITGB6,LAMA1,LAMA2,LAMA3,LAMA4,LAMA5,LAMB1,LAMB2,LAMC1,LRP4,LUM,MATN1,MATN3,MATN4,MUSK,NCAM1,NCAN,PTPRS,SERPINE1,SPARC,TGFB1,TGFB2,TGFB3,TNC,TNN,TNR,TNXB,VCAN,VTN	MSigDB
REACTOME_CROSSLINKING_OF_COLLAGEN_FIBRILS	18	BMP1,COL1A1,COL1A2,COL4A1,COL4A2,COL4A3,COL4A4,COL4A5,COL4A6,LOX,LOXL1,LOXL2,LOXL3,LOXL4,PCOLCE,PXDN,TLL1,TLL2	MSigDB
GOBP_POSITIVE_REGULATION_OF_ENDOTHELIAL_CELL_MATRIX_ADHESION_VIA_FIBRONECTIN	5	CEACAM6,FUT1,GFUS,RIN2,RRAS	MSigDB
GOBP_LYMPH_VESSEL_DEVELOPMENT	31	ACVR2B,ACVRL1,BMPR2,CCBE1,CLEC14A,EFNB2,EPHA2,FGF2,FLT4,FOXC1,FOXC2,HEG1,KDR,LGALS8,MIR9-1, NPR2, NR2F2, PDPN, PKD1, PPP3CB, PROX1, PTPN14,SOX18,SVEP1,SYK,TBX1,TIE1,TMEM204,VASH1,VEGFA,VEGFC	MSigDB
GOBP_LYMPH_VESSEL_MORPHOGENESIS	23	ACVR2B,ACVRL1,BMPR2,CCBE1,CLEC14A,EPHA2,FGF2,FLT4,FOXC1,FOXC2,LGALS8,MIR9-1,PDPN,PKD1,PPP3CB,PROX1,PTPN14,SOX18,SVEP1,TIE1,VASH1,VEGFA,VEGFC	MSigDB
GOBP_POSITIVE_REGULATION_OF_VASCULAR_ENDOTHELIAL_GROWTH_FACTOR_SIGNALING_PATHWAY	9	ADAMTS3,CCBE1,JCAD,MIR21,MYO1C,ROBO1,SMOC2,TNXB,VEGFA	MSigDB
GOBP_ENDOTHELIAL_CELL_MATRIX_ADHESION	8	ADAMTS9,CEACAM6,FUT1,GFUS,MIR92A1,MMP12,RIN2,RRAS	MSigDB
GOBP_POSITIVE_REGULATION_OF_ENDOTHELIAL_CELL_MIGRATION	132	AAMP,ABL1,ADAM17,ADGRA2,AGT,AKT1,AKT3,ANGPT1,ANGPT4,ANXA1,ANXA3,ATOH8,ATP5F1A,ATP5F1	MSigDB

		B,BCAR1,BCAS3,BMP4,BMPR2,CALR,CCBE1,CD40,CIB1,EDN1,EGF,EMC10,ETS1,FGF1,FGF16,FGF18,FGF2,FGFBP1,FGFR1,FLT4,FOXC2,FOXP1,FUT1,GATA2,GATA3,GFUS,GPI,GPLD1,GRN,HDAC7,HDAC9,HIF1A,HMGB1,HMOX1,HSPB1,ITGB1BP1,ITGB3,JCAD,KDR,LGMN,MAP2K3,MAP3K3,MAPK14,MET,MIR101-1,MIR10A,MIR10B,MIR126,MIR132,MIR135B,MIR143,MIR146A,MIR150,MIR1908,MIR199A1,MIR200A,MIR21,MIR210,MIR221,MIR23A,MIR27A,MIR27B,MIR296,MIR29A,MIR30A,MIR31,MIR342,MIR487B,MIR499A,MIR939,MIRLET7F1,NFE2L2,NOS3,NRP1,NRP2,NUS1,P2RX4,PDCD6,PDGFB,PDPK1,PIK3C2A,PIK3CB,PIK3CD,PIK3CG,PLCG1,PLK2,PLPP3,PRKCA,PRKD1,PRKD2,PROX1,PTGS2,PTK2B,RAC1,RHOB,RHOJ,RIN2,ROCK2,RRAS,SASH1,SCARB1,SEMA5A,SIRT1,SMOC2,SP1,SPARC,SRPX2,STAT5A,TDGF1,TEK,TGFB1,THBS1,TMSB4X,VEGFA,VEGFC,WNT5A,WNT7A,ZC3H12A,ZNF580	
GOBP_ENDOTHELIAL_CELL_CHEMOTAXIS	31	CCN3,CORO1B,CXCL13,EGR3,FGF1,FGF16,FGF18,FGF2,FGF4,FGFR1,HRG,HSPB1,KDR,LGMN,MET,MIR149,MIR16-1, MIR424, NOTCH1, NR4A1, NRP1, P2RX4, PLEKHG5,PRKD1,PRKD2,RAB13,SEMA5A,SMOC2,THBS1,TMSB4X,VEGFA	MSigDB
GOBP_POSITIVE_REGULATION_OF_ENDOTHELIAL_CELL_DIFFERENTIATION	19	ACVRL1,ATOH8,BMP4,BMP6,BTG1,CTNNB1,ETV2,GDF2,MIR150,MIR181A2,MIR181B1,MIR199B,MIR200C,MIR21,MIR34A,MIR99B,NOTCH1,TMEM100,VEZF1	MSigDB
GOBP_LYMPHATIC_ENDOTHELIAL_CELL_DIFFERENTIATION	8	ACVR2B,ACVRL1,BMPR2,NR2F2,PDPN,PROX1,SOX18,TIE1	MSigDB
GOBP_POSITIVE_REGULATION_OF_ENDOTHELIAL_CELL_DEVELOPMENT	6	ADD1,CDH5,CLDN5,F11R,PROC,S1PR2	MSigDB
KEGG_VASCULAR_SMOOTH_MUSCLE_CONTRACTION	115	ACTA2,ACTG2,ADCY1,ADCY2,ADCY3,ADCY4,ADCY5,ADCY6,ADCY7,ADCY8,ADCY9,ADORA2A,ADORA2B,ADRA1A,ADRA1B,ADRA1D,AGTR1,ARAF,ARHGEF1,ARHGEF11,ARHGEF12,AVPR1A,AVPR1B,BRAF,CACNA	MSigDB

		1C,CACNA1D,CACNA1F,CACNA1S,CALCRL,CALD1,CALM1,CALM2,CALM3,CALML3,CALML5,CALML6,CYP4A11,CYP4A22,EDNRA,GNA11,GNA12,GNA13,GNAQ,GNAS,GUCY1A1,GUCY1A2,GUCY1B1,IRAG1,ITPR1,ITPR2,ITPR3,JMJD7-PLA2G4B, KCNMA1, KCNMB1, KCNMB2,KCNMB3,KCNMB4,MAP2K1,MAP2K2,MAPK1,MAPK3,MYH11,MYL6,MYL6B,MYL9,MYLK,MYLK2,MYLK3,NPR1,NPR2,PLA2G10,PLA2G12A,PLA2G12B,PLA2G1B,PLA2G2A,PLA2G2C,PLA2G2D,PLA2G2E,PLA2G2F,PLA2G3,PLA2G4A,PLA2G4B,PLA2G4E,PLA2G5,PLA2G6,PLCB1,PLCB2,PLCB3,PLCB4,PPP1CA,PPP1CB,PPP1CC,PPP1R12A,PPP1R12B,PPP1R14A,PRKACA,PRKACB,PRKACG,PRKCA,PRKCB,PRKCD,PRKCE,PRKCG,PRKCH,PRKCQ,PRKG1,PRKX,PTGIR,RAF1,RAMP1,RAMP2,RAMP3,RHOA,ROCK1,ROCK2	
WP_ANGIOGENESIS	24	AKT1,ANGPT1,ARNT,CREBBP,FGF2,FGFR2,FLT1,HIF1A,KDR,MAPK1,MAPK14,MMP9,NOS3,PDGFB,PDGFRA,PIK3CA,PLCG1,PTK2,SMAD1,SRC,TEK,TIMP2,TIMP3,VEGFA	MSigDB
GOBP_POSITIVE_REGULATION_OF_FIBROBLAST_MIGRATION	17	ACTA2,AKAP12,AKT1,AQP1,ARHGEF7,BAG4,DDR2,DMTN,ITGB1,ITGB3,MIR145,PAK3,PRKCE,PTK2,SLC8A1,TGFB1,THBS1	MSigDB
GOMF_TYPE_2_FIBROBLAST_GROWTH_FACTOR_RECEPTOR_BINDING	5	FGF10,FGF17,FGF18,FGF7,FGF8	MSigDB
GOMF_FIBROBLAST_GROWTH_FACTOR_ACTIVATED_RECEPTOR_ACTIVITY	5	FGFR1,FGFR2,FGFR3,FGFR4,FGFRL1	MSigDB

eReference. References for Supplementary table 1.

1. Borghaei H, Gettinger S, Vokes EE, et al. Five-Year Outcomes From the Randomized, Phase III Trials CheckMate 017 and 057: Nivolumab Versus Docetaxel in Previously Treated Non-Small-Cell Lung Cancer. J Clin Oncol. 2021;39(7):723-733.
2. Mazieres J, Rittmeyer A, Gadgeel S, et al. Atezolizumab Versus Docetaxel in Pretreated Patients With NSCLC: Final Results From the Randomized Phase 2 POPLAR and Phase 3 OAK Clinical Trials. J Thorac Oncol. 2021;16(1):140-150.
3. Chang J, Wu YL, Lu S, et al. Three-year follow-up and patient-reported outcomes from CheckMate 078: Nivolumab versus docetaxel in a predominantly Chinese patient population with previously treated advanced non-small cell lung cancer. Lung Cancer. 2021;165:71-81.
4. Herbst RS, Garon EB, Kim DW, et al. Five Year Survival Update From KEYNOTE-010: Pembrolizumab Versus Docetaxel for Previously Treated, Programmed Death-Ligand 1-Positive Advanced NSCLC. J Thorac Oncol. 2021;16(10):1718-1732.
5. Okada M, Kato K, Cho BC, et al. Three-Year Follow-Up and Response-Survival Relationship of Nivolumab in Previously Treated Patients with Advanced Esophageal Squamous Cell Carcinoma (ATTRACTION-3). Clin Cancer Res. 2022;28(15):3277-3286.
6. Huang J, Xu J, Chen Y, et al. Camrelizumab versus investigator's choice of chemotherapy as second-line therapy for advanced or metastatic oesophageal squamous cell carcinoma (ESCORT): a multicentre, randomised, open-label, phase 3 study. Lancet Oncol. 2020;21(6):832-842.
7. Xu J, Li Y, Fan Q, et al. Clinical and biomarker analyses of sintilimab versus chemotherapy as second-line therapy for advanced or metastatic esophageal squamous cell carcinoma: a randomized, open-label phase 2 study (ORIENT-2). Nat Commun. 2022;13(1):857.
8. Shen L, Kato K, Kim SB, et al. Tislelizumab Versus Chemotherapy as Second-Line Treatment for Advanced or Metastatic Esophageal Squamous Cell Carcinoma (RATIONALE-302): A Randomized Phase III Study. J Clin Oncol. 2022;40(26):3065-3076.
9. Kojima T, Shah MA, Muro K, et al. Randomized Phase III KEYNOTE-181 Study of Pembrolizumab Versus Chemotherapy in Advanced Esophageal Cancer. J Clin Oncol. 2020;38(35):4138-4148.
10. Cohen EEW, Soulières D, Le Tourneau C, et al. Pembrolizumab versus methotrexate, docetaxel, or cetuximab for recurrent or metastatic head-and-neck squamous cell carcinoma (KEYNOTE-040): a randomised, open-label, phase 3 study. Lancet. 2019;393(10167):156-167.

11. Ferris RL, Blumenschein G Jr, Fayette J, et al. Nivolumab vs investigator's choice in recurrent or metastatic squamous cell carcinoma of the head and neck: 2-year long-term survival update of CheckMate 141 with analyses by tumor PD-L1 expression. Oral Oncol. 2018;81:45-51.
12. Ferris RL, Haddad R, Even C, et al. Durvalumab with or without tremelimumab in patients with recurrent or metastatic head and neck squamous cell carcinoma: EAGLE, a randomized, open-label phase III study. Ann Oncol. 2020;31(7):942-950.
13. Bang YJ, Ruiz EY, Van Cutsem E, et al. Phase III, randomised trial of avelumab versus physician's choice of chemotherapy as third-line treatment of patients with advanced gastric or gastro-oesophageal junction cancer: primary analysis of JAVELIN Gastric 300. Ann Oncol. 2018;29(10):2052-2060.
14. Shitara K, Özgüroğlu M, Bang YJ, et al. Pembrolizumab versus paclitaxel for previously treated, advanced gastric or gastro-oesophageal junction cancer (KEYNOTE-061): a randomised, open-label, controlled, phase 3 trial. Lancet. 2018;392(10142):123-133.
15. Bellmunt J, de Wit R, Vaughn DJ, et al. Pembrolizumab as Second-Line Therapy for Advanced Urothelial Carcinoma. N Engl J Med. 2017;376(11):1015-1026.
16. van der Heijden MS, Loriot Y, Durán I, et al. Atezolizumab Versus Chemotherapy in Patients with Platinum-treated Locally Advanced or Metastatic Urothelial Carcinoma: A Long-term Overall Survival and Safety Update from the Phase 3 IMvigor211 Clinical Trial. Eur Urol. 2021;80(1):7-11.
17. Hamid O, Puzanov I, Dummer R, et al. Final analysis of a randomised trial comparing pembrolizumab versus investigator-choice chemotherapy for ipilimumab-refractory advanced melanoma. Eur J Cancer. 2017;86:37-45.
18. Larkin J, Minor D, D'Angelo S, et al. Overall Survival in Patients With Advanced Melanoma Who Received Nivolumab Versus Investigator's Choice Chemotherapy in CheckMate 037: A Randomized, Controlled, Open-Label Phase III Trial. J Clin Oncol. 2018;36(4):383-390.
19. Pujade-Lauraine E, Fujiwara K, Ledermann JA, et al. Avelumab alone or in combination with chemotherapy versus chemotherapy alone in platinum-resistant or platinum-refractory ovarian cancer (JAVELIN Ovarian 200): an open-label, three-arm, randomised, phase 3 study. Lancet Oncol. 2021;22(7):1034-1046.
20. Hamanishi J, Takeshima N, Katsumata N, et al. Nivolumab Versus Gemcitabine or Pegylated Liposomal Doxorubicin for Patients With Platinum-Resistant Ovarian Cancer: Open-Label, Randomized Trial in Japan (NINJA). J Clin Oncol. 2021;39(33):3671-3681.
21. Spigel DR, Vicente D, Ciuleanu TE, et al. Second-line nivolumab in relapsed small-cell lung cancer: CheckMate 331. Ann Oncol. 2021;32(5):631-641.
22. Motzer RJ, Escudier B, McDermott DF, et al. Nivolumab versus Everolimus in Advanced Renal-Cell Carcinoma. N Engl J Med. 2015;373(19):1803-1813.

23. Winer EP, Lipatov O, Im SA, et al. Pembrolizumab versus investigator-choice chemotherapy for metastatic triple-negative breast cancer (KEYNOTE-119): a randomised, open-label, phase 3 trial. Lancet Oncol. 2021;22(4):499-511.
24. Eng C, Kim TW, Bendell J, et al. Atezolizumab with or without cobimetinib versus regorafenib in previously treated metastatic colorectal cancer (IMblaze370): a multicentre, open-label, phase 3, randomised, controlled trial. Lancet Oncol. 2019;20(6):849-861.
25. Popat S, Curioni-Fontecedro A, Dafni U, et al. A multicentre randomised phase III trial comparing pembrolizumab versus single-agent chemotherapy for advanced pre-treated malignant pleural mesothelioma: the European Thoracic Oncology Platform (ETOP 9-15) PROMISE-meso trial. Ann Oncol. 2020;31(12):1734-1745.
26. Reardon DA, Brandes AA, Omuro A, et al. Effect of Nivolumab vs Bevacizumab in Patients With Recurrent Glioblastoma: The CheckMate 143 Phase 3 Randomized Clinical Trial. JAMA Oncol. 2020;6(7):1003-1010.
