# Supplementary material for: Deciphering transcriptomic determinants of the divergent link between PD-L1 and immunotherapy efficacy
Source: NPJ Precis Oncol. 2023 Sep 11;7:87. doi: 10.1038/s41698-023-00443-3 (PMC10495439; doi:10.1038/s41698-023-00443-3)
Supplement: Supplementary file 2 — Reporting summary [file 41698_2023_443_MOESM2_ESM.pdf]

nature portfolio

Corresponding author(s): Shaodong Hong
Last updated by author(s): August 26, 2023

Reporting Summary

Nature Portfolio wishes to improve the reproducibility of the work that we publish. This form provides structure for consistency and transparency in reporting. For further information on Nature Portfolio policies, see our Editorial Policies and the Editorial Policy Checklist.

Please do not complete any field with "not applicable" or n/a. Refer to the help text for what text to use if an item is not relevant to your study.
For final submission: please carefully check your responses for accuracy; you will not be able to make changes later.

Statistics

For all statistical analyses, confirm that the following items are present in the figure legend, table legend, main text, or Methods section.

n/a	Confirmed	
☐	☒	The exact sample size (n) for each experimental group/condition, given as a discrete number and unit of measurement
☐	☒	A statement on whether measurements were taken from distinct samples or whether the same sample was measured repeatedly
☐	☒	The statistical test(s) used AND whether they are one- or two-sided<br>*Only common tests should be described solely by name; describe more complex techniques in the Methods section.*
☐	☒	A description of all covariates tested
☐	☒	A description of any assumptions or corrections, such as tests of normality and adjustment for multiple comparisons
☐	☒	A full description of the statistical parameters including central tendency (e.g. means) or other basic estimates (e.g. regression coefficient) AND variation (e.g. standard deviation) or associated estimates of uncertainty (e.g. confidence intervals)
☐	☒	For null hypothesis testing, the test statistic (e.g. F, t, r) with confidence intervals, effect sizes, degrees of freedom and P value noted<br>*Give P values as exact values whenever suitable.*
☒	☐	For Bayesian analysis, information on the choice of priors and Markov chain Monte Carlo settings
☒	☐	For hierarchical and complex designs, identification of the appropriate level for tests and full reporting of outcomes
☐	☒	Estimates of effect sizes (e.g. Cohen's d, Pearson's r), indicating how they were calculated

Our web collection on statistics for biologists contains articles on many of the points above.

Software and code

Policy information about availability of computer code

Data collection	No software was used to collect the data.
Data analysis	This paper does not report original code, except for the Cox proportional hazard interaction test between PD-L1 expression and candidate variables. This part of the code is deposited at https://github.com/NPTL1201/PDL1_predictiveness_interaction/blob/main/code_R.

For manuscripts utilizing custom algorithms or software that are central to the research but not yet described in published literature, software must be made available to editors and reviewers. We strongly encourage code deposition in a community repository (e.g. GitHub). See the Nature Portfolio guidelines for submitting code & software for further information.

Data

Policy information about availability of data

All manuscripts must include a data availability statement. This statement should provide the following information, where applicable:

- Accession codes, unique identifiers, or web links for publicly available datasets
- A description of any restrictions on data availability
- For clinical datasets or third party data, please ensure that the statement adheres to our policy

The clinical and RNA-seq data of OAK and POPLAR trials can be requested in European Genome-phenome Archive (Accession numbers EGAD00001008391, EGAD00001008390, EGAD00001008549, EGAD00001008548, EGAD00001008550, and EGAD00001006630). The clinical and RNA-seq data of IMvigor210 trial are accessible in R package IMvigor210CoreBiologies. The 28 clinical trial data were obtained from public literature. The TCGA Pan-Cancer cohort data were obtained from UCSC Xena browser and GDC PanImmune Data Portal, as noted in the Methods section.

Research involving human participants, their data, or biological material

Policy information about studies with human participants or human data. See also policy information about sex, gender (identity/presentation), and sexual orientation and race, ethnicity and racism.

Reporting on sex and gender	Data were formally requested from Genentech. Both male and female were included in this study.
Reporting on race, ethnicity, or other socially relevant groupings	Not applicable.
Population characteristics	The available population characteristics were described in Supplementary Tables 9-11.
Recruitment	Not applicable.
Ethics oversight	Not applicable. This study involves analyses of existing data.

Note that full information on the approval of the study protocol must also be provided in the manuscript.

Field-specific reporting

Please select the one below that is the best fit for your research. If you are not sure, read the appropriate sections before making your selection.

☒ Life sciences ☐ Behavioural & social sciences ☐ Ecological, evolutionary & environmental sciences

For a reference copy of the document with all sections, see nature.com/documents/nr-reporting-summary-flat.pdf

Life sciences study design

All studies must disclose on these points even when the disclosure is negative.

Sample size	1239 patients from three independent clinical trials (OAK: 699 patients; POPLAR: 192 patients; IMvigor210: 348 patients
Data exclusions	Detailed exlcusion criteria have been published in original publications.
Replication	We confirmed the consistent clinical and molecular observations in three clinical trials
Randomization	OAK and POPLAR are randomized trials and the randomization procedures have been described in original publications.
Blinding	Not applicable.

Reporting for specific materials, systems and methods

We require information from authors about some types of materials, experimental systems and methods used in many studies. Here, indicate whether each material, system or method listed is relevant to your study. If you are not sure if a list item applies to your research, read the appropriate section before selecting a response.

Materials & experimental systems

n/a	Involved in the study	
☒	☐	Antibodies
☒	☐	Eukaryotic cell lines
☒	☐	Palaeontology and archaeology
☒	☐	Animals and other organisms
☒	☐	Clinical data
☒	☐	Dual use research of concern
☒	☐	Plants

Methods

n/a	Involved in the study	
☒	☐	ChIP-seq
☒	☐	Flow cytometry
☒	☐	MRI-based neuroimaging
